# Structural analysis of *N*-glycans in chicken trachea and lung reveals potential receptors of chicken influenza viruses

**DOI:** 10.1038/s41598-022-05961-x

**Published:** 2022-02-08

**Authors:** Noriko Suzuki, Tatsuya Abe, Shunji Natsuka

**Affiliations:** 1 Graduate School of Science and Technology, Niigata University, 8050 Ikarashi-nino-cho, Nishi-ku, Niigata, 950-2181 Japan; 2 Faculty of Science, Niigata University, 8050 Ikarashi-nino-cho, Nishi-ku, Niigata, 950-2181 Japan

**Keywords:** Glycobiology, Glycomics

## Abstract

Although avian influenza A viruses (avian IAVs) bind preferentially to terminal sialic acids (Sia) on glycans that possess Siaα2-3Gal, the actual glycan structures found in chicken respiratory tracts have not been reported. Herein, we analyzed *N*-glycan structures in chicken trachea and lung, the main target tissues of low pathogenic avian IAVs. 2-Aminopyridine (PA)-labeled *N*-glycans from chicken tissues were analyzed by combined methods using reversed-phase liquid chromatography (LC), electrospray ionization (ESI)-mass spectrometry (MS), MS/MS, and multistage MS (MS^n^), with or without modifications using exoglycosidases, sialic acid linkage-specific alkylamidation (SALSA), and/or permethylation. The results of SALSA indicated that PA-*N*-glycans in both chicken trachea and lung harbored slightly more α2,6-Sia than α2,3-Sia. Most α2,3-Sia on *N*-glycans in chicken trachea was a fucosylated form (sialyl Lewis X, sLe^x^), whereas no sLe^x^ was detected in lung. By contrast, small amounts of *N*-glycans with 6-sulfo sialyl LacNAc were detected in lung but not in trachea. Considering previous reports that hemagglutinins (HAs) of avian IAVs originally isolated from chicken bind preferentially to α2,3-Sia with or without fucosylation and/or 6-sulfation but not to α2,6-Sia, our results imply that avian IAVs do not evolve to possess HAs that bind preferentially to α2,6-Sia, regardless of the abundance of α2,6-Sia.

## Introduction

Influenza A viruses (IAVs) cause zoonotic diseases and have a great impact on our lives. Beyond species-specific barriers for virus infections, they are transmitted from the natural hosts, wild waterfowl such as ducks, to other species of birds and mammals, including poultry, livestock, and humans^1^. Infection of domesticated chickens with avian IAVs is of significance for human lives, because chicken is one of the main poultry species worldwide, and a popular food source. Avian IAVs infecting chickens initially have low pathogenicity, but some evolve into highly pathogenic IAVs by mutations when they circulate among chickens. Highly pathogenic avian IAVs cause severe infections in poultry with high rates of mortality, resulting in serious economic damage.

Two spike glycoproteins expressed on the surface of IAVs, hemagglutinin (HA) and neuraminidase (NA), are involved in infection of host cells^2^. Binding of HA to sialic acid (Sia) residues on glycans expressed by target cells initiates virus attachment, thereby mediating the subsequent internalization step. When the amplified viruses are released from host cells, NA cleaves off the terminal Sia residues from host cells to prevent formation of virus aggregates at the budding site. The receptor binding specificities of HA are believed to be one of the main factors determining species tropism of IAVs, implying that glycans on host cells are natural barriers for transmission between different species. The well-known species-specific differences in HA specificities are that avian origin HAs bind preferentially to α2,3-Sia, whereas those of human origin mainly bind to α2,6-Sia^3^. Since α2,6-Sia is expressed predominantly on human upper airway epithelial cells^4^, it is thought that IAVs with HA that binds to α2,6-Sia are selected preferentially in humans. These specificities correlate with the amino acid sequences of HAs, and substitution of one or two amino acid residues in the receptor binding site of HAs can confer altered specificity^5^.

Species-specific differences in receptor binding specificities of HAs are also found among avian IAVs. While Siaα2-3Gal appears to be the minimum essential glycan structure for binding to HAs from avian IAVs, which infect either natural hosts or poultry, the fine details of the specificity of HAs differ depending on the original host species. For instance, comparison of the binding specificities of several HAs using synthesized glycan libraries revealed that duck-origin viruses displayed high affinity for glycans having NeuAcα2-3Galβ1-3GlcNAc/GalNAc rather than type II α2,3-sialyl LacNAc (NeuAcα2-3Galβ1-4GlcNAc), and that fucosylation and/or sulfation on these glycans resulted in weaker binding^6–8^. By contrast, some chicken-origin IAVs have stronger affinity for 6-sulfo α2,3-sialyl LacNAc (NeuAcα2-3Galβ1-4(SO_3_H-6)GlcNAc), sialyl Lewis X (sLe^x^, NeuAcα2-3Galβ1-4(Fucα1-3)GlcNAc), and/or 6-sulfo sLe^x^ (NeuAcα2-3Galβ1-4(Fucα1-3)(SO_3_H-6)GlcNAc) than for α2,3-sialyl LacNAc without fucosylation and sulfation, although the patterns of viral binding to the synthesized glycans varied significantly among viruses of different subtypes and among different isolates^6–10^. This fact implies that target glycan structures expressed on host cells may differ depending on the avian species, and induce acquisition of appropriate specificities through selective mutation on HAs.

Despite extensive studies on the glycan-binding specificities of HAs, information on the actual glycan structures on host cells in avian tissues is limited. Although histochemical staining of avian tissue samples using some lectins or anti-carbohydrate antibodies has been reported^9,11,12^, these methods can fail to identify the presence of some specific glycan structures that are not recognized by available lectins or antibodies. Recently, we developed a combined method for an *N*-glycomic analysis of tissue samples using chicken colon^13^, one of the main target tissues for infection by low pathogenic avian IAVs. Because IAVs are originally resident in the intestinal tract of natural hosts such as ducks^1^, they may initially infect a similar physiological environment in chickens. While intestines are mainly infected by orally-introduced IAVs, tissues in respiratory tracts such as trachea and lung, are the main target of IAVs entering through the nose^9^. Infection in these tissues seems to be one of the main route by which low pathogenic IAVs circulate among chickens in a poultry farm^14^, and such circulation occasionally induces viruses to evolve to highly pathogenic IAVs^15,16^. Therefore, information of the glycan structures present in these tissues is useful to find important structures that IAVs actually bind.

Herein, we report the glycan structures present in chicken trachea and lung to explore the actual glycan receptors of IAVs that influence changes in the glycan specificities of HAs. The results indicate that both the trachea and lung express slightly more Siaα2-6Gal than Siaα2-3Gal, although Siaα2-6Gal is not regarded as a preferred receptor of avian IAVs. Moreover, the chicken trachea expresses large amounts of fucosylated glycans, including Le^x^ (Galβ1-4(Fucα1-3)GlcNAc), sLe^x^, and sulfo fucosyl LacdiNAc on branches, whereas only a trace amount of Le^x^, and no sLe^x^, was found in chicken lung.

## Results

### *N*-Glycomic analysis of chicken trachea and lung by LC–MS and MS/MS

For glycan structural analysis with LC–MS and MS/MS, we performed two independent experiments to prepare *N*-glycans from both chicken trachea and lung. The raw data of LC–MS and MS/MS have been deposited to GlycoPOST (https://glycopost.glycosmos.org)^17^. The results from the two independent experiments were consistent with respect to glycan structural features; therefore, we describe just one of the experiments below.

**Figure 1:**
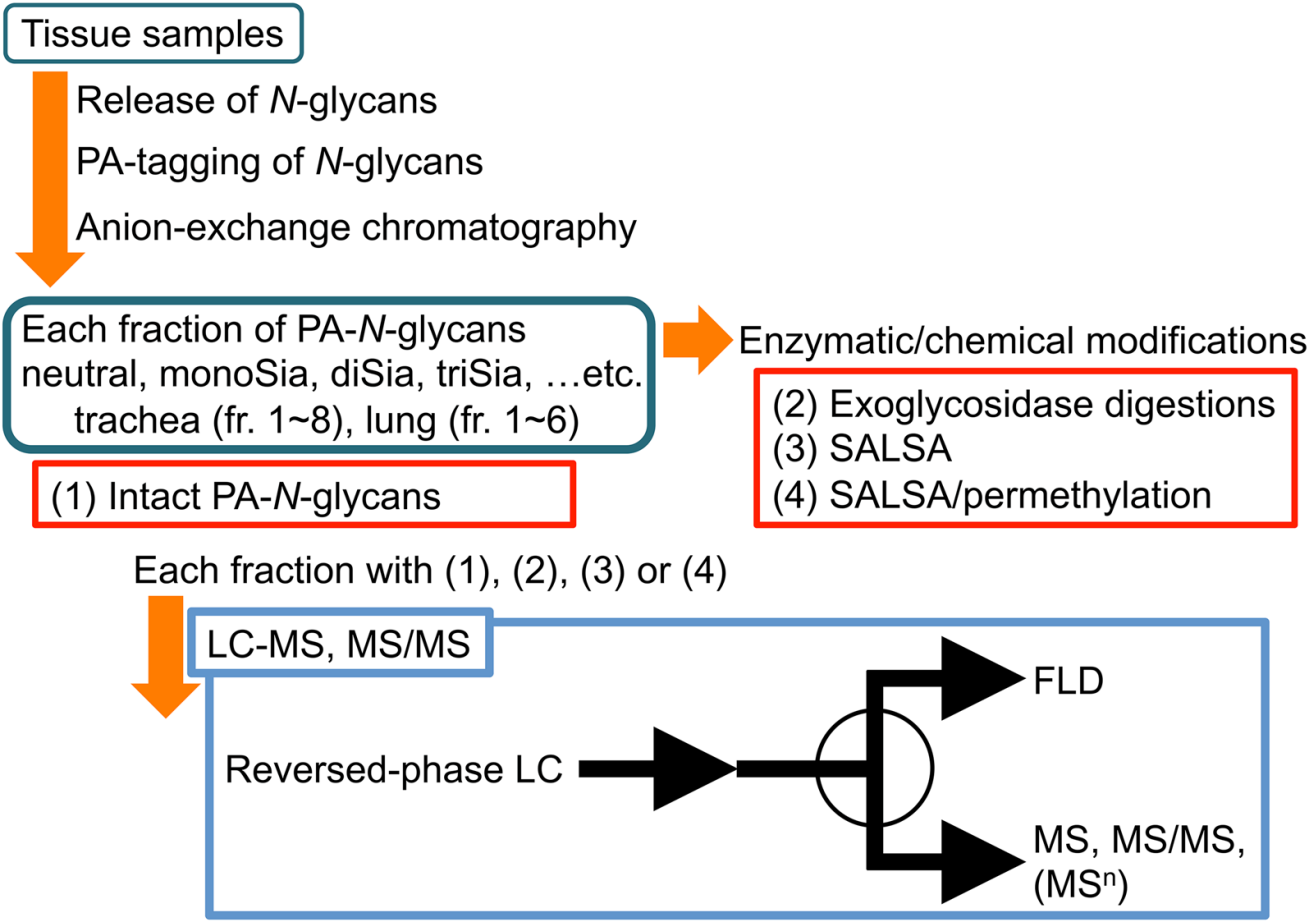
Workflow of procedural steps for structural analysis of *N*-glycans from chicken tissues. *N*-Glycans are released enzymatically from tissue glycoproteins, and labeled with PA. After partial fractionation of PA-*N*-glycans based on their negative charge on an anion-exchange column, each fraction, which contains intact PA-*N*-glycans (1), is subjected to LC–MS and MS/MS analysis using a reversed-phase column to obtain an overview of the structural features of tissue glycans. Eluents from the column are separated evenly (1:1) using a splitter, and PA-glycans are detected simultaneously using a MS system and a fluorescence detector (FLD). Glycan structures, including anomeric configurations, are confirmed by a combination of exoglycosidase digestions (2). The branching/core structures of *N*-glycans can be confirmed based on their elution positions, as well as LC–MS and MS/MS analysis, after exoglycosidase digestions. Linkages of sialic acids on sialylated glycans are determined and quantified by SALSA (3), followed by LC–MS and MS/MS analysis. Permethylation of alkylamidated glycans (4) followed by LC–MS, MS/MS, and MS^n^ analysis accompanied by cross-ring cleavages is useful for further determination of glycosidic linkage positions in some glycans.

*N*-Glycans from chicken trachea and lung derivatized with 2-aminopyridine (PA) were separated into eight and six fractions, respectively, by high-performance liquid chromatography (HPLC) using a diethylaminoethyl (DEAE) anion-exchange column (Fig. 1, Supplementary Fig. S1A, S1C). The results of LC–MS revealed that seven of the eight fractions (fr. 1, 3–8) from trachea and five of the six fractions (fr. 1, 3–6) from lung separated on the DEAE column contained PA-*N*-glycans (Supplementary Fig. S1B, S1D). MS/MS spectra indicated some fragment ions such as *m/z* 366 (Hex_1_HexNAc_1_), *m/z* 657 (Hex_1_HexNAc_1_NeuAc_1_), *m/z* 512 (Hex_1_HexNAc_1_Fuc_1_), *m/z* 803 (Hex_1_HexNAc_1_Fuc_1_NeuAc_1_), *m/z* 407 (HexNAc_2_), and *m/z* 731 (Hex_2_HexNAc_2_), which are characteristic B ion fragments derived from complex- or hybrid-type *N*-glycans, and contributed to the estimation of approximate branch sequences (Supplementary Table S1A, S1B). For convenience, deoxyhexoses are indicated as fucose (Fuc) in this study. Small amounts of artifactual by-products generated by C-2 epimerization of reducing termini, i.e., conversion from GlcNAc to ManNAc, were eluted earlier in reversed-phase HPLC than the corresponding non-epimerized PA-*N*-glycans as described previously^18^. In this study, C-2 epimers are simply indicated as “epimer” in Fig. S4 and Table S1. The estimated monosaccharide compositions suggested the presence of highly branched structures, such as tri-, tetra-, or penta-antennary *N*-glycans, in lung but not trachea (Supplementary Table S1A, S1B). Using some PA-*N*-glycans from glycoproteins as reference standards (Supplementary Fig. S2), the core and branching structures (Supplementary Fig. S3) of the tissue *N*-glycans were deduced based on the elution positions, MS, and MS/MS spectra.

**Figure 2:**
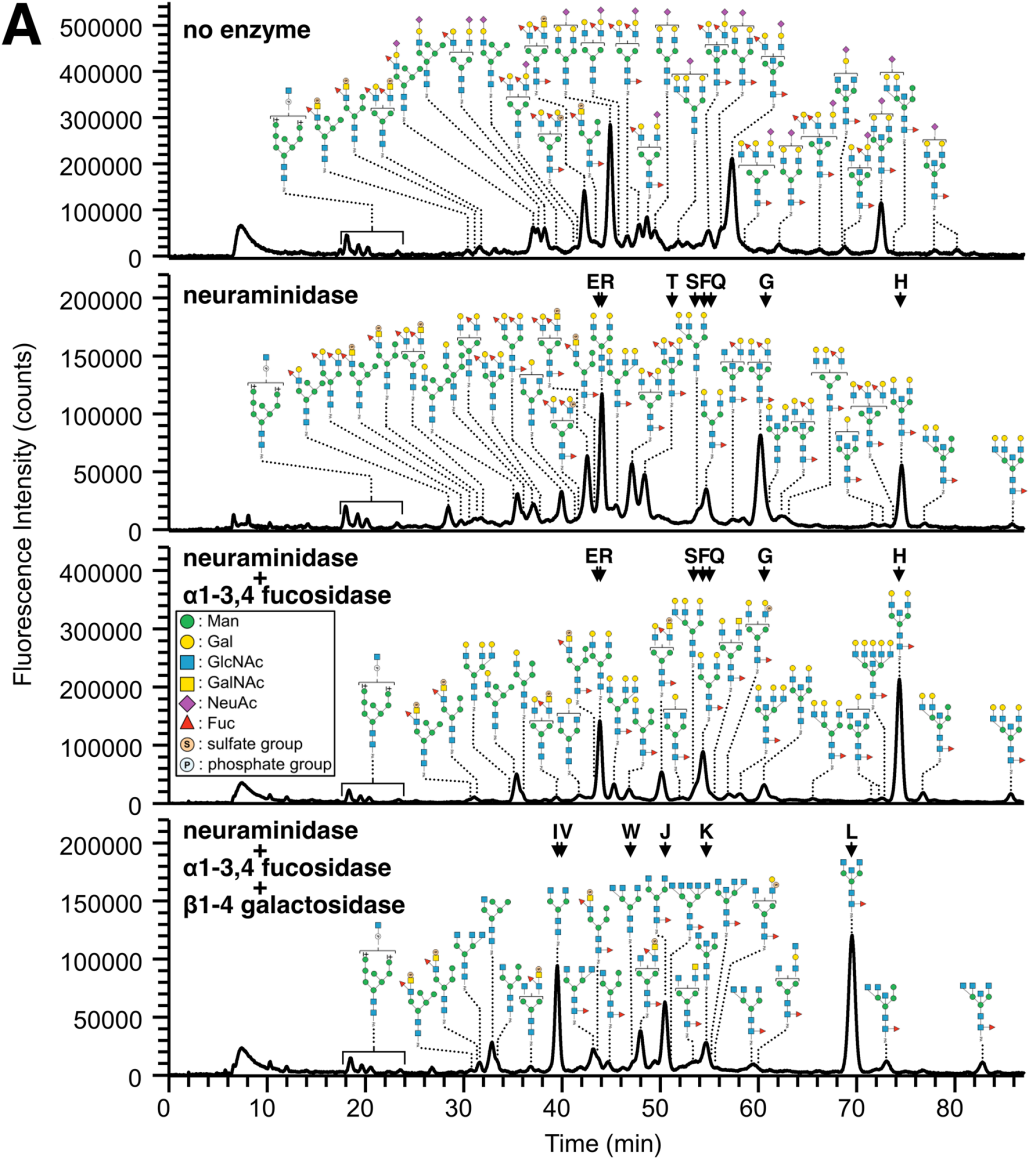

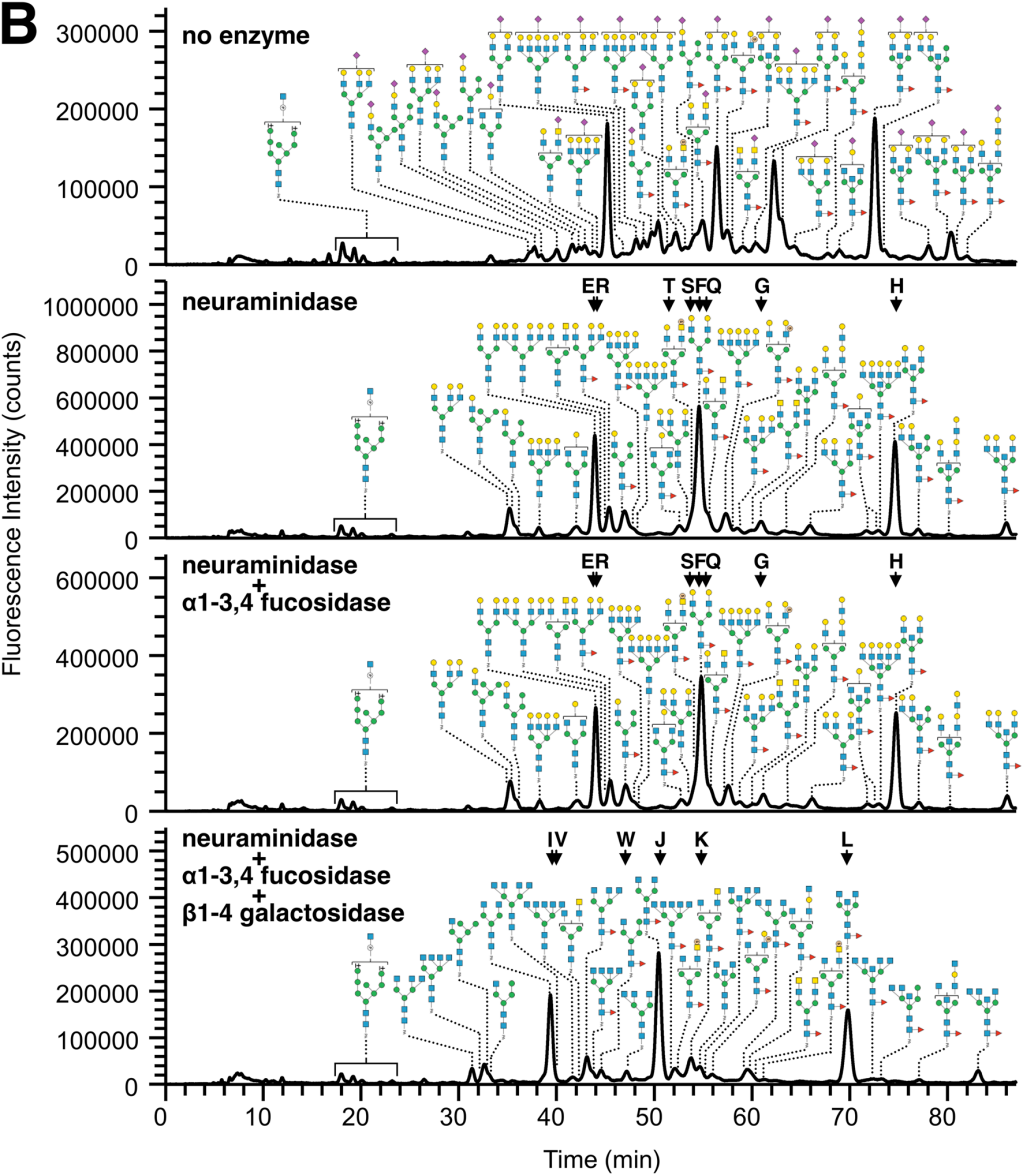
Elution profiles of PA-*N*-glycans from chicken trachea and lung after sequential digestion with exoglycosidases. A portion of fr. 3 from the DEAE column (monosialylated PA-*N*-glycans, see Supplementary Fig. S1A,C) derived from chicken trachea (**A**) and lung (**B**) was sequentially digested with neuraminidase, α1-3,4 fucosidase, and β1-4 galactosidase, and each digest was subjected to LC–MS and MS/MS analyses. Arrows with alphabetical characters indicate the elution positions of the standard PA-*N*-glycans (Supplementary Fig. S2). Some representative PA-*N*-glycans, but not all the structures detected by MS/MS, are shown in this figure. Standard Symbol Nomenclature for Glycans was used for monosaccharide symbols^30^, except for sulfate and phosphate groups.

A portion of each fraction of PA-*N*-glycans was subjected to sequential exoglycosidase digestion using neuraminidase, α1-3,4 fucosidase, and β1-4 galactosidase to clarify the sequences of branches and branching patterns. Figure 2 shows a representative example of elution profiles on reversed-phase LC following exoglycosidase digestion of PA-*N*-glycans in fr. 3 from chicken trachea and lung, which contained monosialylated or monosulfated glycans. The elution profiles of PA-*N*-glycans in fr. 3 from both trachea and lung were altered markedly by neuraminidase digestion. By contrast, the elution profiles following α1-3,4 fucosidase digestion of PA-*N*-glycans from chicken trachea were also altered significantly, whereas those from lung were mostly unchanged. These results suggest that chicken trachea expresses abundant α3/4-Fuc on *N*-glycans, unlike lung. After β1-4 galactosidase digestion, the elution profiles of both trachea and lung were also altered markedly, suggesting that the majority of complex/hybrid-type *N*-glycans possess type II LacNAc (Galβ1-4GlcNAc). In the case of lung, some minor PA-*N*-glycans retained one LacNAc sequence, even after treatment with an appreciable amount of β1-4 galactosidase (e.g., Fig. S4-10, fr. 3/4 with β1-4 galactosidase, at *m/z* 1258.49(2H^+^) eluted around 71.51 min), implying the presence of type I LacNAc (Galβ1-3GlcNAc) as a minor component.

### MS analysis of characteristic structures of *N*-glycans in trachea and/or lung using combined methods

The detailed structural features such as glycosidic linkages and branching patterns of *N*-glycans in trachea and/or lung were analyzed by the combining sequential exoglycosidase digestion, sialic acid linkage-specific alkylamidation (SALSA), and/or permethylation, as well as the elution position on reversed-phase LC. Using the SALSA method, α2,3-Sia and α2,6-Sia were alkylamidated by methylamine (MA, + 13.032) and isopropylamine (iPA, + 41.063), respectively, resulting in a mass difference (Δ = 28.031)^19^. This mass difference is maintained even after permethylation^20^. To deduce the glycan structures, we also used empirical additivity rules, in which the type and position of each constituent monosaccharide additionally contribute positively or negatively to the retention of PA-*N*-glycans in LC^18,21^. Some characteristic structures of PA-*N*-glycans were analyzed as follows:

#### Fucosylated structures

The results of LC–MS and MS/MS analyses indicated the presence of multiply fucosylated *N*-glycans in trachea. The positions of Fuc residues, either on branches or the *N*-glycan core, were deduced by comparison of the elution positions in reversed-phase LC as described previously^13^. For instance, extracted ion chromatograms (EICs) of neuraminidase–treated fr. 3 from trachea revealed the different elution positions of fucosylated biantennary structures without bisecting GlcNAc, assigned as Hex_2_HexNAc_2_Fuc_1–3_C-PA (C is the trimannosyl core structure, Man_3_GlcNAc_2_). As shown in Supplementary Fig. S4-1A, the results indicate that the addition of one core Fuc made a strong positive contribution to retention (10–11 min), whereas addition of one α1,3-Fuc residue on a branch made a strong negative contribution to retention (6–8 min), when the PA-*N*-glycans do not possess bisecting GlcNAc. The results of MS/MS support the presence of Fuc residues on branches, which are more easily removed by fragmentation with collision-induced dissociation (CID), as shown in Supplementary Fig. S4-1B. Furthermore, the presence of Le^x^ was confirmed by MS^3^ analysis of permethylated neutral PA-*N*-glycan possessing Fuc residues on branches (Supplementary Fig. S5-1).

**Figure 3:**
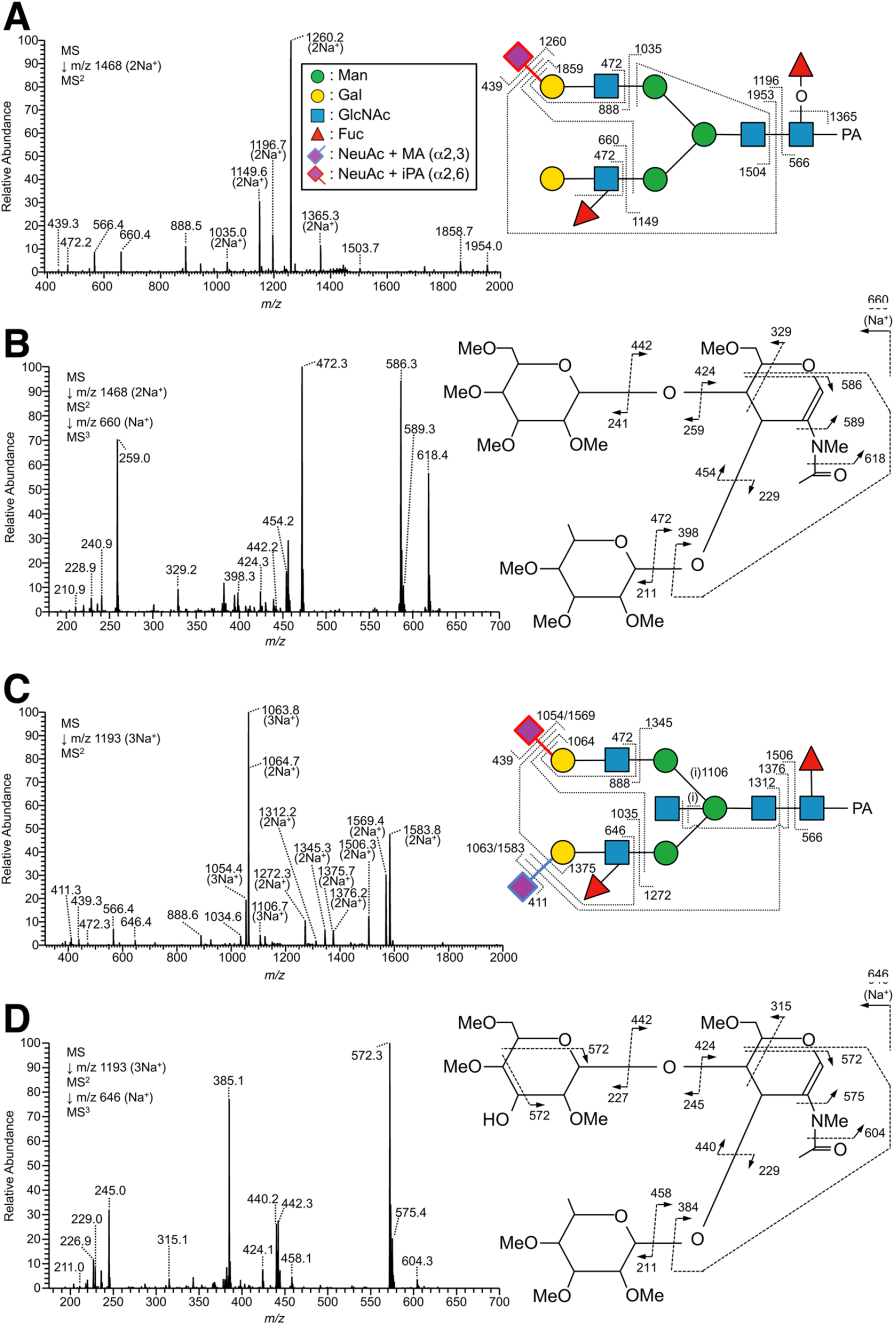
MS^n^ analysis of alkylamidated and permethylated PA-*N*-glycans from chicken trachea. (**A**) MS^2^ spectrum of PA-*N*-glycans with Le^x^ from the doubly sodiated precursor ion at *m/z* 1468, corresponding to Hex_2_HexNAc_2_Fuc_2_(NeuAc + iPA)_1_C-PA. The branch positions of each glycan sequence linked to α3- or α6-Man were not determined. (**B**) MS^3^ spectrum of the B ion fragments Hex_1_HexNAc_1_Fuc_1_ at *m/z* 660. (**C**) MS^2^ spectrum of PA-*N*-glycans with sLe^x^ from the triply sodiated precursor ion at *m/z* 1193, corresponding to Hex_2_HexNAc_3_Fuc_2_(NeuAc + MA)_1_(NeuAc + iPA)_1_C-PA. The branch positions of each glycan sequence linked to α3- or α6-Man were not determined. (**D**) MS^3^ spectrum of the B/Y ion fragments Hex_1_HexNAc_1_Fuc_1_ at *m/z* 646.

Some sialylated PA-*N*-glycans from trachea possessed Fuc residues either on branches or the *N*-glycan core (Supplementary Fig. S4-2A). The linkages of Sia to Gal, and the positions of Fuc residues, were deduced based on the elution positions and MS/MS spectra (Supplementary Fig. S4-2B, S4-2C), and then confirmed by SALSA (Supplementary Table S2A, S2B) and a combined SALSA/permethylation method^20^. For instance, the EIC at *m/z* 1078.91 of PA-*N*-glycans in fr.3 from trachea, assigned as Hex_2_HexNAc_2_Fuc_1_NeuAc_1_C-PA(2H^+^), exhibited several isomer peaks (Supplementary Fig. S4-2A). Similar to the neuraminidase-treated samples, sialylated PA-*N*-glycans with Fuc residues on a branch were eluted earlier than those with core Fuc. Previously, we found that in general, biantennary PA-*N*-glycans with Siaα2-6Gal were eluted earlier than those with Siaα2-3Gal^13^, and this rule seemed to be applicable in the presence of branch Fuc residues. The results of SALSA/permethylation revealed that branches that contain the Siaα2-6Gal sequence were not fucosylated at the same branches. When PA-*N*-glycans contain both Fuc residues on a branch and a Siaα2-6Gal sequence, these moieties are located on different branches (Fig. 3A,B). By contrast, one Fuc residue sometimes coexists on the same branch that possesses Siaα2-3Gal sequences (Fig. 3C,D, Supplementary Fig. S5-2), suggesting the presence of sLe^x^ or sialyl Lewis A (sLe^a^, NeuAcα2-3Galβ1-3(Fucα1-4)GlcNAc). Because the Gal-GlcNAc linkages were almost completely removed by β1-4 galactosidase digestion (Fig. 2A), the branches are most likely sLe^x^, but not sLe^a^. This branch sequence was further confirmed by SALSA/permethylation and MS^3^ analyses accompanied by cross-ring cleavages of B/Y fragments at *m/z* 646 (Fig. 3C,D, Supplementary Fig. S5-2). A small peak at *m/z* 315 derived from ^3,5^A cross-ring cleavage of HexNAc (Fig. 3D, Supplementary Fig. S5-2D) was generated, suggesting linkages of Hex1–4HexNAc and dHex1–3HexNAc, which are the part of sLe^x^, as described previously^13,20^.

#### Sulfated structures

Sulfated *N*-glycans were found in both trachea and lung, but the branch sequences that possess a sulfate group varied. In the case of trachea, sulfated fucosyl LacdiNAc were the main *N*-glycans, with one or two sulfate groups (Supplementary Fig. S5-3, Supplementary Table S1A), and sulfated fucosyl LacNAc, most likely sulfo Le^x^ (Galβ1-4(Fucα1-3)(SO_3_H-6)GlcNAc), was also detected as a minor component (Supplementary Fig. S5-4). MS^3^ analysis of the permethylated glycans suggested that the position of the sulfate group on the sulfated fucosyl LacdiNAc was the 4- or 6-OH of the HexNAc at the non-reducing terminus, which is most likely GalNAc (Supplementary Fig. S5-3). By contrast, based on the results of MS/MS analysis of the permethylated glycans as well as LC–MS and MS/MS analyses after sequential exoglycosidase digestion, the position of a sulfate group on the sulfated fucosyl LacNAc was most likely the 6-OH of the inner GlcNAc (Supplementary Fig. S5-4, Fig. S6). It should be noted that sulfated *N*-glycans from trachea did not possess Sia residues on the same branches. Some *N*-glycans possessed sulfate groups and Sia simultaneously (e.g., pk. 6-8-1 and pk. 6-10-1 in Supplementary Table S1A), but they are located at different branches.

**Figure 4:**
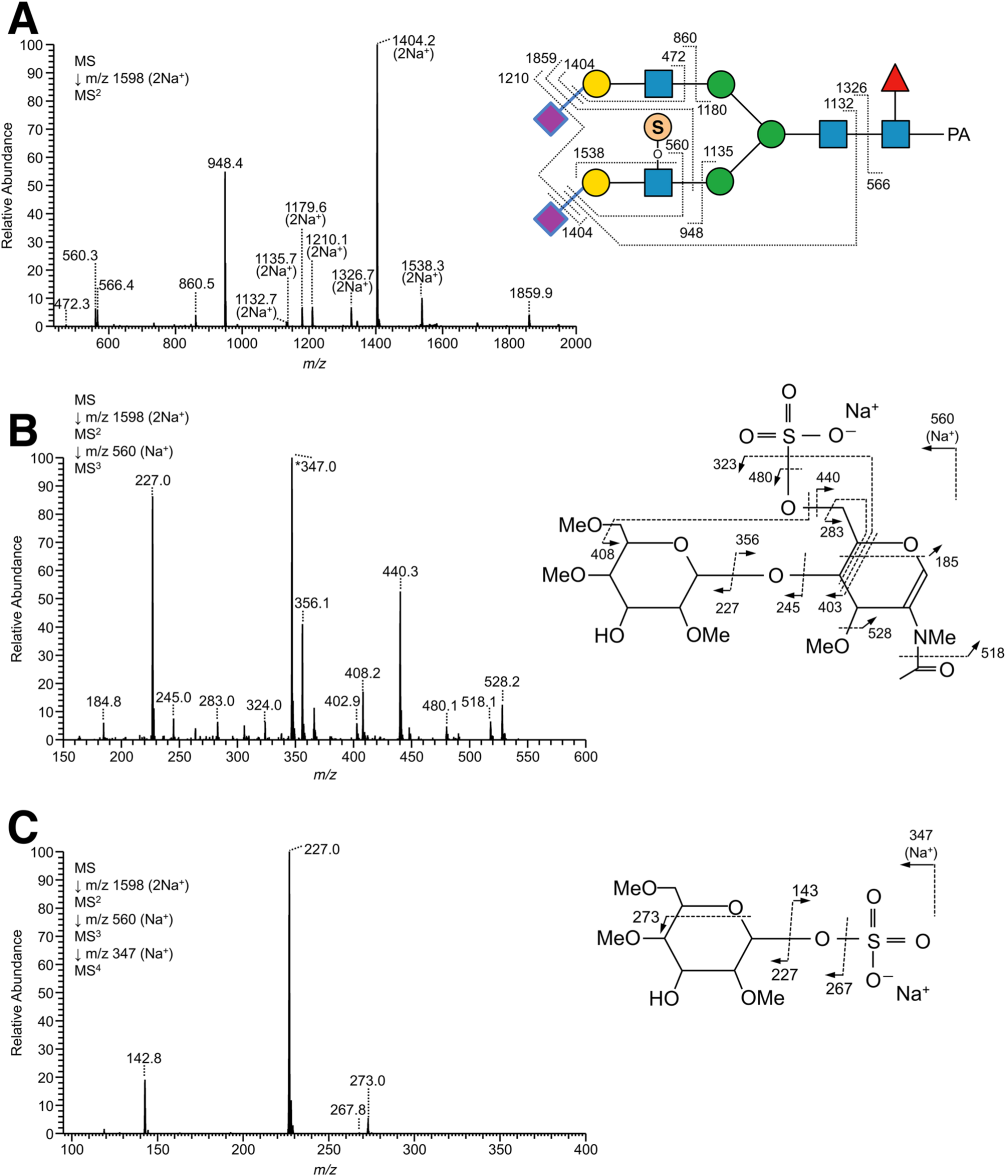
MS^n^ analysis of alkylamidated and permethylated PA-*N*-glycans with sulfation derived from chicken lung. (**A**) MS^2^ spectrum of α2,3-sialylated PA-*N*-glycans with sulfated LacNAc from the doubly sodiated precursor ion at *m/z* 1598, corresponding to Hex_2_HexNAc_2_Fuc_1_(NeuAc + MA)_2_(SO_3_-H + Na)_1_C-PA. (**B**) MS^3^ spectrum of the B/Y ion fragments Hex_1_HexNAc_1_(SO_3_-H + Na)_1_ at *m/z* 560. The peak at *m/z* 347, indicated by the asterisk (*), was probably generated by rearrangement of sodium sulfate (–SO_4_Na) on HexNAc to the B/Y ion fragments at *m/z* 227 derived from Hex on the non-reducing end, according to the result of MS^4^ analysis at *m/z* 347 (**C**). Although the sulfate groups on the glycan structures are represented as if they were located on a branch linked to α3-Man (**A**), the actual branch positions were not determined.

Unlike trachea, lung contained *N*-glycans with either sulfated LacdiNAc or sulfated LacNAc without Fuc residues on branches. MS^3^ analysis of the permethylated glycans suggested that the position of sulfate groups on the sulfated LacdiNAc was the 4- or 6-OH of the HexNAc at the non-reducing terminus, which is most likely GalNAc (Supplementary Fig. S5-5). By contrast, the position of a sulfate group on the sulfated LacNAc was most likely the 6-OH of the inner GlcNAc, based on the results of MS/MS analysis of permethylated glycans as well as LC–MS and MS/MS analyses (Fig. 4). It should be noted that sulfated *N*-glycans from lung sometimes possessed Sia residues on the same branches, such as NeuAcα2-3Galβ1-4(SO_3_H-6)GlcNAc and NeuAcα2-6Galβ1-4(SO_3_H-6)GlcNAc. The linkages of Sia were deduced based on the results of SALSA (Supplementary Table S2B), as well as the elution positions before and after neuraminidase digestion (Supplementary Fig. S6).

#### LacdiNAc and branching patterns

The presence of multiantennary structures of *N*-glycans from lung was suggested by the deduced monosaccharide composition: Hex_*n*_HexNAc_*n*_Fuc_0–1_NeuAc_0–3_C-PA or Hex_*n*_HexNAc_(*n*+1)_Fuc_0–1_NeuAc_0–3_C-PA (*n* = 2–6). Elution positions of PA-*N*-glycans with different branching patterns were compared using EICs of chicken lung PA-*N*-glycans with varying compositions (HexNAc_4–5_Fuc_0–1_C-PA) prepared by neuraminidase/α1-3,4 fucosidase/β1-4 galactosidase digestion (Supplementary Fig. S4-3A, S4-4A, S4-5A, S4-6A). As we reported previously^13^, PA-*N*-glycans can be separated by reversed-phase LC using a C18 column based on the branching pattern. For example, two different types of triantennary structures, designated 2,2′,6′-tri and 2,4,2′-tri (Supplementary Fig. S3), eluted much earlier and later, respectively, than the cognate 2,2′-biantennary structure. These differences are attributable to the negative contribution of branching GlcNAc linked to the C-6 position of α6-Man (β6′-GlcNAc, 7–8 min), and the positive contribution of branching GlcNAc linked to the C-4 position of α3-Man (β4-GlcNAc, 7–9 min), to retention of PA-*N*-glycans without bisecting GlcNAc. We also found that branching GlcNAc linked to the C-4 position of α6-Man (β4′-GlcNAc) made a small positive contribution (1–2 min) in the case of PA-*N*-glycans, without bisecting GlcNAc. Accordingly, we deduced PA-*N*-glycans eluted at 32.55 min (Supplementary Fig. S4-3A, fr. 1, EIC at *m/z* 901.36, HexNAc_4_C-PA(2H^+^)) as a 2,2′,4′,6′-tetraantennary structure. Based on the elution position and MS/MS spectra, the peak at 35.17 min in the same EIC is most likely a 2,2′,6′-triantennary structure with one LacdiNAc, which is characterized by a B ion fragment at *m/z* 407 upon MS/MS analysis (Supplementary Fig. S4-3B). The elution positions and MS/MS spectra of PA-*N*-glycans eluted at 39.62, 42.94, and 72.15 min on the EIC at *m/z* 901.36 (Supplementary Fig. S4-3B) were the same as those from chicken colon^13^, suggesting that each of their structures is the same.

Each structure of PA-*N*-glycans eluted at the indicated time on EICs at *m/z* 974.39 (HexNAc_4_Fuc_1_C-PA(2H^+^), Supplementary Fig. S4-4A), at *m/z* 1002.90 (HexNAc_5_C-PA(2H^+^), Supplementary Fig. S4-5A), and at *m/z* 1075.93 (HexNAc_5_Fuc_1_C-PA(2H^+^), Supplementary Fig. S4-6A), were similarly deduced based on elution position and MS/MS spectra (Supplementary Fig. S4-4B, S4-5B, and S4-6B). The presence of LacdiNAc (GalNAc-GlcNAc) was confirmed by the hallmark B ion fragments at *m/z* 407. These results suggest that addition of the second HexNAc (most likely GalNAc) to the first HexNAc (most likely GlcNAc) of HexdiNAc resulted in a positive contribution to retention in the range of 3–6 min, and that this contribution differed slightly depending on the arm on which the HexNAc was added, as reported previously^13^. The presence of LacdiNAc and sialyl LacdiNAc (sLacdiNAc) in chicken lung was confirmed by SALSA/permethylation (Supplementary Fig. S5-6). The results indicated that sLacdiNAc possessed α2,6-Sia but not α2,3-Sia. Interestingly, both 2,4,2′,4′,6′-pentaantennary structures (e.g., eluted at 41.31 min (fr. 1) in Fig. S4-5, eluted at 52.05 min (fr. 1) in Fig. S4-6) and 2,2′,4′,6′-tetraantennary structures (e.g., eluted at 32.55 min (fr. 1) in Fig. S4-3, eluted at 43.66 min (fr. 1) in Fig. S4-4), which are rarely found in mammals, were relatively abundant in chicken lung (Supplementary Fig. S4-3A, S4-4A, S4-5A, S4-6A).

#### LacNAc repeats and multiantennary structures with LacNAc

The presence of multiantennary structures and/or extended LacNAc repeat sequences in chicken lung was supported by the deduced compositions of complex-type PA-*N*-glycans with Hex_*n*_HexNAc_*n*_Fuc_0–1_NeuAc_0–5_C-PA or Hex_*n*_HexNAc_(*n*+1)_Fuc_0–1_NeuAc_0–4_C-PA (*n* = 2–6). The branching patterns were deduced based on the elution positions observed in the EICs of PA-*N*-glycans with the compositions of Hex_*n*_HexNAc_*n*_Fuc_0–1_C-PA(3H^+^) or Hex_*n*_HexNAc_(*n*+1)_Fuc_0–1_C-PA(3H^+^) (*n* = 4–6) using data from LC–MS and MS/MS analyses of each neuraminidase/α1-3,4 fucosidase–digested fraction (Supplementary Fig. S4-7A, S4-8A, S4-9A, S4-10A). The EICs of their β1-4 galactosidase–digested products (Supplementary Fig. S4-10A) were also examined to confirm the number of remaining Hex (Gal) residues. PA-*N*-glycans with multiantennary structures (including pentaantennary structures) and/or LacNAc repeats were successfully separated, and their structures were deduced based on the elution positions and MS/MS spectra (Supplementary Fig. S4-7B, S4-8B, S4-9B, S4-10B). For example, EICs at *m/z* 866.00 [Hex_4_HexNAc_4_Fuc_1_C-PA(3H^+^)] revealed that PA-*N*-glycans with this composition were clearly separated into fully galactosylated 2,2′-bi- and 2,2′,6′-tri-antennary structures with LacNAc repeats, as well as 2,2′,4′,6′-tetra- and 2,4,2′,4′-tetra-antennary structures (Supplementary Fig. S4-7A). The PA-*N*-glycan that eluted around 67.71 min (fr. 3) generated B ion fragments at *m/z* 1096 (Hex_3_HexNAc_3_) and *m/z* 731 (Hex_2_HexNAc_2_), suggesting biantennary structures with a branch containing three LacNAc units connected in tandem (Supplementary Fig. S4-7B). EICs at *m/z* 1109.42 [Hex_6_HexNAc_6_Fuc_1_C-PA(3H^+^)] of PA-*N*-glycans revealed the presence of 2,4,2′,4′,6′-penta- and 2,4,2′,4′-tetra-antennary structures with one and two LacNAc repeats, respectively (Supplementary Fig. S4-10). After the β1-4 galactosidase digestion, the former lost five of the six Gal residues, and the latter lost four of them, confirming the number of LacNAc repeat sequences.

### Comparison of structural features of *N*-glycans from chicken trachea and lung

**Figure 5:**
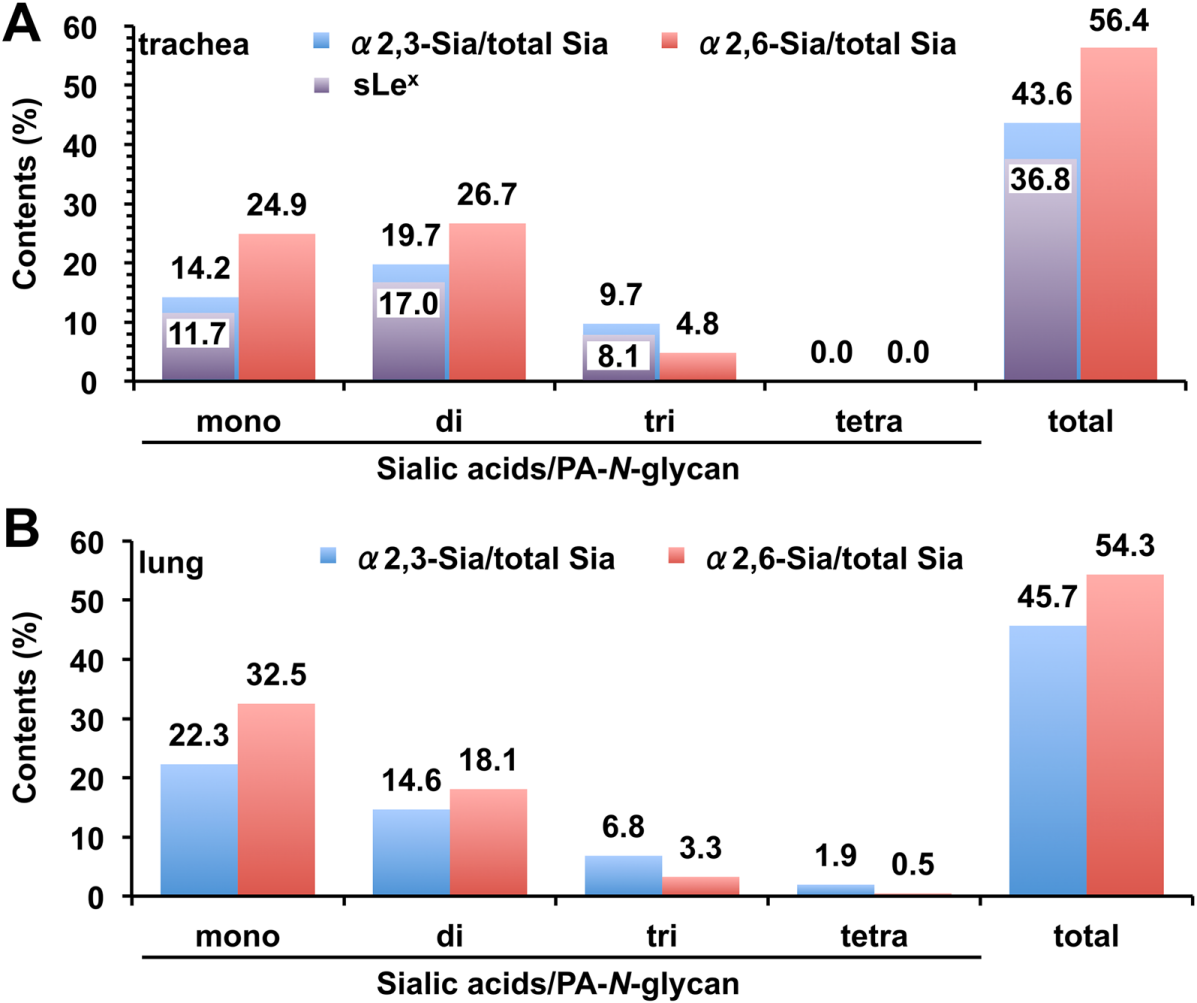
Relative content of α2,3- and α2,6-Sia in sialylated *N*-glycans from chicken trachea (**A**) and lung (**B**), estimated using the SALSA method. The proportions of α2,3- and α2,6-Sia in mono-, di-, tri-, or tetra-sialylated glycans, as well as in total glycans from the tissues, were calculated based on the results of LC–MS and MS/MS analyses of PA-*N*-glycans after SALSA, as indicated in Supplementary Table S2. In the results of trachea, the relative content of sLe^x^ is shown as purple bars (**A**).

A portion of each fraction containing sialylated PA-*N*-glycans from chicken trachea (fr. 3–6, 8) and lung (fr. 3–6) were chemically modified by SALSA to discriminate α2,3- or α2,6-Sia in PA-*N*-glycans, as described previously^19,22^, and then analyzed by LC–MS and MS/MS (Supplementary Fig. S7). Based on the results of full MS and MS/MS analyses, we deduced the monosaccharide compositions and Sia-linkages of each PA-*N*-glycan detected by a fluorescence detector (FLD) (Supplementary Table S2A, S2B). The proportions of α2,3- and α2,6-Sia at non-reducing termini of PA-*N*-glycans from trachea and lung were estimated using the peak area of each PA-*N*-glycan derivatized by the SALSA method (Fig. 5). The results revealed that the proportion of α2,3- and α2,6-Sia in sialylated branches of PA-*N*-glycans was 43.6% and 56.4%, respectively, in trachea, and 45.7% and 54.3%, respectively, in lung, indicating that the proportion of α2,3-Sia was slightly lower than that of α2,6-Sia in both tissues. It should be noted that the proportion of α2,3-Sia in both trachea and lung was slightly lower than that of α2,6-Sia on mono- and di-sialylated PA-*N*-glycans, whereas the proportion of α2,3-Sia was slightly higher than that of α2,6-Sia on tri- or tetra-sialylated PA-*N*-glycans. These results reflect the abundance of α2,6-Sia on biantennary structures. It is also notable that the majority of α2,3-Sia in trachea is further fucosylated, i.e., present as sLe^x^ (Fig. 5A).

**Figure 6:**
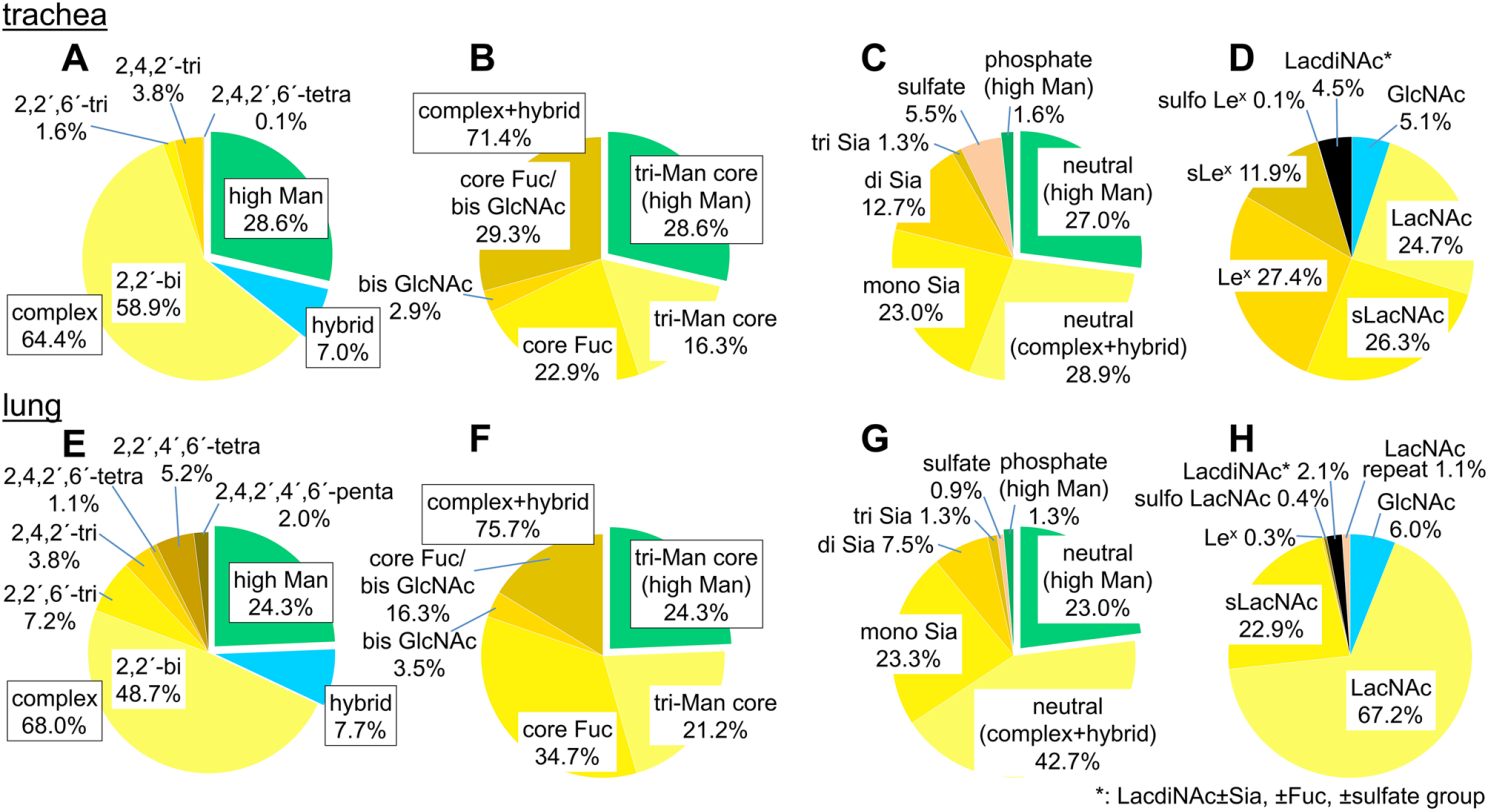
Summary of the structural features of *N*-glycans from chicken trachea (**A**–**D**) and lung (**E**–**H**). (**A**,**E**) Relative abundance of high mannose, hybrid, and complex-type *N*-glycans, categorized by branching pattern (Supplementary Fig. S3). (**B**,**F**) Relative abundance of *N*-glycans, categorized by core structures. (**C**,**G**) Relative abundance of neutral, sialylated, sulfated, and phosphorylated *N*-glycans in chicken trachea and lung. Sulfated glycan groups include those with simultaneously sialylated glycans, whereas sialylated glycan groups do not include any sulfated glycans. Each value (%) in *A*–*C* and *E*–*G* is expressed relative to the total amount of PA-*N*-glycans (= 100%) in Supplementary Table S1. (**D**,**H**) Relative abundance of characteristic branch sequences on complex or hybrid-type glycans, expressed relative to the total amount of GlcNAc/LacNAc/LacdiNAc-containing branches (= 100%) in all PA-*N*-glycans in Supplementary Table S1. Each group of GlcNAc (excluding bisecting GlcNAc), LacNAc, sialyl LacNAc (sLacNAc), Le^x^, and sLe^x^ includes the corresponding branches located at the non-reducing termini of complex- and hybrid-type *N*-glycans. Each group of sulfated LacNAc (sulfo LacNAc), LacdiNAc (including sulfo LacdiNAc and sulfo fucosyl LacdiNAc), and LacNAc repeats includes both sialylated and non-sialylated branch sequences.

**Figure 7:**
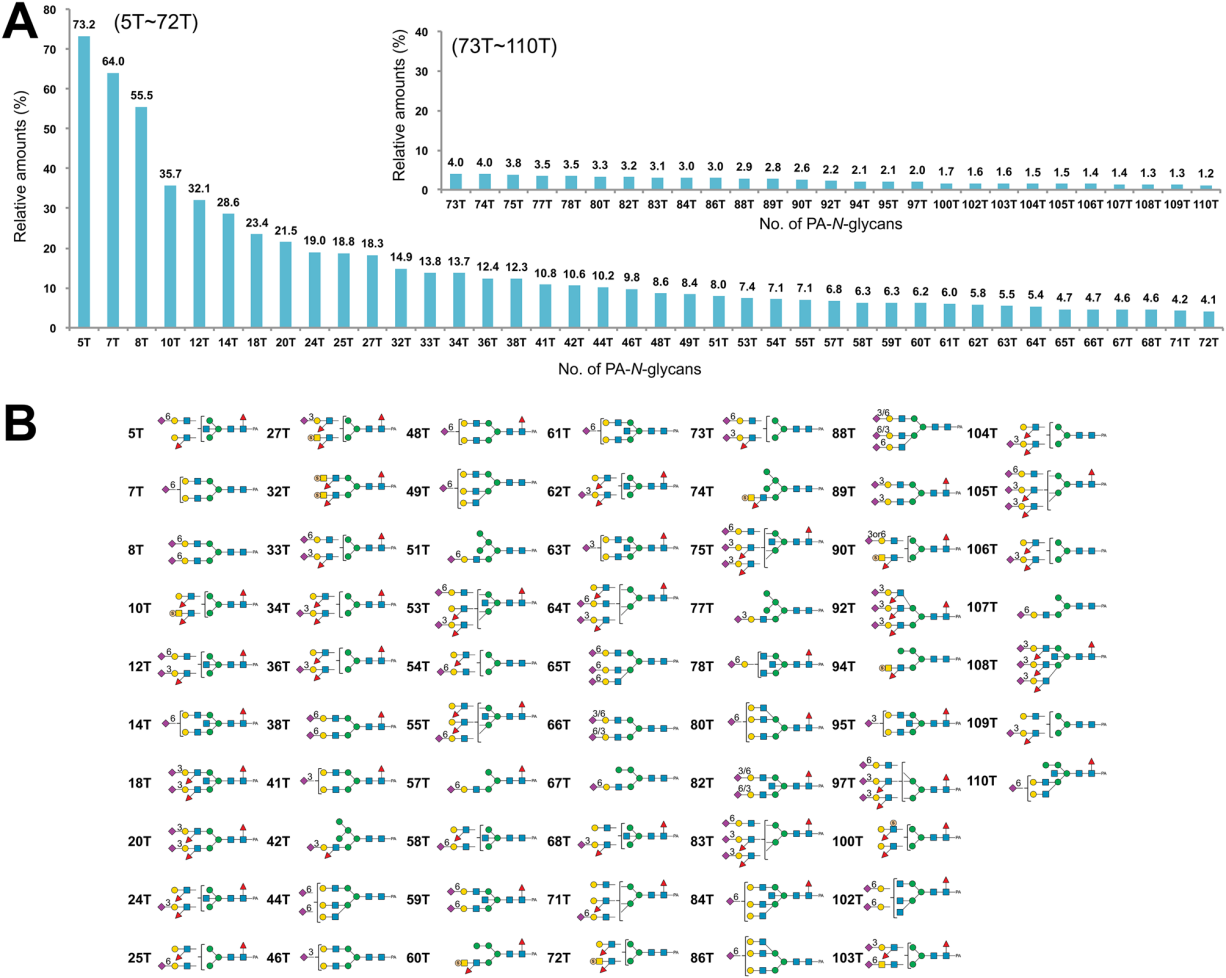

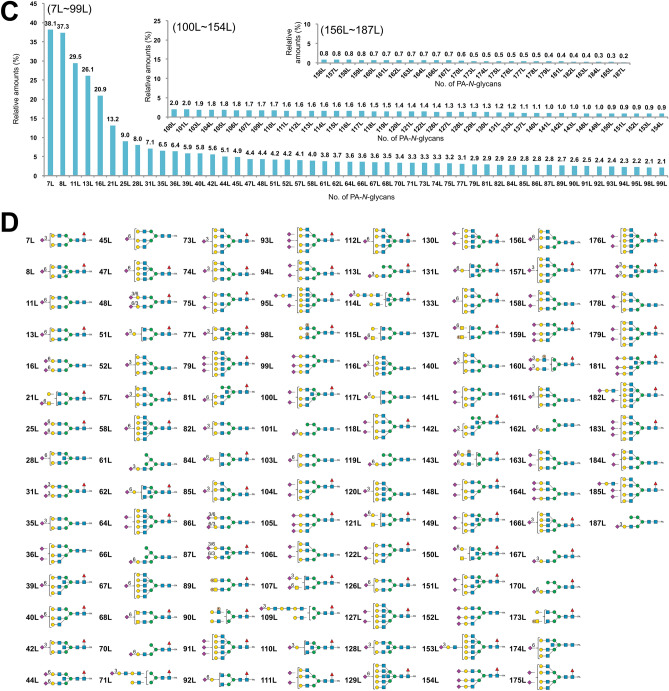
Structures and relative amounts of sialylated and/or sulfated PA-*N*-glycans from chicken trachea and lung. (**A**,**C**) Relative amounts of sialylated and/or sulfated PA-*N*-glycans of trachea (**A**) and lung (**C**). The upper values on each bar of the graph indicate the % of each glycan relative to the most abundant glycan (Man_9_GlcNAc_2_-PA (pk.1-9-1, 1T) in Supplementary Table S1A and S3 for chicken trachea, Gal_2_GlcNAc_2_Fuc_1_C-PA (pk.1-28-1, 1L) in Supplementary Table S1B and S3 for chicken lung), which was assigned a value of 100. The numbers of each PA-*N*-glycan indicate the order when they are arranged in descending order of relative amount in each tissue (1T–111T and 1L–187L in Table S3). (**B**, **D**) Structures of the numbered PA-*N*-glycans in *A* (trachea) and *C* (lung), respectively. Sia-linkages (α2,3, α2,6) that were identified unambiguously are indicated by a number between the Sia and Gal/GalNAc moieties.

Based on the results of LC–MS, MS/MS, exoglycosidase digestions, SALSA, and SALSA/permethylation, we deduced the structures of almost all major PA-*N*-glycans from chicken trachea and lung, including the core structures, branching patterns, and branch sequences. The deduced structures are summarized in Supplementary Table S1, along with the relative amounts calculated from the area of each peak detected by fluorescence and full MS analyses. Table S3 lists of all detected PA*-N*-glycans sorted in descending order of relative amounts. Using the datasets, we calculated the relative amounts of categorized glycan structures (Fig. 6). The ratios of high mannose-, hybrid-, and complex-type glycans were almost the same between trachea and lung, as well as chicken colon^13^. While lung contains certain amounts of tetra- and penta-antennary structures, trachea contains very few tetraantennary structures. Two types of tetraantennary structures were found in lung, with 2,2′,4′,6′-tetra was more abundant than 2,4,2′,4′-tetra, whereas the reverse is true for chicken colon. Approximately half of the *N*-glycans in both tissues possessed core Fuc, but the amounts of *N*-glycans that possess bisecting GlcNAc were slightly higher in trachea (32.2%) than in lung (19.8%). The amounts of sialylated *N*-glycans or sulfated *N*-glycans were higher in trachea (37.0% or 5.5%, respectively) than in lung (32.1% or 0.9%, respectively). Although both trachea and lung contain sulfated *N*-glycans, the former mainly contains sulfo fucosyl LacdiNAc, and the latter contains sulfo LacNAc and sulfo LacdiNAc. Figure 7 shows the sialylated and/or sulfated *N*-glycans in chicken trachea and lung.

To quantify the structural features of branch sequences, the amounts of each GlcNAc/LacNAc/LacdiNAc-containing branch on complex and hybrid-type *N*-glycans were calculated (Fig. 6D,H). While LacNAc or sialyl LacNAc (sLacNAc) sequences were the dominant sequences in lung, they were decreased in trachea as the proportion of Le^x^ or sLe^x^ increased. Small amounts of *N*-glycans possessing LacNAc repeats were detected in lung (1.1%) but not in trachea. LacdiNAc in trachea (4.5%) were mainly sulfo fucosyl LacdiNAc.

## Discussion

One of the main barriers to IAV transmission among species is believed to be the receptor specificity of HAs that bind terminal Sia on host glycans. Different binding preferences among avian IAVs may also be barriers against interspecies transmission among birds. IAVs isolated from ducks rarely infect chickens directly in experiments, although both chicken-origin and duck-origin IAVs bind preferentially to α2,3-Sia. Several groups reported that IAVs from terrestrial poultry including chicken, bind preferentially to 6-sulfo α2,3-sialyl LacNAc, sLe^x^, and/or 6-sulfo sLe^x^, although the binding preferences varied depending on viruses of different subtypes and isolates^6–10^. For example, Gambaryan et al. reported that some chicken IAVs show strong binding to 6-sulfo α2,3-sialyl LacNAc and/or 6-sulfo sLe^x^^6–8^, whereas Hiono et al. reported that a low pathogenic H5N2 isolate from chicken bound preferentially to sLe^x^ rather than to α2,3-sialyl LacNAc^9,10^. The effect of 6-sulfation and/or fucosylation of the GlcNAc moiety of α2,3-sialyl LacNAc on binding to some chicken IAVs was also found in the publically available data of glycan arrays provided by the Consortium for Functional Glycomics (CFG), as shown in the Supplementary Information and Supplementary Table S4. By contrast, IAVs from duck bind preferentially to Siaα2-3Galβ1-3GalNAc/GlcNAc rather than to α2,3-sialyl LacNAc, and fucosylation and/or sulfation of α2,3-sialyl LacNAc results in weaker binding^6–8^. The different glycan specificities of duck and chicken IAVs suggest that the target tissues in these birds may express glycan structures in a species-specific manner.

**Figure 8:**
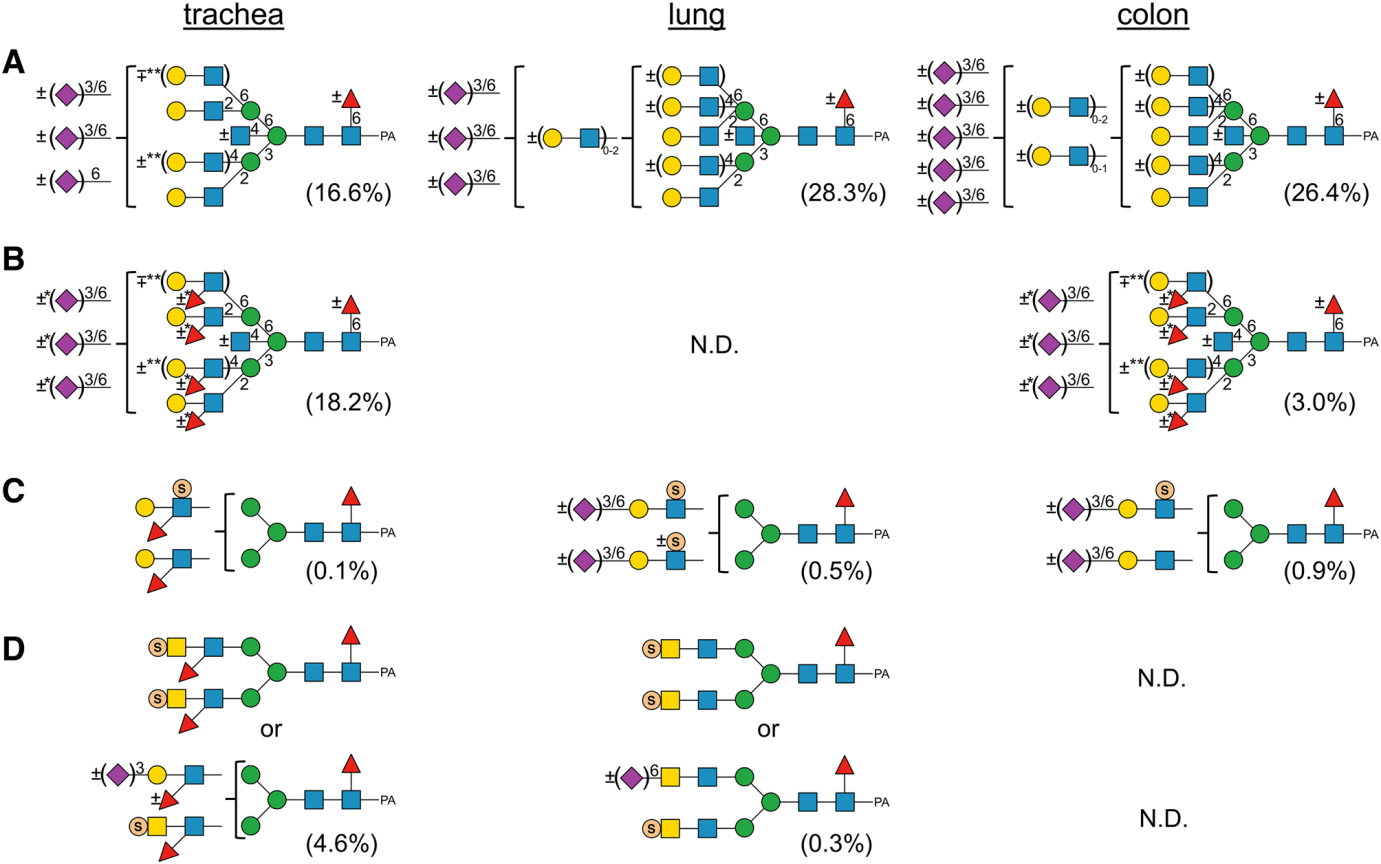
Comparison of the structural features of sialylated and/or sulfated complex-type PA-*N*-glycans from chicken trachea, lung, and colon. (**A**) Summary of the sialylated complex-type PA-*N*-glycans without branch fucosylation in the three tissues. Double asterisks (**) indicate that the 4-branch and the 6′-branch do not exist simultaneously. (**B**) Summary of the sialylated complex-type PA-*N*-glycans with branch fucosylation in chicken trachea and colon. An asterisk (*) indicates that α2,6-sialylations are found only when GlcNAc residues on the same branches are non-fucosylated. (**C**) Summary of the complex-type PA-*N*-glycans with 6-sulfated (sialyl) LacNAc branches with or without branch fucosylation from the three tissues. (**D**) Summary of the complex-type PA-*N*-glycans with sulfated LacdiNAc branches with or without branch fucosylation from trachea and lung. Each value (%) is expressed relative to the total amount of PA-*N*-glycans (= 100%) in Supplementary Table S1.

Although the receptor binding specificity of various HAs of IAVs have been studied extensively, actual glycan structures expressed in avian species have not been extensively studied. Because *N*-glycans rather than *O*-glycans and glycolipids are thought to be the major targets of IAV infection^23^, we analyzed *N*-glycan structures of chicken trachea and lung, in addition to colon^13^. Figure 8 represents the summary of structural features of sialylated or sulfated complex-type *N*-glycans with or without branch fucosylation in chicken trachea, lung, and colon. Our results indicated three major aspects of *N*-glycans in these tissues in terms of sialylated or sulfated branch structures. First, the relative amounts of α2,6-Sia in trachea and lung were slightly higher than those of α2,3-Sia (Fig. 5), whereas chicken colon expressed more α2,3-Sia than α2,6-Sia^13^. Second, most branches with α2,3-Sia in trachea are α1,3-fucosylated and exist as sLe^x^ (Fig. 8A,B). This glycan epitope was rarely found in lung and only small amounts of *N*-glycans with sLe^x^ were found in colon. Third, 6-sulfo α2,3-sialyl LacNAc, but not 6-sulfo sLe^x^, were detected as minor components in chicken lung and colon (Fig. 8C). In trachea, sulfated *N*-glycans mainly exist as sulfo fucosyl LacdiNAc (Figs. 7, 8D, Supplementary Table S1A, S3, Fig. S5-3), and 6-sulfo Le^x^ was detected as a minor component (Fig. 8C, Supplementary Fig. S5-4, S6). No 6-sulfo sLe^x^ was detected in trachea, lung, or colon, although this structure was reported as the common receptor determinant recognized by H5, H6, H7, and H9 influenza viruses of terrestrial poultry^7^. In addition to these three major aspects, multiple branching structures (up to pentaantennary structures) with some LacNAc repeat sequences were identified in chicken lung and colon, but not in trachea (Fig. 8A). The *N*-glycans in the colon appear more complex than those in the lung, due to the presence of multiple sialylations (up to five per glycan) and more extended LacNAc repeats. Currently, no sufficient information is available to evaluate how these complex glycan structures affect the bindings of avian IAVs. They are not covered by the CFG glycan array, even though some of the more complex *N*-glycans are included in newer versions^24,25^.

Abundant expression of sLe^x^ on the surface of chicken trachea epithelial cells was reported previously using specific monoclonal antibodies against this epitope^9^. Our *N*-glycomic data clearly support the results of immunohistochemical staining. Because previous reports indicated that some chicken IAVs, but not duck IAVs, bind preferentially to sLe^x^^7,9,10^, our current *N*-glycomic data suggest that HAs of the chicken IAVs may have adapted to bind sLe^x^. Substitution of two amino acid residues at the receptor binding site of H5 HA was suggested to contribute to increased binding affinity to sLe^x^^10^. Moreover, our results indicate that Siaα2-6Gal and sLe^x^ are often located on the same PA-*N*-glycans at different branches (Figs. 3, 7, Supplementary Table S1A, S3), suggesting that α2,6-sialyltransferases, α2,3-sialyltransferases, and α1,3-fucosyltransferases acting on *N*-glycans are expressed in the same cells, and can act on the same glycosylation sites on glycoproteins. This fact is consistent with the results of histochemical staining with specific lectins and antibodies showing that the surfaces of trachea epithelial cells present both Siaα2-6Gal and sLe^x^^9,26^. Therefore, either Siaα2-6Gal or sLe^x^ could be selected to attach to target cells. Nevertheless, HAs of chicken IAVs studied to date bind preferentially to Siaα2-3Gal, including sLe^x^, but not to Siaα2-6Gal, suggesting that only IAVs with HAs that bind Siaα2-3Gal/sLe^x^ can propagate in chicken trachea. The opposite appears to be the case for human IAVs, which express HAs that bind preferentially to Siaα2-6Gal. In contrast to the results of lectin staining, which suggested the dominance of α2,6-Sia in human respiratory tracts^4^, glycan structural analysis suggested a comparable abundance of α2,3-Sia and α2,6-Sia^27^. However, HAs of some human IAVs bind preferentially to Siaα2-6Gal, unlike HAs of chicken IAVs. Therefore, not only the abundance of Siaα2-6Gal in human respiratory tract, but some other unknown factors in human may influence the alteration of the receptor specificity of IAVs from Siaα2-3Gal to Siaα2-6Gal.

There are many lines and breeds of chickens, both commercial and indigenous. Among them, we used Chunky (a chicken broiler) as a source of material for this study. Although there is a lack of information about variations among chicken lines/breeds in terms of glycan structural modifications, we need to be aware of the possibility that glycan variations affect the susceptibility of different lines/breeds to infection by avian IAVs. For generalization of glycan structural features in chicken species, accumulation of data from many other lines/breeds in addition to our data is necessary. Another concern is possible glycan variations among individual chickens, as occurs for blood type glycans in humans. In this study, we used a mixture of trachea mucosa obtained from several chickens as a source to analyze glycan structures, since the amount of *N*-glycans isolated from a single chicken was not sufficient for detailed structural analysis with our system. Although our qualitative analysis of glycan structures yielded consistent results in the two independent experiments, quantitative differences in glycans among individual chickens remain unknown. Thus, quantitative and comparative analyses of glycans among individuals and lines/breeds of chickens should be conducted in future studies using improved methods with higher sensitivity.

Different receptor specificities among avian IAVs has also been reported in those originally isolated from terrestrial birds other than chicken, as well as those from some wild waterfowl such as gulls^6,7^. However, there is little information about the glycan structures in these birds. Further studies should clarify the glycan structures of other hosts, particularly natural hosts, i.e., wild waterfowl, and other terrestrial poultry species known to be intermediate transmitters of avian IAVs (e.g., quails and turkeys). It will help to explore the relationship between the glycan-binding specificities of HAs of avian IAVs and the glycan structures expressed on host cells of birds. The glycomic analysis of chicken tissues presented herein can form the cornerstone for further studies of avian IAVs based on avian glycomic analysis.

## Materials and methods

### Materials

Tissues from 3-week-old male chickens (Chunky, purchased from a local farmer in the Niigata area) were a kind gift from Dr. Toshie Sugiyama of Niigata University. All reagents used in this study were the same as those reported previously^13^.

### Preparation of PA-N-glycans from tissue samples

For glycan structural analysis with LC–MS and MS/MS, two independent experiments to prepare PA-*N*-glycans from both chicken trachea and lung were performed. Chicken trachea tissues were obtained from three and four chickens per experiment, whereas chicken lung were obtained from one of the chickens used to obtain trachea per experiments. Isolated chicken trachea and lung were washed several times with phosphate-buffered saline, immediately frozen in liquid nitrogen, and kept at − 70°C until use. The mucosa covering the tracheal lumen was physically detached from the cartilage by tweezers and used for preparation of glycans. After tissues (100–200 mg, wet weight) were homogenized with a Polytron homogenizer, *N*-glycans were prepared as described previously^13^. *N*-Glycans released by glycoamidase F (GAF, aka *N*-glycosidase F and PNGase F) treatment were derivatized with PA as described previously^28^. Mixtures of PA-*N*-glycans were separated by HPLC using a TSKgel DEAE-5PW column, as described previously^22,29^. PA-glycans were detected using an FLD with an excitation wavelength of 310 nm and an emission wavelength of 380 nm. Each fraction was analyzed by liquid chromatography-mass spectrometry (LC–MS) and MS/MS using a C18 reversed-phase LC column as described later, and simultaneously monitored with an FLD (Fig. 1).

### Linkage-specific derivatization of sialic acids and permethylation

To determine the linkages of sialic acids on non-reducing termini, portions of sialylated PA-*N*-glycans were derivatized with linkage-specific alkylamidation, as described previously^22^. For permethylation of alkylamidated PA-*N*-glycans, sialylated glycans were alkylamidated and permethylated sequentially, as described previously^20^.

### Exoglycosidase digestion

Sialylated PA-*N*-glycans were digested with neuraminidase (α2-3,6,8,9 neuraminidase from *Arthrobacter ureafaciens*). Neutral or desialylated PA-*N*-glycans were digested with α1-3,4 fucosidase from the sweet almond tree, then with β1-4 galactosidase S from *Streptococcus pneumoniae*. These enzymatic reactions were performed for 16–48 h at 37°C in 50 mM sodium acetate (pH 5.5) containing 5 mM CaCl_2_. After each glycosidase digestion, the products were analyzed by LC–MS and MS/MS as described below.

### Online LC–MS, MS/MS, and MS^n^ analyses of glycans

MS analysis of PA-*N*-glycans was performed by ESI–MS on an LTQ XL linear ion trap mass spectrometer coupled to a Dionex U3000 HPLC system and an ESI-probe (H-ESI-II, Thermo Fisher Scientific, Waltham, MA, USA). MS data were recorded and analyzed using Xcalibur 2.2 software (Thermo Fisher Scientific). All conditions for MS analyses of PA-*N*-glycans, with or without enzymatic or chemical modifications, were as previously described^13^. Some PA-*N*-glycans from glycoproteins (Supplementary Fig. S2) were also analyzed under the same LC–MS and MS/MS conditions as reference standards.

Glycan structures were deduced based on the elution positions on reversed-phase LC, full MS, MS/MS, and MS^n^, as well as known biosynthetic pathways of vertebrate glycans. Symbol Nomenclature for Glycans was used for monosaccharide symbols^30^, except for sulfate and phosphate groups. The relative amount of each PA-*N*-glycan was quantified based on the integration of fluorescence signals after LC separation. When fluorescence intensity peaks included more than two kinds of PA-glycans with different mass values, their proportions were estimated using the ratios of integrated ion intensities for each *m/z* value detected at the corresponding times.

## Supplementary Information

Supplementary Figures.

Supplementary Information.

Supplementary Table S1.

Supplementary Table S2.

Supplementary Table S3.

Supplementary Table S4.

Supplementary Table S5.

Supplementary Table S6.

## Data Availability

The raw LC–MS and MS/MS data for intact PA-*N*-glycans have been deposited to GlycoPOST (the announced ID: GPST000237). The other datasets generated and/or analyzed during the current study are available from the corresponding author on reasonable request.
