# Supplementary material for: Structural analysis of N-glycans in chicken trachea and lung reveals potential receptors of chicken influenza viruses
Source: Sci Rep. 2022 Feb 8;12:2081. doi: 10.1038/s41598-022-05961-x (PMC8827061; doi:10.1038/s41598-022-05961-x)
Supplement: Supplementary file 1 — Supplementary Figures. [file 41598_2022_5961_MOESM1_ESM.pdf]

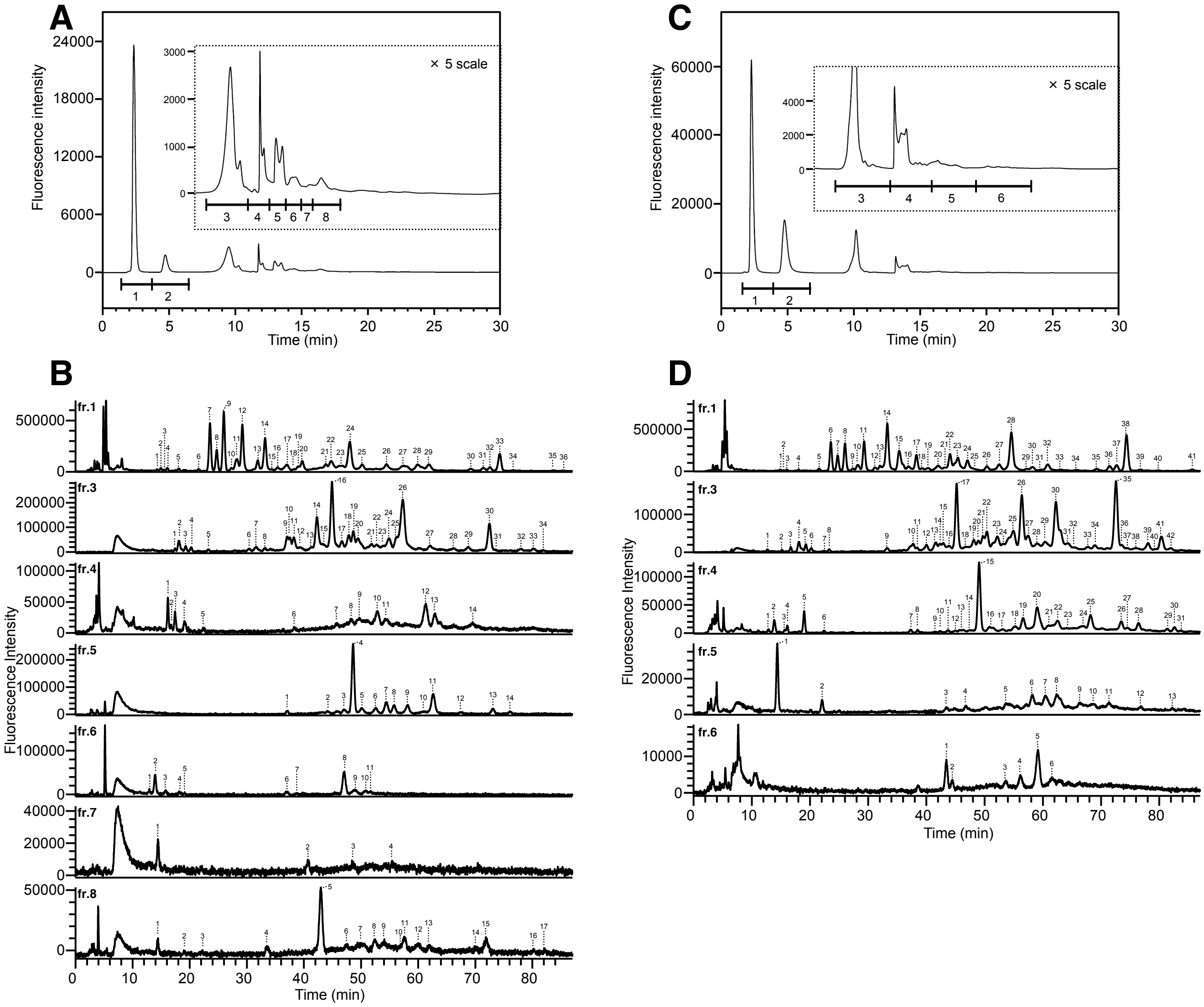

Figure S1. Elution profiles of PA-*N*-glycans from chicken trachea (A, B) and lung (C, D). (A, C) PA-*N*-glycans from chicken trachea (A) and lung (C) were separated with a DEAE column. Each numbered major peak was fractionated and analyzed by LC-MS and MS/MS. (B, D) Elution profiles of PA-*N*-glycans from chicken trachea (B) and lung (D) on reversed-phase LC. Each fraction from the DEAE column was analyzed. The profile of fr. 2 from the DEAE column is not shown, because no obvious signals of PA-glycans were detected. Most of the detectable peaks eluted in 10–87 min were numbered, regardless of whether they contained PA-*N*-glycans. The results of LC-MS and MS/MS are summarized in Supplementary Table S1.

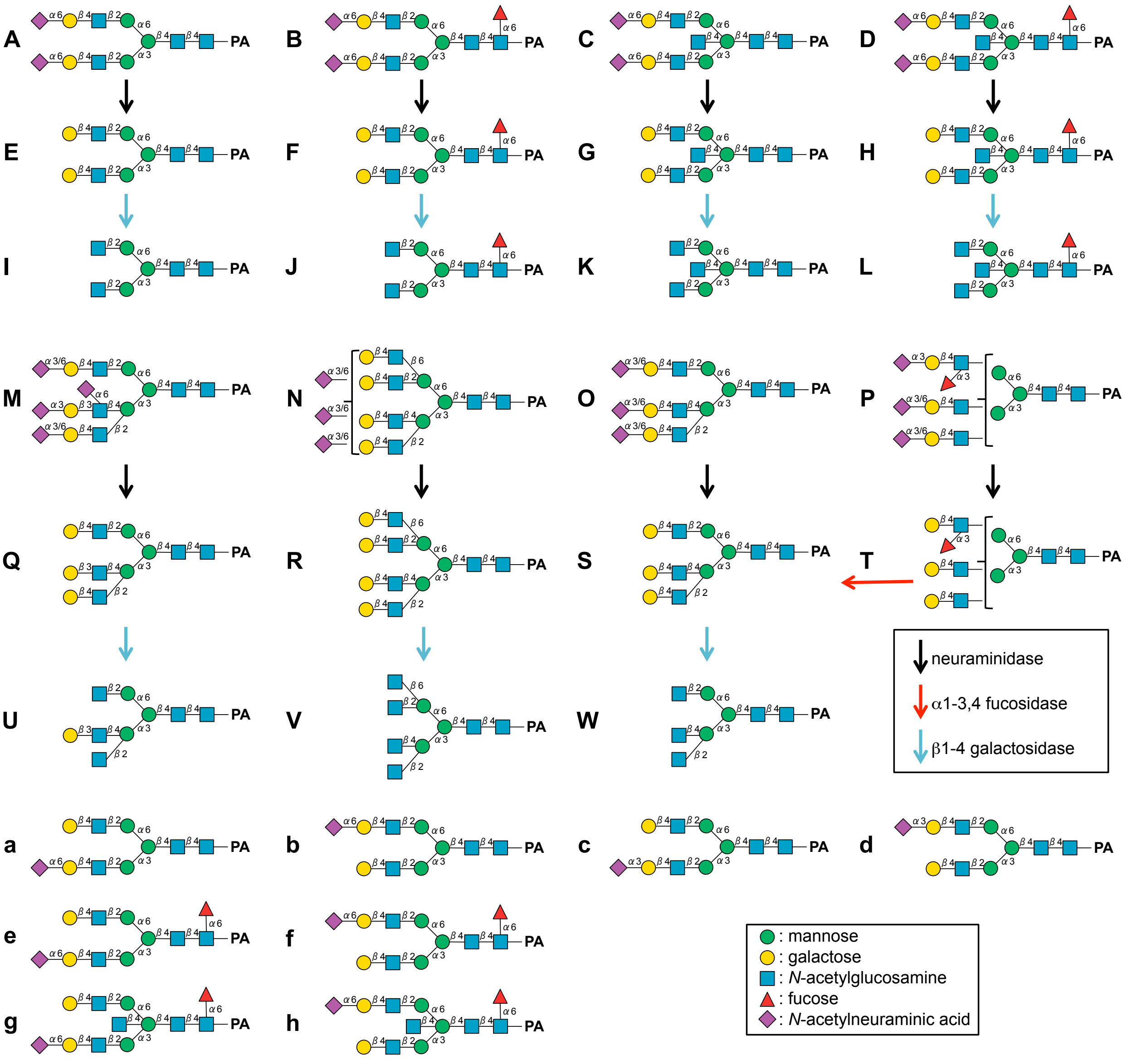

Figure S2. Structures of standard PA-*N*-glycans used for reversed-phase LC-MS and MS/MS. Sialylated PA-*N*-glycans were prepared from human γ-globulin (A, B, C, D), bovine fetuin (M), and α1-AGP (N, O, P), and sequentially digested with neuraminidase, α1-3,4 fucosidase (for T), and β1-4 galactosidase. α2,6-Monosialylated PA-*N*-glycans were prepared from human transferrin (a, b) and human γ-globulin (e, f, g, h). α2,3-Monosialylated PA-*N*-glycans (c, d) were prepared by treatment of asialo-biantennary PA-*N*-glycans with recombinant α2,3-sialyltransferase from *Photobacterium phosphoreum*[22]. The standard Symbol Nomenclature for Glycan system was used for monosaccharide symbols[30]. Reproduced from the previous publication[13].

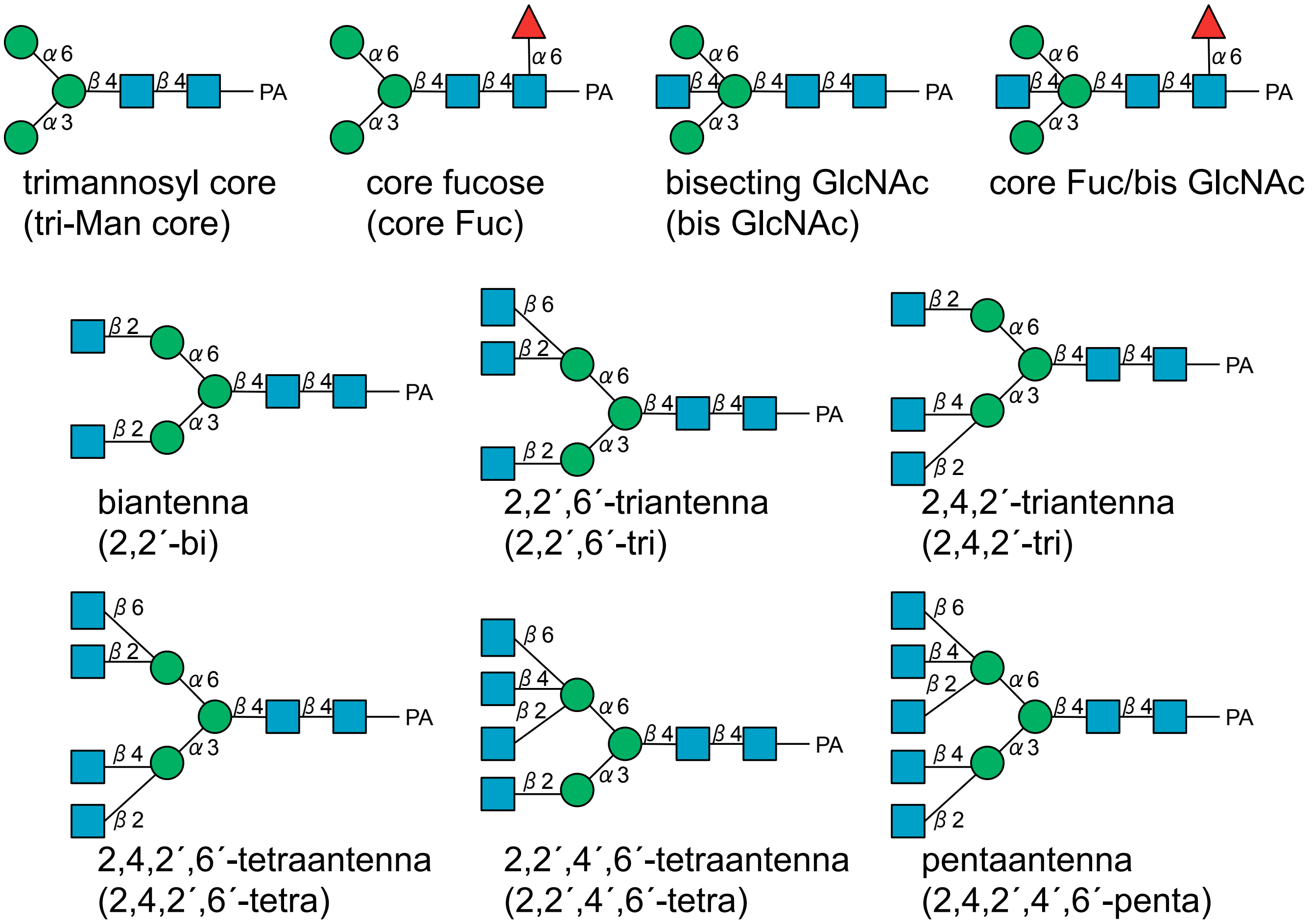

Figure S3. Core and branching structures of PA-*N*-glycans used in this study. The represented branching pattern in this figure is also used in other figures and tables. Reproduced from the previous publication[13].

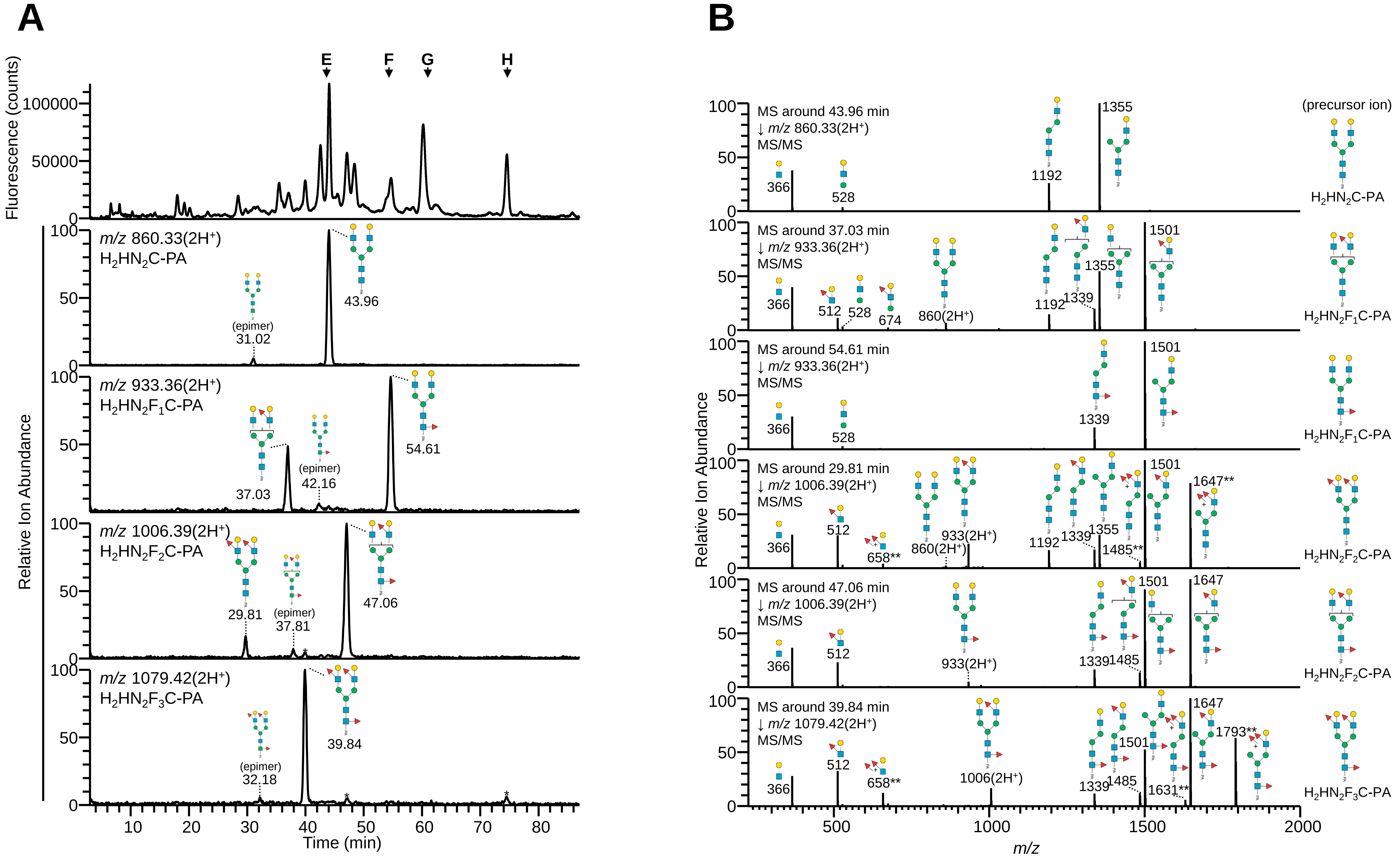

Figure S4-1. Different elution positions among glycan isomers with or without multiple fucosylation on reversed-phase LC. (A) EICs at *m/z* 860.33, 933.36, 1006.39, and 1079.42 of PA-*N*-glycans in neuraminidase-treated fr. 3 of chicken trachea. Arrows with alphabetical characters indicate the elution positions of the standard PA-*N*-glycans (Supplementary Figure S2). The peaks indicated by an asterisk (*) are probably artifactual ion signals derived from large amounts of PA-*N*-glycans eluted around the corresponding times. (B) Comparison of MS/MS spectra of glycan isomers at *m/z* 860.33, 933.36, 1006.39, and 1079.42 eluted at different times, as shown in Supplementary Figure S4-1A. The structures of fragments shown in the figures are representative examples, and other isomeric ions can also be generated. The peaks indicated by double asterisks (**) are probably artifactual ion signals generated by ion rearrangements.

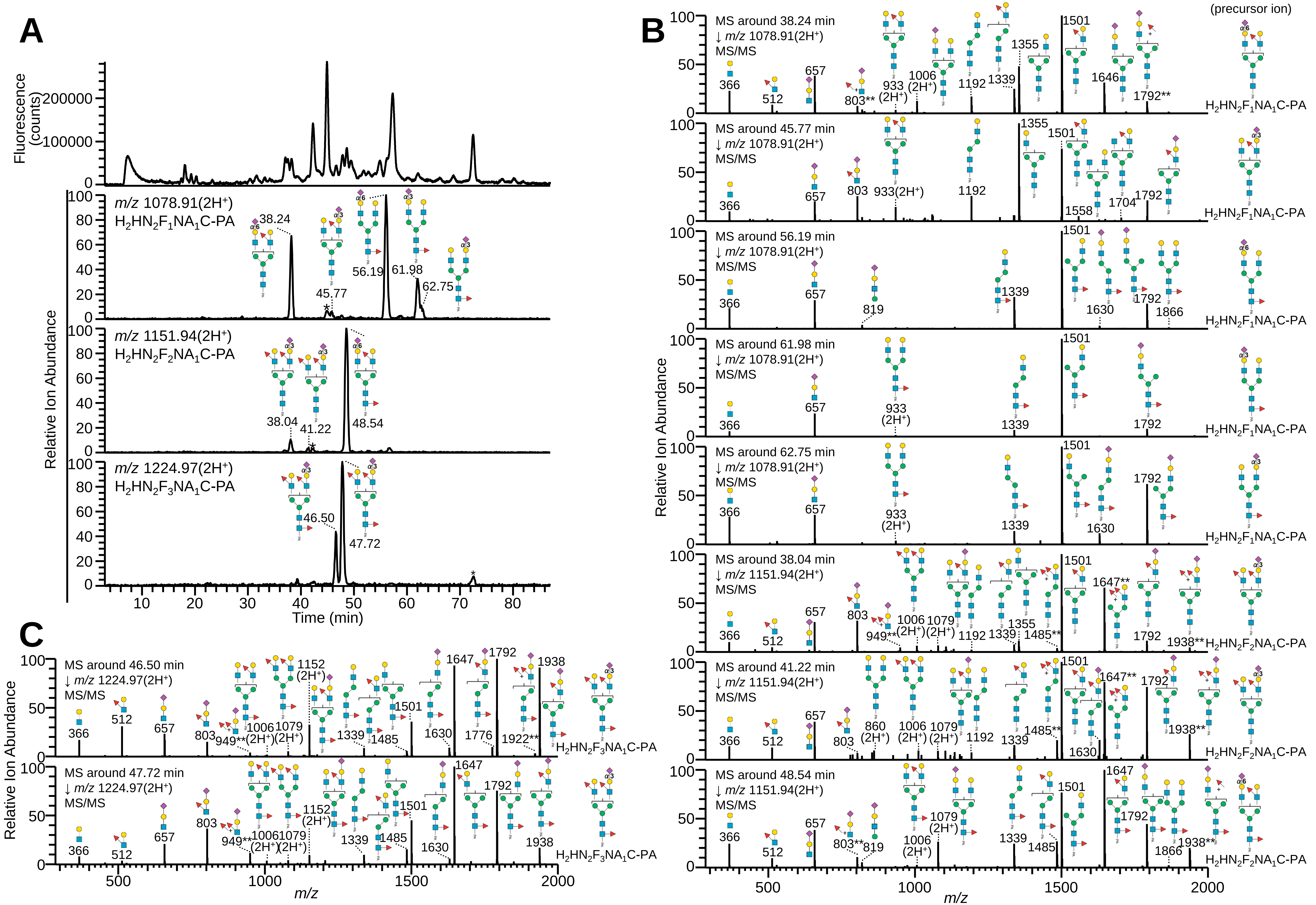

Figure S4-2. Different elution positions among monosialylated glycan isomers with or without multiple fucosylation on reversed-phase LC. (A) EICs at *m/z* 1078.91, 1151.94, and 1224.97 of PA-*N*-glycans in fr. 3 of chicken trachea. The peaks indicated by an asterisk (*) are probably artifactual ion signals derived from large amounts of PA-*N*-glycans eluted around the corresponding times. (B, C) Comparison of MS/MS spectra of glycan isomers at *m/z* 1078.91, 1151.94, and 1224.97 eluted at different times, as shown in Supplementary Figure S4-2A. The structures of fragments shown in the figures are representative examples, and other isomeric ions can also be generated. The peaks indicated by double asterisks (**) are probably artifactual ion signals generated by ion rearrangements.

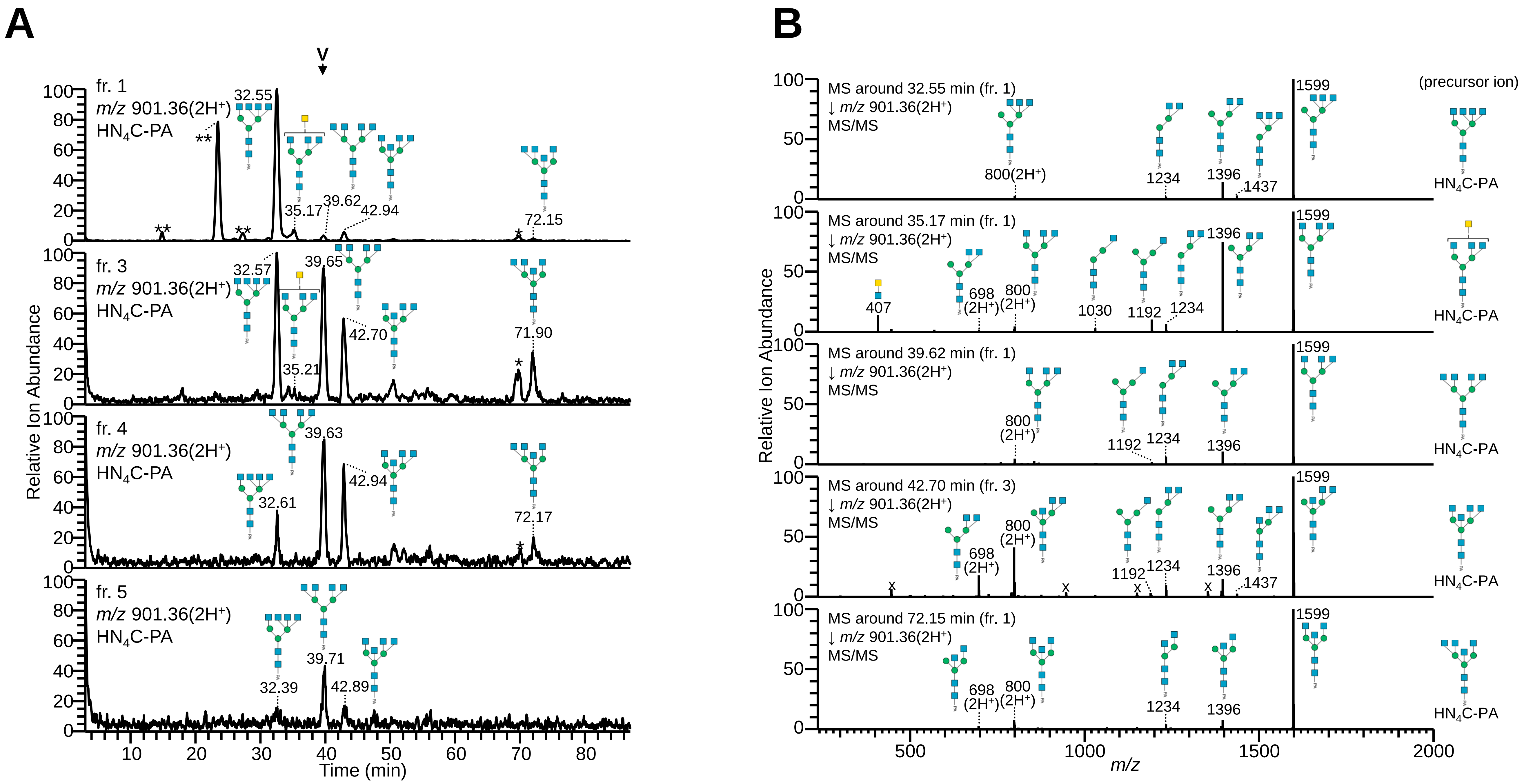

Figure S4-3. Different elution positions among glycan isomers on reversed-phase LC. (A) EICs at *m/z* 901.36 of PA-*N*-glycans in neuraminidase/α1-3,4 fucosidase/β1-4 galactosidase-treated fr. 1, 3, 4, and 5 of chicken lung. An arrow indicates the elution positions of the standard PA-*N*-glycan V (Supplementary Figure S2). The peaks indicated by an asterisk (*) are probably artifactual ion signals derived from large amounts of PA-*N*-glycans eluted around the corresponding times. The peaks indicated by double asterisks (**) are signals derived from PA-*N*-glycans at *m/z* 900.33 [$Hex_8HexNAc_2$-PA(2H$^+$)] eluted around the corresponding times. (B) Comparison of MS/MS spectra of glycan isomers at *m/z* 901.36 of PA-*N*-glycans (fr. 1 or fr. 3) eluted at different times, as shown in Supplementary Figure S4-3A. The structures of fragments shown in the figures are representative examples, and other isomeric ions can also be generated. The peaks indicated an X are probably derived from unrelated precursor ions with a similar *m/z* value.

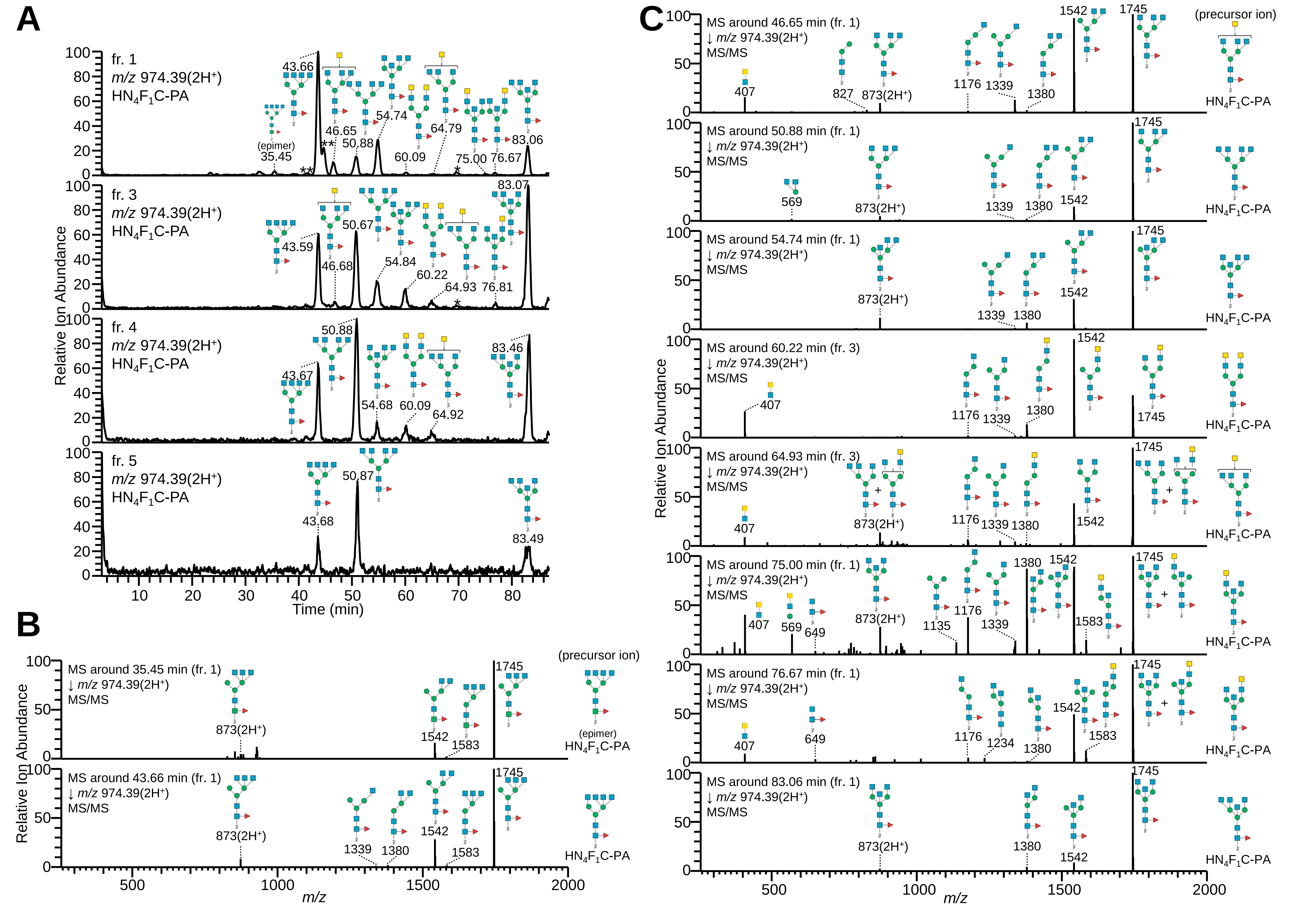

Figure S4-4. Different elution positions among glycan isomers on reversed-phase LC. (A) EICs at *m/z* 974.39 of PA-*N*-glycans in neuraminidase/α1-3,4 fucosidase/β1-4 galactosidase-treated fr. 1, 3, 4, and 5 of chicken lung. The peaks indicated by an asterisk (*) are probably artifactual ion signals derived from large amounts of PA-*N*-glycans eluted around the corresponding times. The peaks indicated by double asterisks (**) are signals derived from PA-*N*-glycans at *m/z* 973.40 [$Hex_2HexNAc_2Fuc_1$-PA(H^+)] eluted around the corresponding times. (B, C) Comparison of MS/MS spectra of glycan isomers at *m/z* 974.39 of PA-*N*-glycans (fr. 1 or fr. 3) eluted at different times, as shown in Supplementary Figure S4-4A. The structures of fragments shown in the figures are representative examples, and other isomeric ions can also be generated.

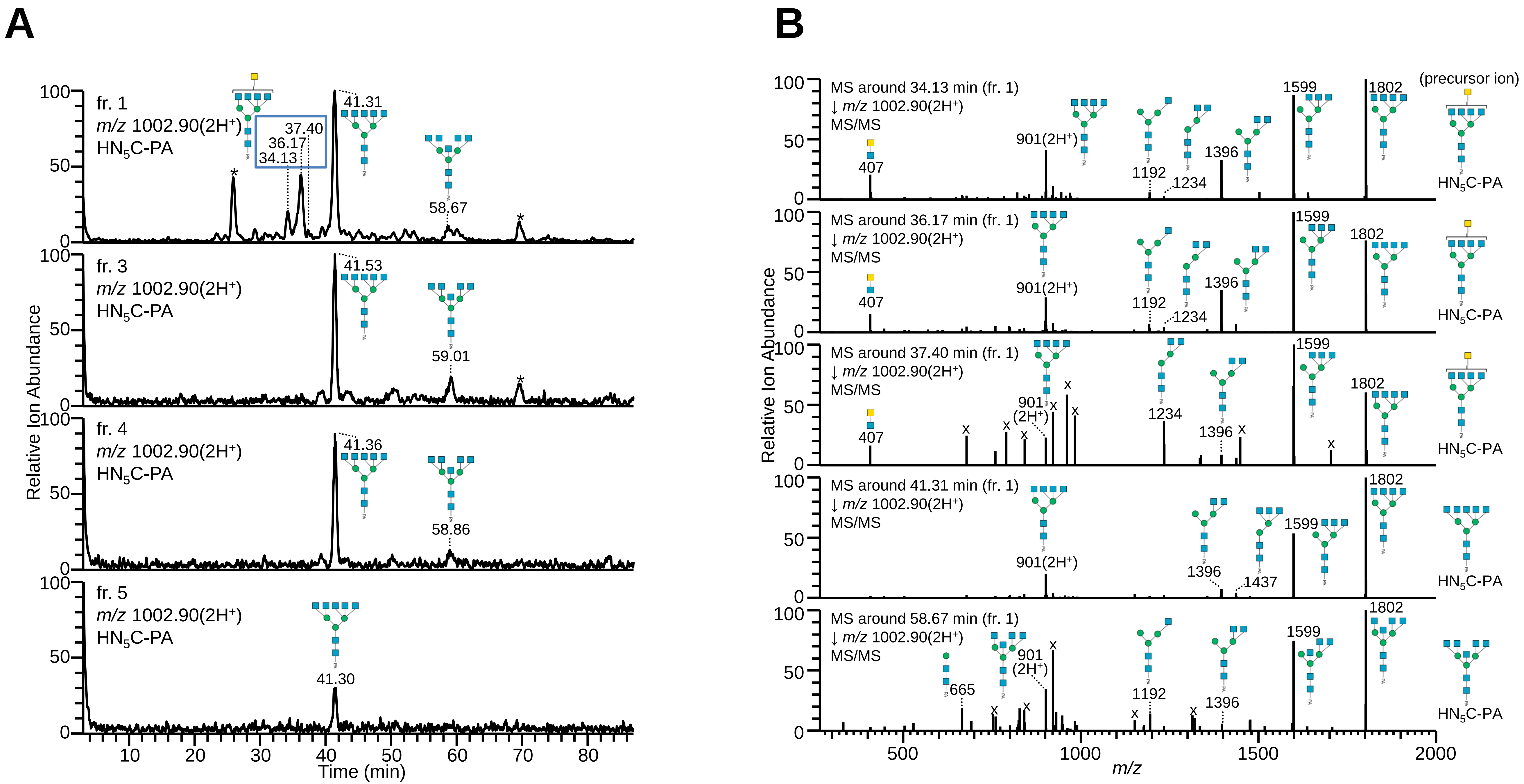

Figure S4-5. Different elution positions among glycan isomers on reversed-phase LC. (A) EICs at *m/z* 1002.90 of PA-*N*-glycans in neuraminidase/α1-3,4 fucosidase/β1-4 galactosidase-treated fr. 1, 3, 4, and 5 of chicken lung. The peaks indicated by an asterisk (*) are probably artifactual ion signals derived from large amounts of PA-*N*-glycans eluted around the corresponding times. (B) Comparison of MS/MS spectra of glycan isomers at *m/z* 1002.90 of PA-*N*-glycans (fr. 1) eluted at different times, as shown in Supplementary Figure S4-5A. The structures of fragments shown in the figures are representative examples, and other isomeric ions can also be generated. The peaks indicated by an X are probably derived from unrelated precursor ions with a similar *m/z* value.

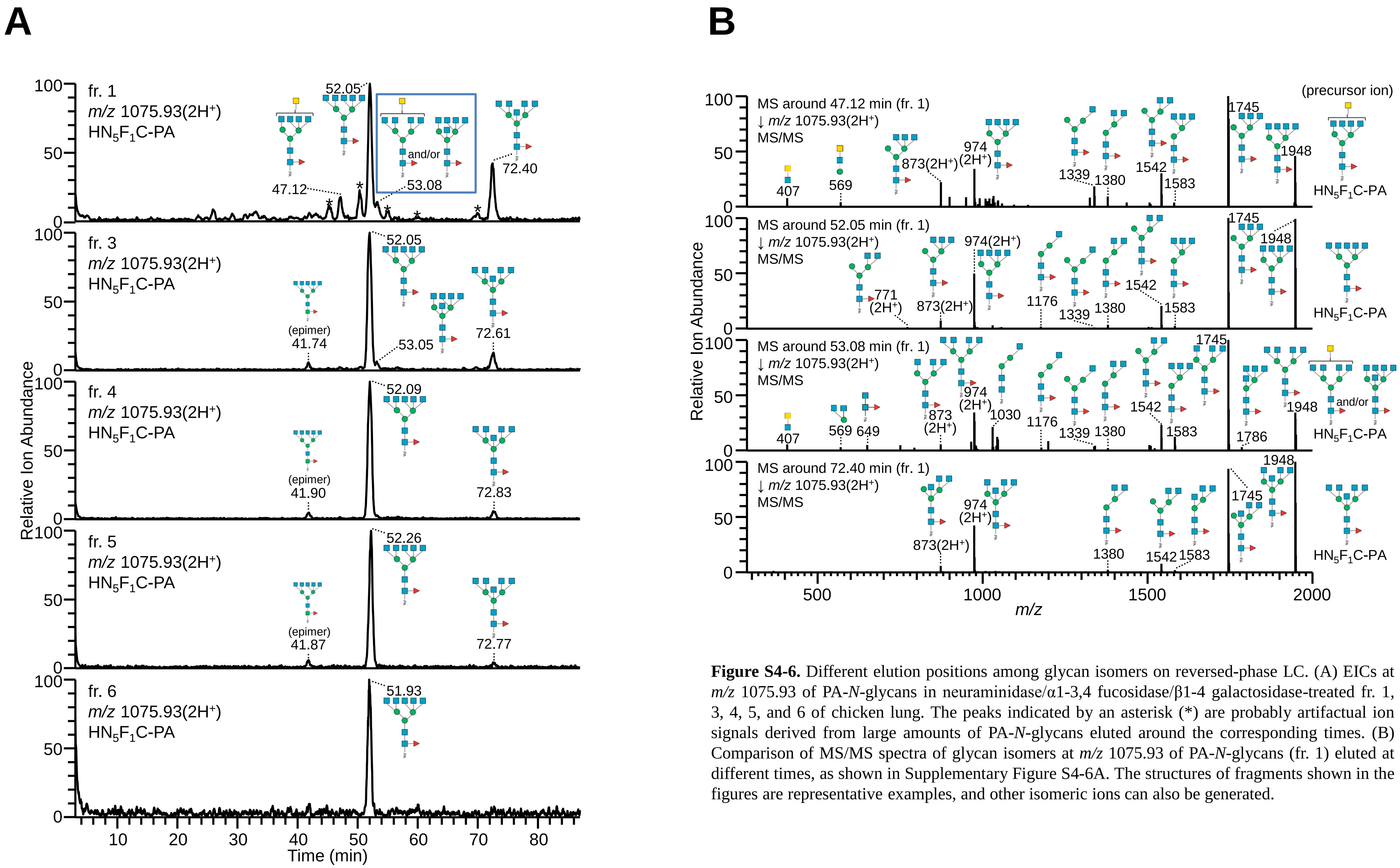

Figure S4-6. Different elution positions among glycan isomers on reversed-phase LC. (A) EICs at *m/z* 1075.93 of PA-*N*-glycans in neuraminidase/α1-3,4 fucosidase/β1-4 galactosidase-treated fr. 1, 3, 4, 5, and 6 of chicken lung. The peaks indicated by an asterisk (*) are probably artifactual ion signals derived from large amounts of PA-*N*-glycans eluted around the corresponding times. (B) Comparison of MS/MS spectra of glycan isomers at *m/z* 1075.93 of PA-*N*-glycans (fr. 1) eluted at different times, as shown in Supplementary Figure S4-6A. The structures of fragments shown in the figures are representative examples, and other isomeric ions can also be generated.

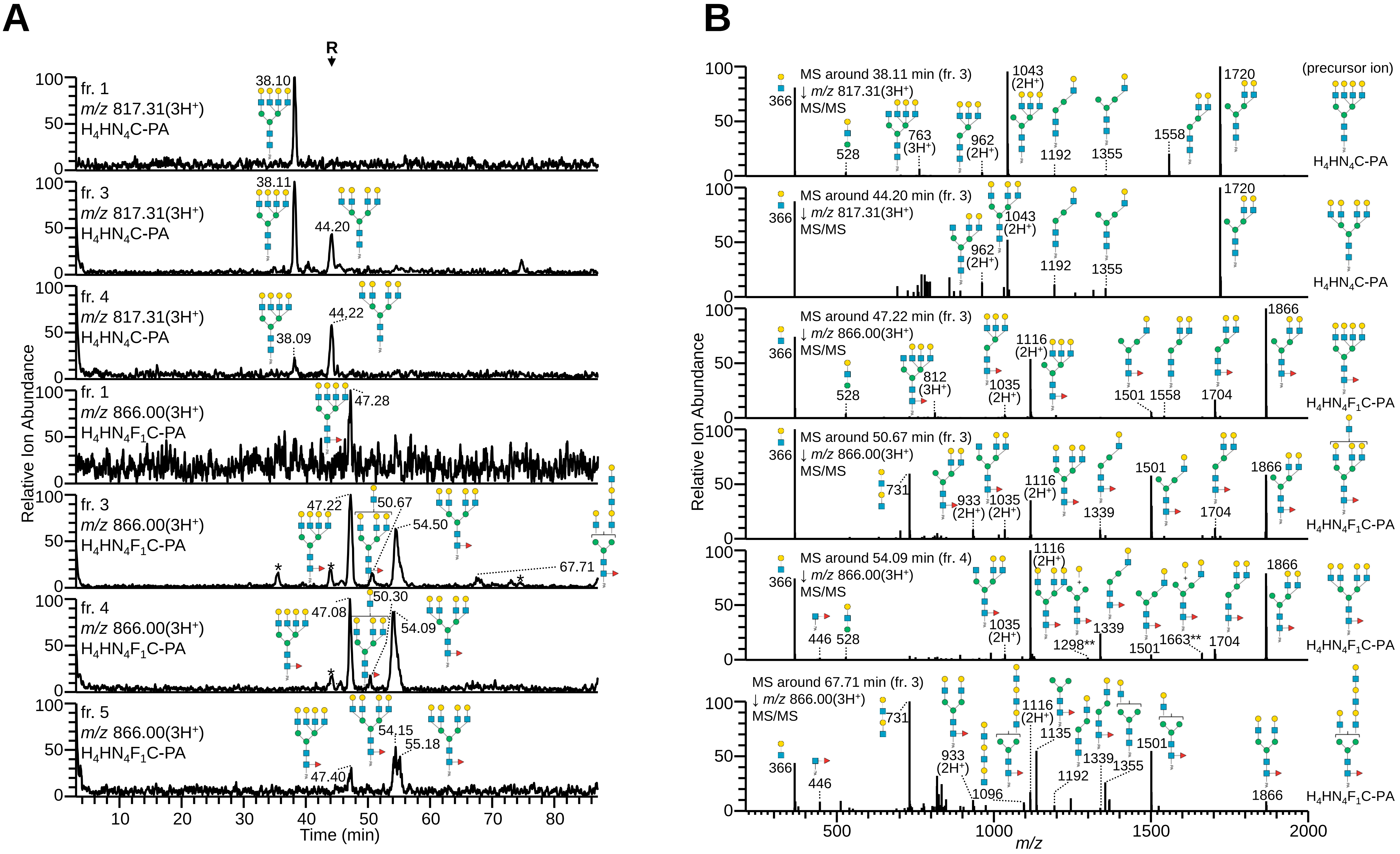

Figure S4-7. Different elution positions among glycan isomers on reversed-phase LC. (A) EICs at *m/z* 817.31 and 866.00 of PA-*N*-glycans in neuraminidase/α1-3,4 fucosidase-treated fr. 1, 3, 4, and 5. An arrow indicates the elution positions of the standard PA-*N*-glycan R (Supplementary Figure S2). The peaks indicated by an asterisk (*) are probably artifactual ion signals derived from large amounts of PA-*N*-glycans eluted around the corresponding times. (B) Comparison of MS/MS spectra of glycan isomers at *m/z* 817.31 and 866.00 ($3H^+$) of PA-*N*-glycans (fr. 3 or fr. 4) eluted at different times, as shown in Supplementary Figure S4-7A. The structures of fragments shown in the figures are representative examples, and other isomeric ions can also be generated. The peaks indicated by double asterisks (**) are probably artifactual ion signals generated by ion rearrangements.

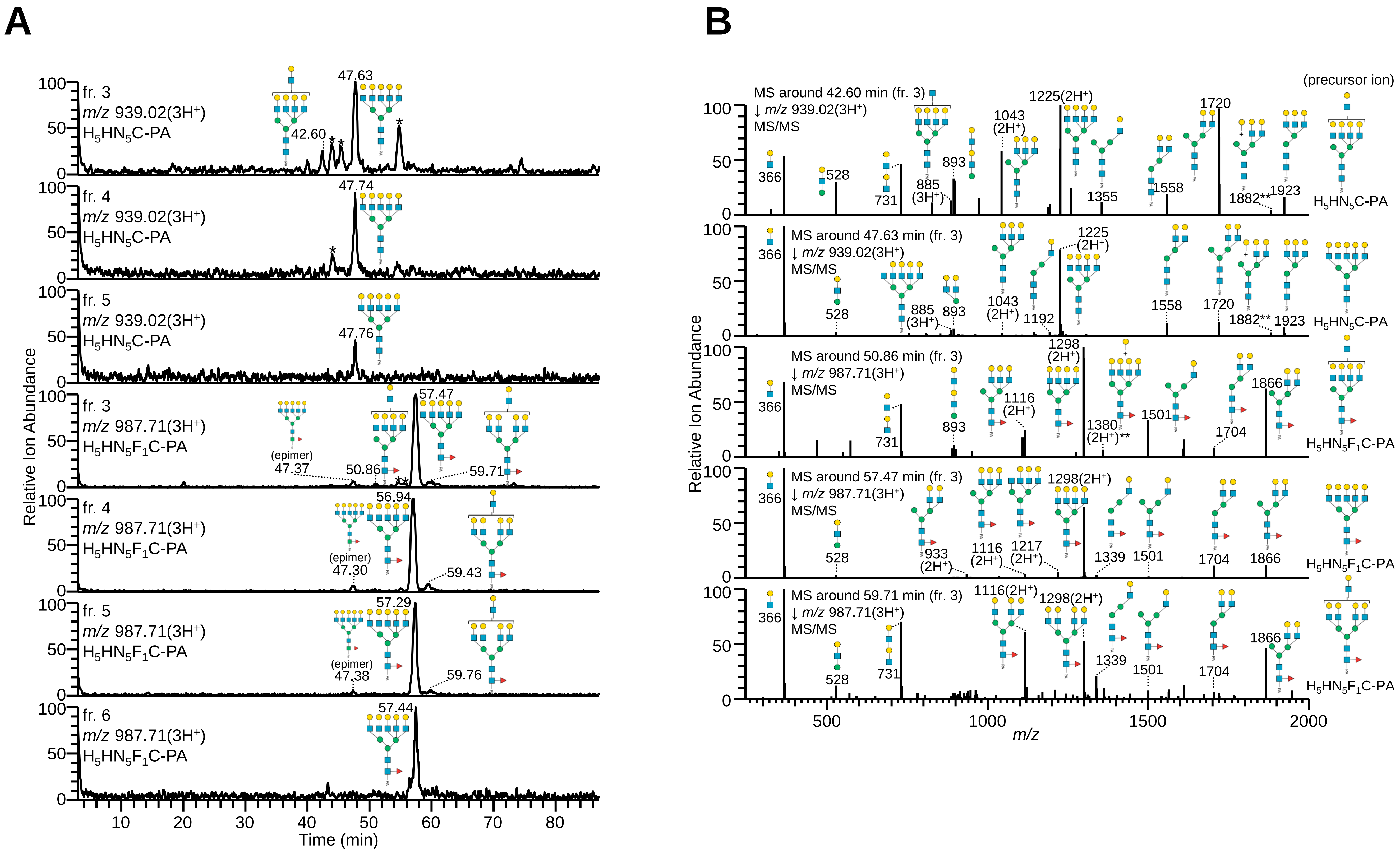

Figure S4-8. Different elution positions among glycan isomers on reversed-phase LC. (A) EICs at *m/z* 939.02 and 987.71 of PA-*N*-glycans in neuraminidase/α1-3,4 fucosidase-treated fr. 3, 4, 5, and 6. The peaks indicated by an asterisk (*) are probably artifactual ion signals derived from large amounts of PA-*N*-glycans eluted around the corresponding times. (B) Comparison of MS/MS spectra of glycan isomers at *m/z* 939.02 and 987.71 ($3H^+$) of PA-*N*-glycans (fr. 3) eluted at different times, as shown in Supplementary Figure S4-8A. The structures of fragments shown in the figures are representative examples, and other isomeric ions can also be generated. The peaks indicated by double asterisks (**) are probably artifactual ion signals generated by ion rearrangements.

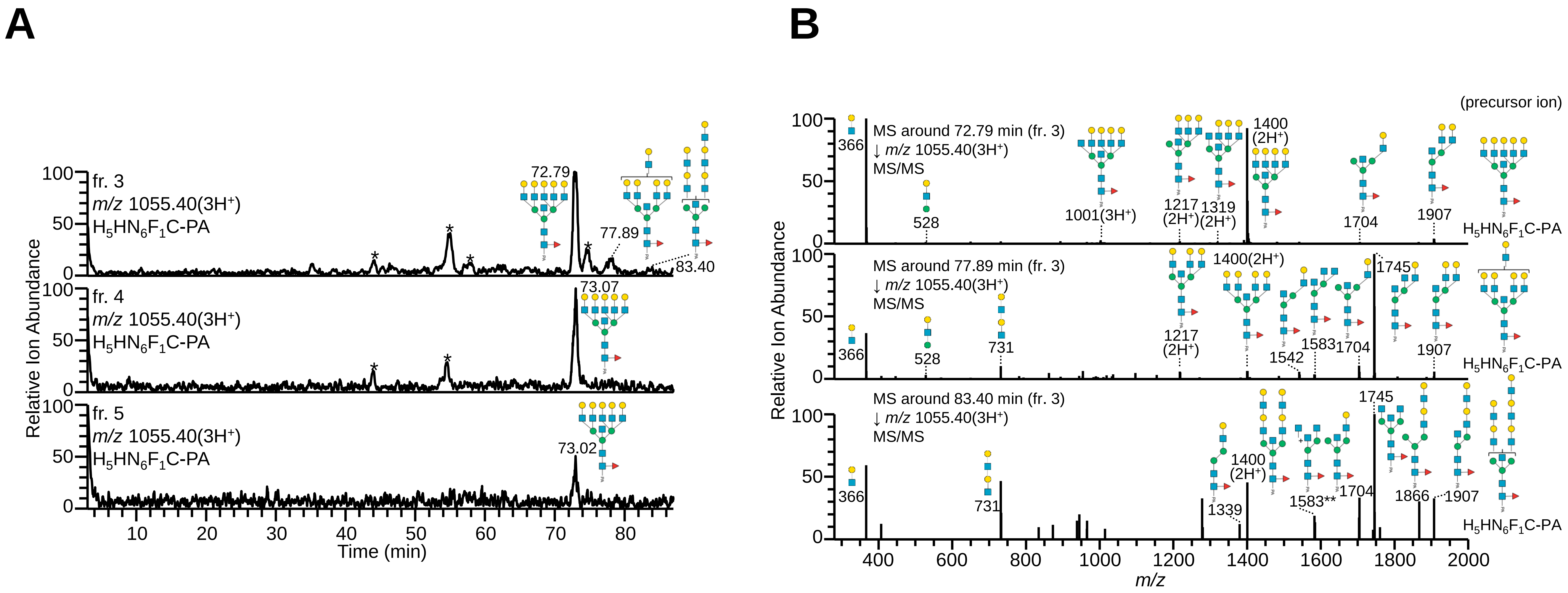

Figure S4-9. Different elution positions among glycan isomers on reversed-phase LC. (A) EICs at *m/z* 1055.40 of PA-*N*-glycans in neuraminidase/α1-3,4 fucosidase-treated fr. 3, 4, and 5. The peaks indicated by an asterisk (*) are probably artifactual ion signals derived from large amounts of PA-*N*-glycans eluted around the corresponding times. (B) Comparison of MS/MS spectra of glycan isomers at *m/z* 1055.40 ($3H^+$) of PA-*N*-glycans (fr. 3) eluted at different times, as shown in Supplementary Figure S4-9A. The structures of fragments shown in the figures are representative examples, and other isomeric ions can also be generated. The peaks indicated by double asterisks (**) are probably artifactual ion signals generated by ion rearrangements.

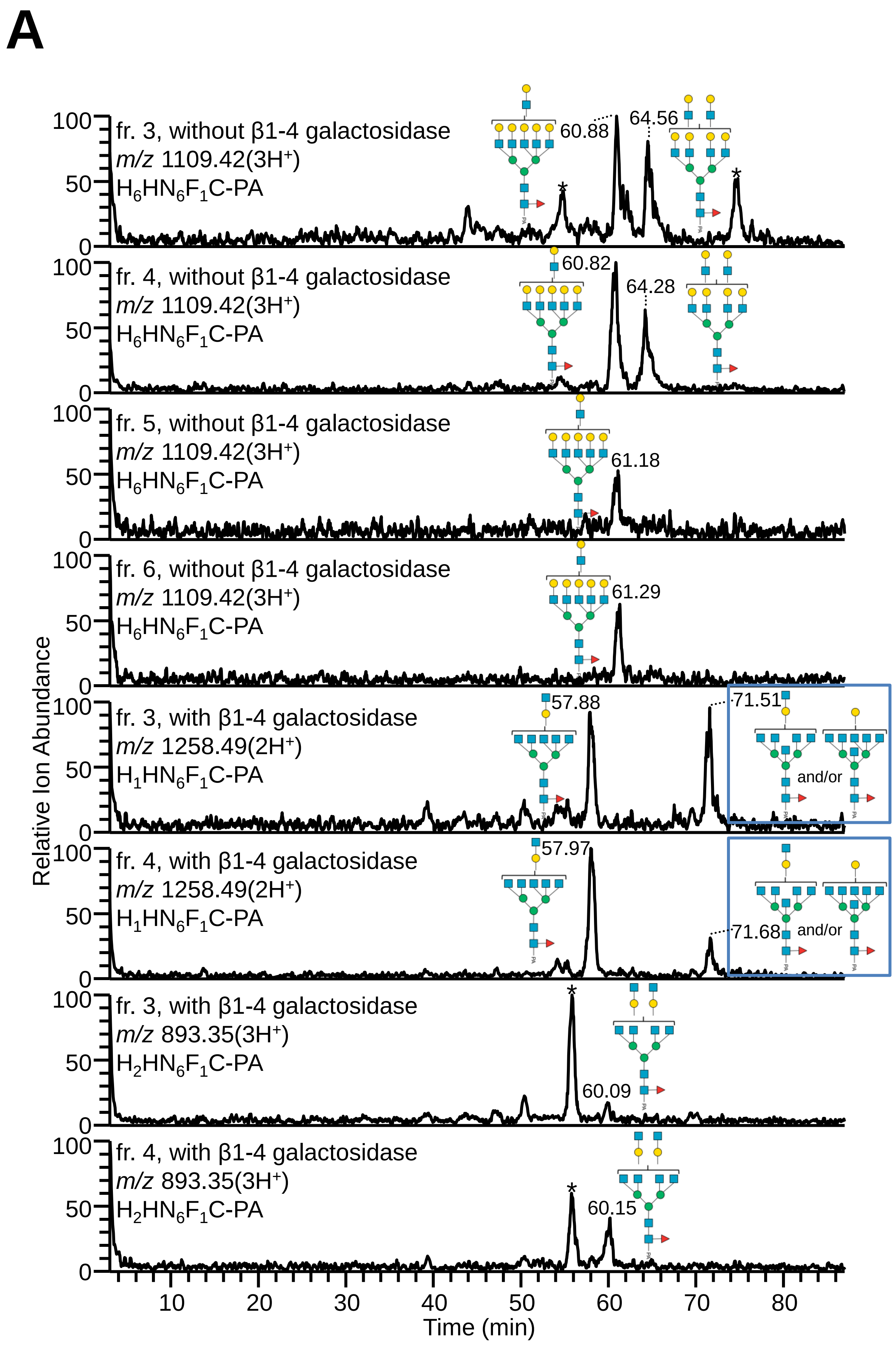

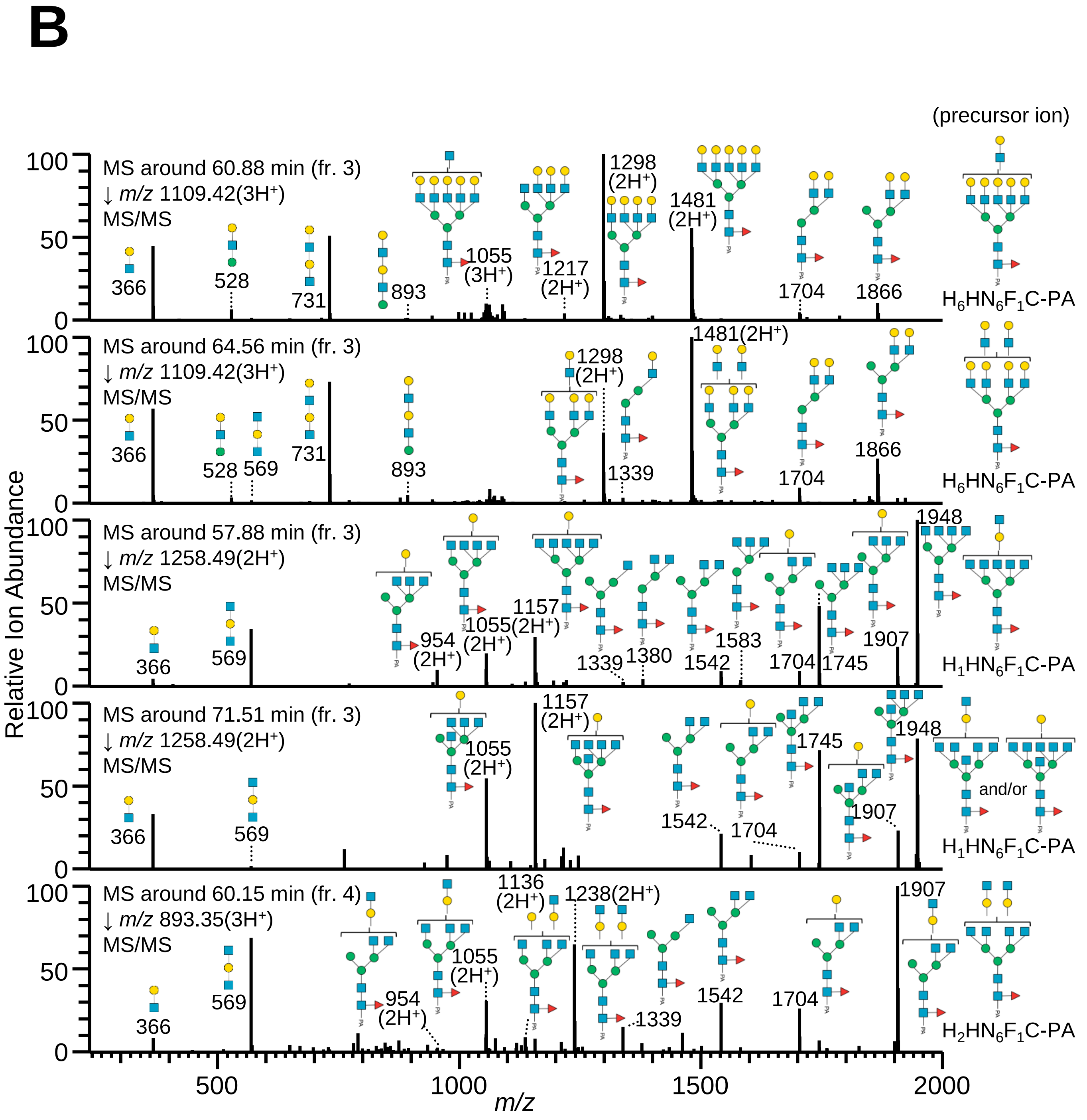

Figure S4-10. Different elution positions among glycan isomers on reversed-phase LC. (A) EICs at *m/z* 1109.42 of PA-*N*-glycans in neuraminidase/α1-3,4 fucosidase-treated fr. 3, 4, 5, and 6, and EICs at *m/z* 1258.49, and 893.35 of PA-*N*-glycans in neuraminidase/α1-3,4 fucosidase/β1-4 galactosidase-treated fr. 3 and 4. The peaks indicated by an asterisk (*) are probably artifactual ion signals derived from large amounts of PA-*N*-glycans eluted around the corresponding times. (B) Comparison of MS/MS spectra of glycan isomers at *m/z* 1109.42, 1258.49, and 893.35 of PA-*N*-glycans (fr. 3 or 4) eluted at different times, as shown in Supplementary Figure S4-10A. The structures of fragments shown in the figures are representative examples, and other isomeric ions can also be generated.

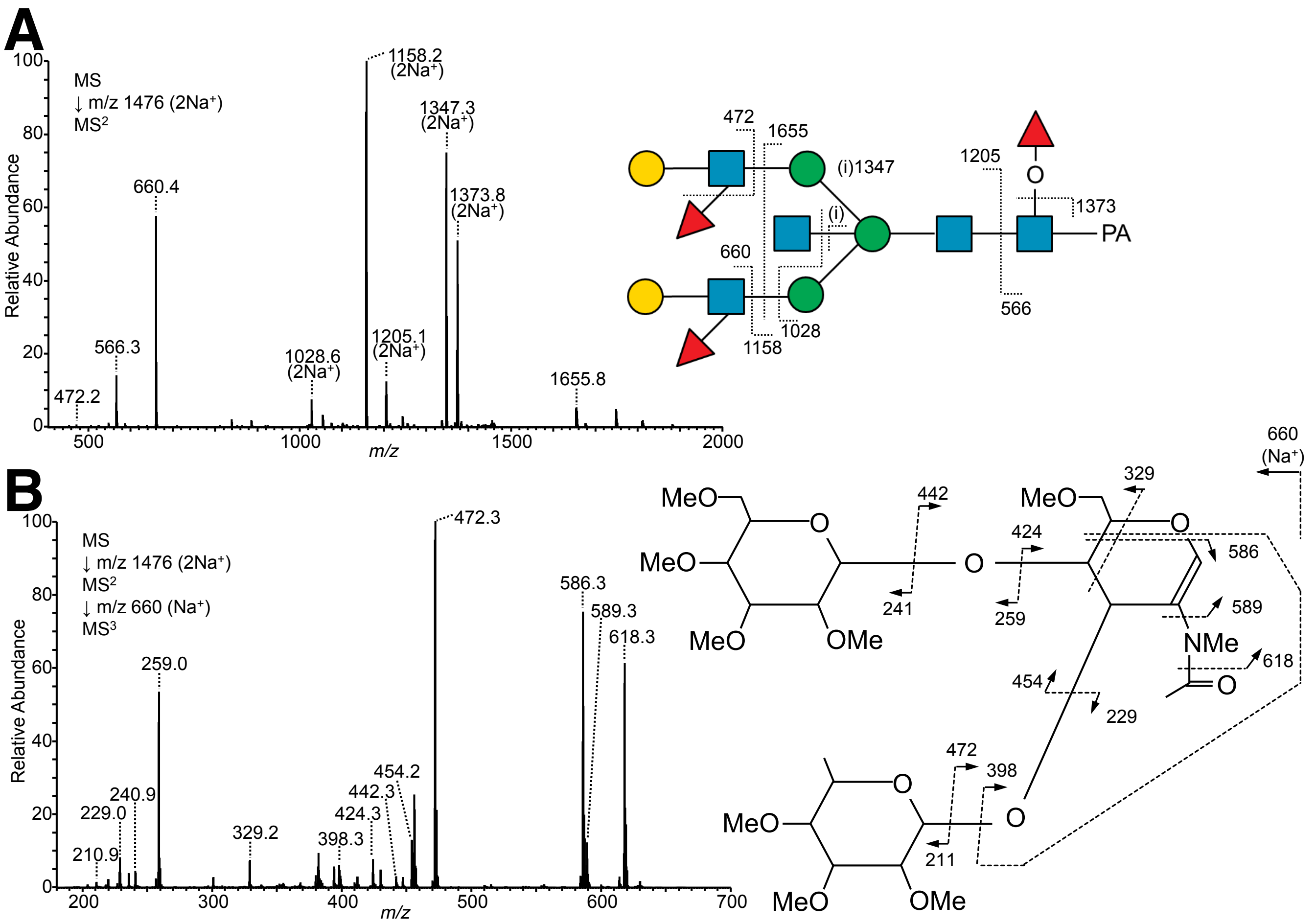

Figure S5-1. MS^n analysis of permethylated PA-*N*-glycans from chicken trachea. (A) MS^2 spectrum of PA-*N*-glycan with Le^x from the doubly sodiated precursor ion at *m/z* 1476, corresponding to $Hex_2HexNAc_3Fuc_3C$-PA. (B) MS^3 spectrum of the B ion fragments $Hex_1HexNAc_1Fuc_1$ at *m/z* 660.

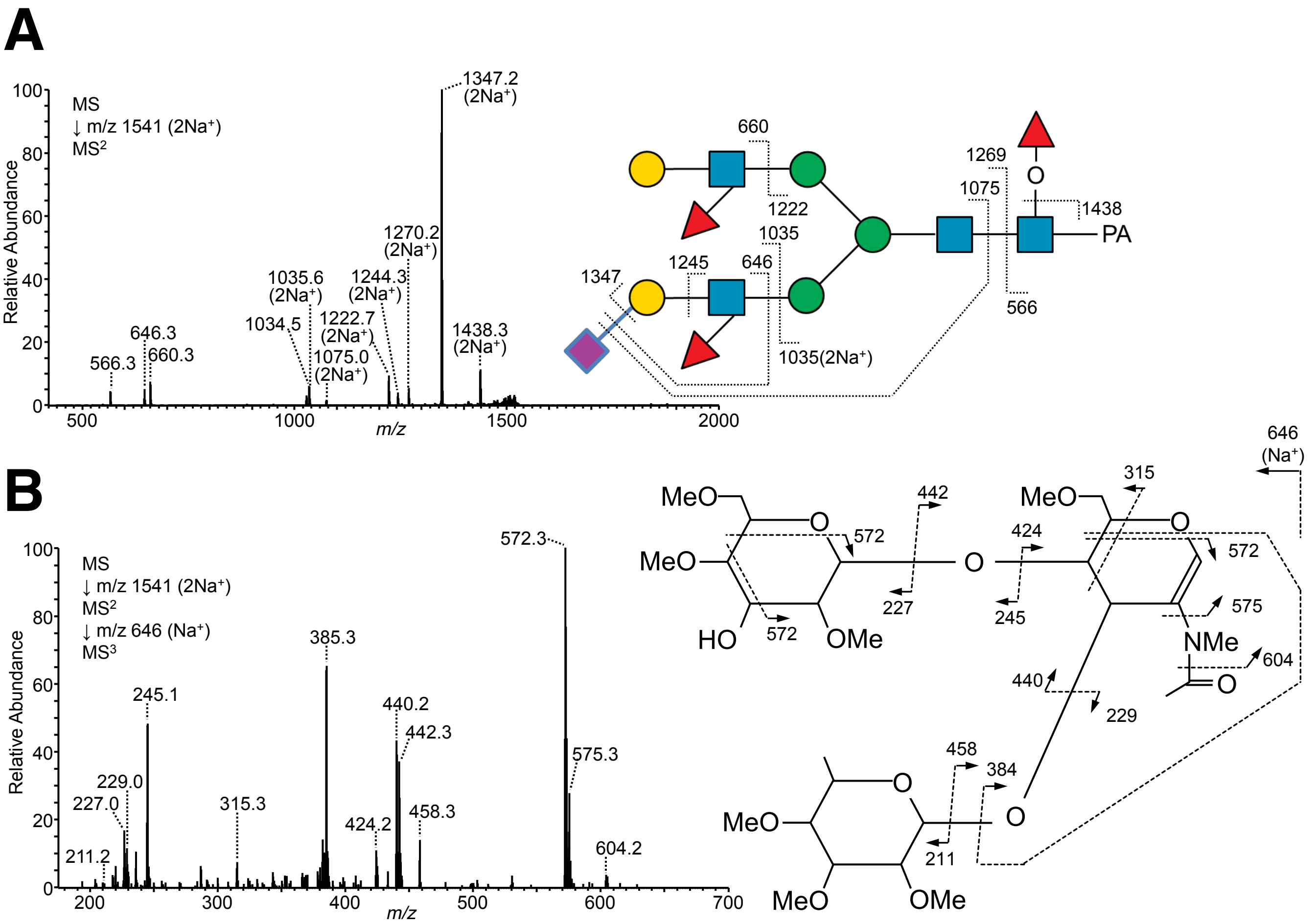

Figure S5-2. MS^n analysis of alkylamidated and permethylated PA-*N*-glycans from chicken trachea. (A) MS^2 spectrum of PA-*N*-glycan with sLe^x from the doubly sodiated precursor ion at *m/z* 1541, corresponding to $Hex_2HexNAc_2Fuc_3(NeuAc+MA)_1C$-PA. The branch positions of each glycan sequence linked to α3- or α6-Man have not been determined. (B) MS^3 spectrum of the B/Y ion fragments $Hex_1HexNAc_1Fuc_1$ at *m/z* 646.

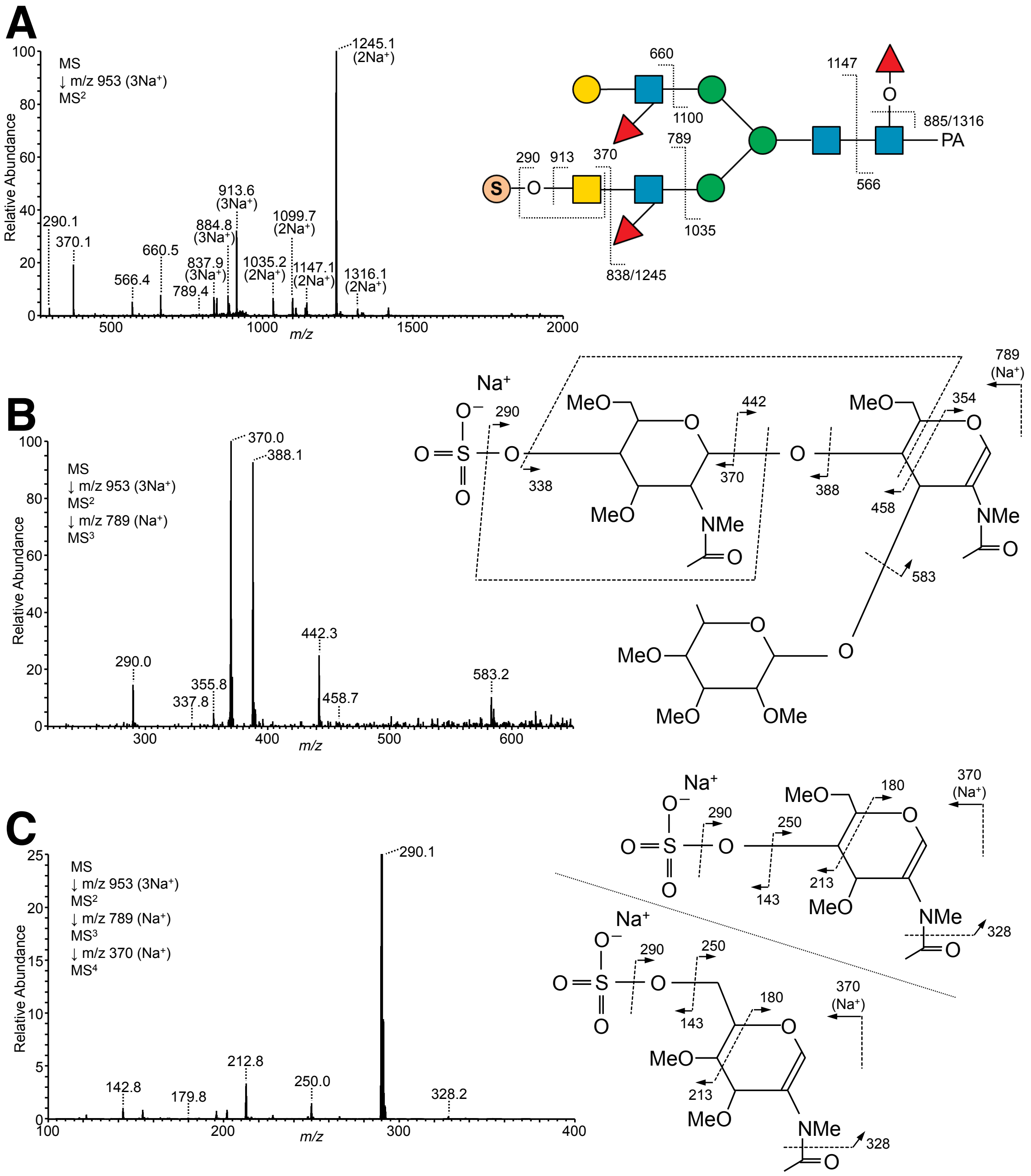

Figure S5-3. MS^n analysis of permethylated PA-*N*-glycans from chicken trachea. (A) MS^2 spectrum of PA-*N*-glycan with sulfated fucosyl LacdiNAc from the triply sodiated precursor ion at *m/z* 953, corresponding to $Hex_1HexNAc_3Fuc_3(SO_3\text{-}H\text{+}Na)_1C\text{-}PA$. The branch positions of each glycan sequence linked to α3- or α6-Man have not been determined. (B) MS^3 spectrum of the B ion fragments $HexNAc_2Fuc_1(SO_3\text{-}H\text{+}Na)_1$ at *m/z* 789. (C) MS^4 spectrum of the B ion fragments $HexNAc_1(SO_3\text{-}H\text{+}Na)_1$ at *m/z* 370.

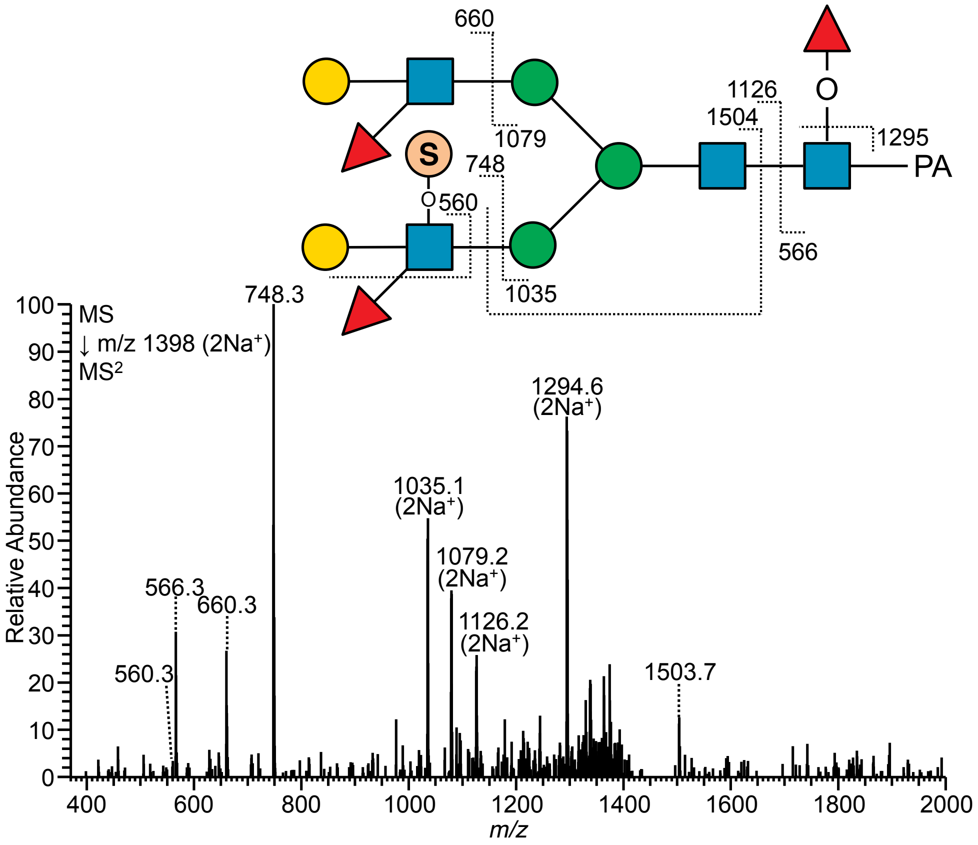

Figure S5-4. MS^2 analysis of permethylated PA-*N*-glycans from chicken trachea. MS^2 spectrum of PA-*N*-glycan with sulfated Le^x from the doubly sodiated precursor ion at *m/z* 1398, corresponding to $Hex_2HexNAc_2Fuc_3(SO_3\text{-}H+Na)_1C$-PA. The branch positions of each glycan sequence linked to α3- or α6-Man have not been determined. The position of sulfate group linked to GlcNAc was deduced based on the elution position of non-permethylated PA-*N*-glycans on reversed-phase LC as shown in Supplementary Figure S6.

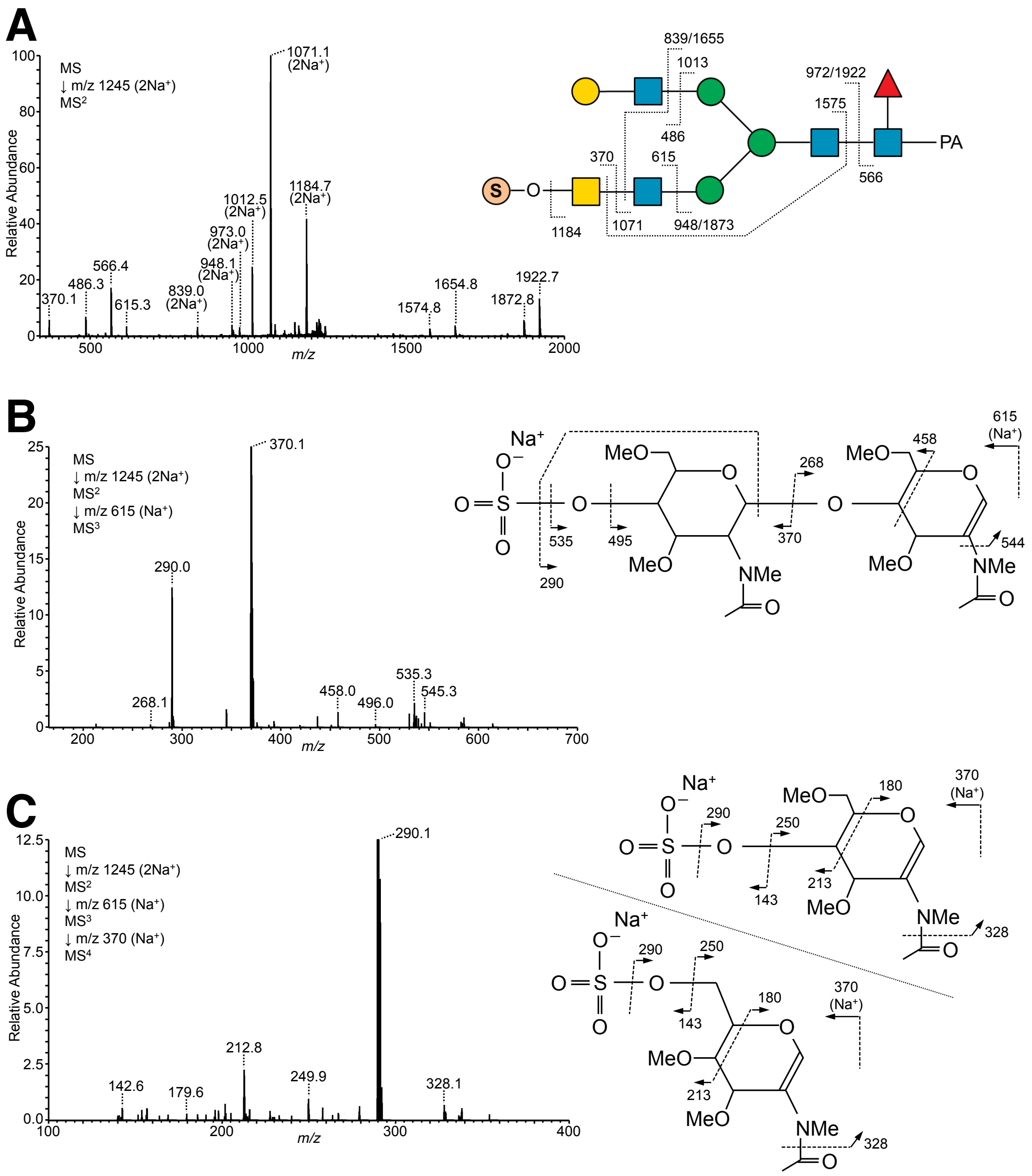

Figure S5-5. MS^n analysis of permethylated PA-*N*-glycans from chicken lung. (A) MS^2 spectrum of PA-*N*-glycan with sulfated LacdiNAc from the doubly sodiated precursor ion at *m/z* 1245, corresponding to $Hex_1HexNAc_3Fuc_1(SO_3\text{-}H\text{+}Na)_1C$-PA. The branch positions of each glycan sequence linked to α3- or α6-Man have not been determined. (B) MS^3 spectrum of the B ion fragments $HexNAc_2(SO_3\text{-}H\text{+}Na)_1$ at *m/z* 615. (C) MS^4 spectrum of the B ion fragments $HexNAc_1(SO_3\text{-}H\text{+}Na)_1$ at *m/z* 370.

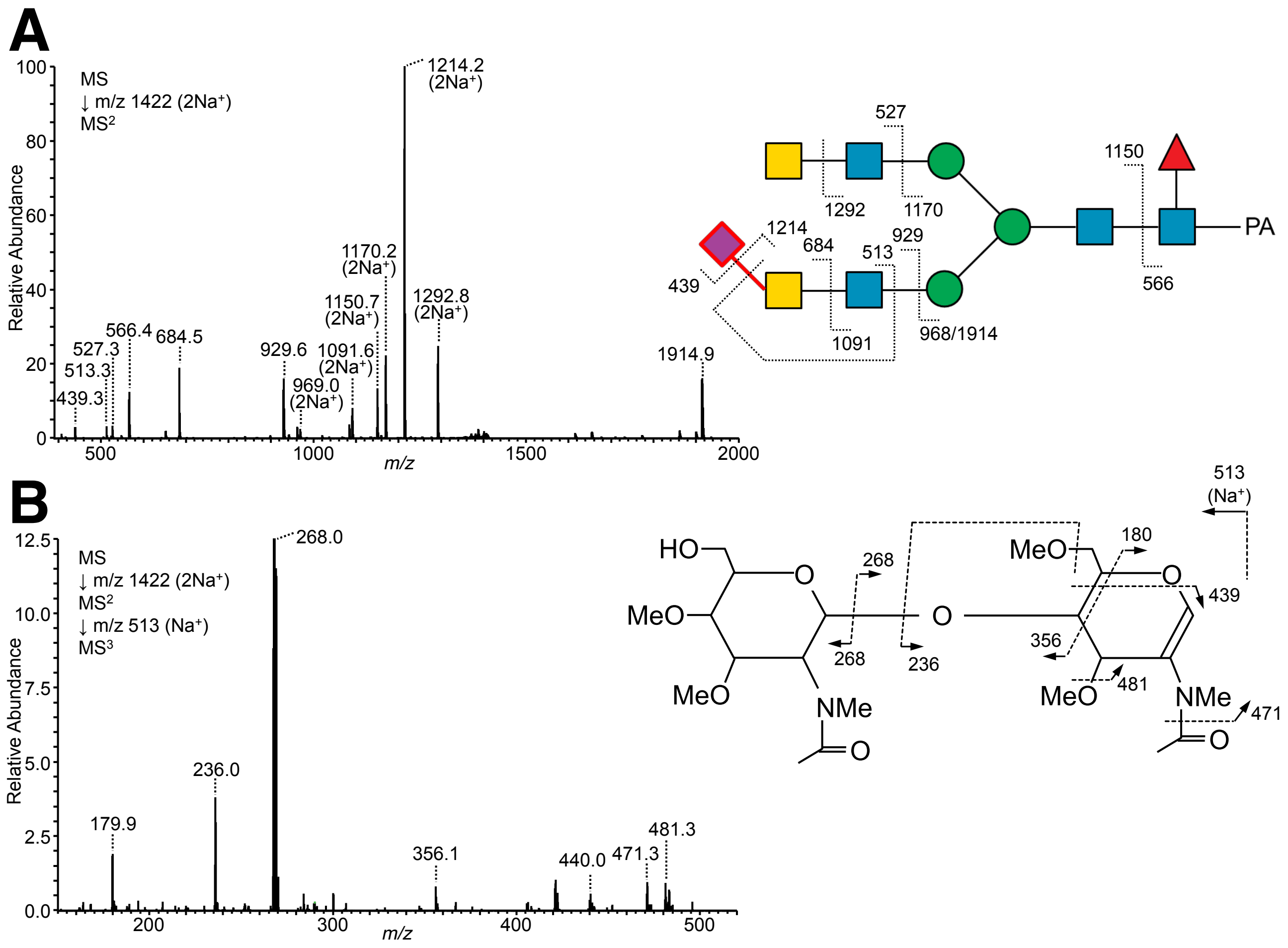

Figure S5-6. MS^n analysis of alkylamidated and permethylated PA-*N*-glycans from chicken lung. (A) MS^2 spectrum of PA-*N*-glycan with sialylated LacdiNAc from the doubly sodiated precursor ion at *m/z* 1422, corresponding to $HexNAc_4Fuc_1(NeuAc+iPA)_1C$-PA. The branch positions of each glycan sequence linked to α3- or α6-Man have not been determined. (B) MS^3 spectrum of the B/Y ion fragments $HexNAc_2$ at *m/z* 513.

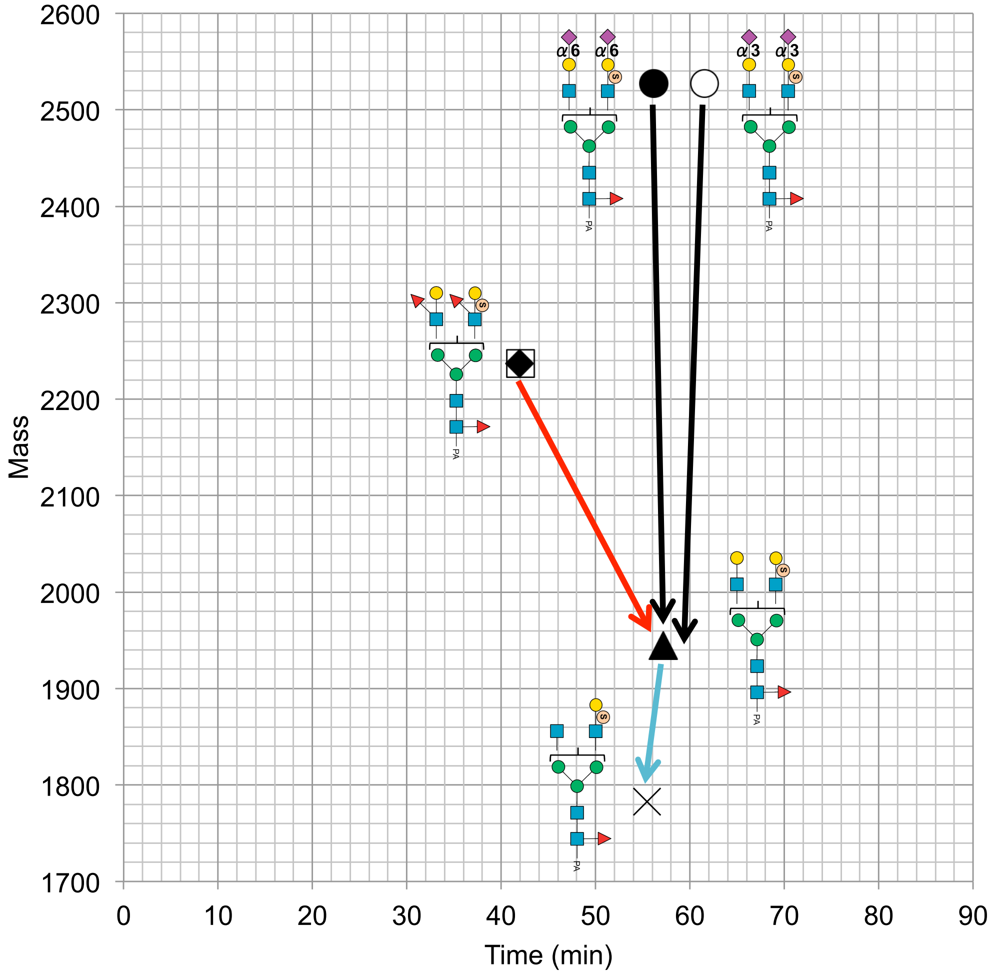

Figure S6. A two-dimensional (2D) map of PA-*N*-glycans with a sulfate group linked to GlcNAc. The elution time (*x*-axis) and the mass value (*y*-axis) of each PA-*N*-glycan from chicken trachea ($Hex_2HexNAc_2Fuc_3(SO_3)_1C$-PA) or lung ($Hex_2HexNAc_2Fuc_1NeuAc_2(SO_3)_1C$-PA) were plotted. Fuc residues on branches were removed by α1-3,4 fucosidase (red arrow). Sia-linkages of disialylated PA-*N*-glycans with a sulfate group were deduced on the elution positions, which are the same as those of PA-*N*-glycans from chicken colon found in previously[13], as well as the results of SALSA (Table S2B) and SALSA/permethylation (Fig. 3). Sia residues were removed by neuraminidase (black arrow). Desialylated or de-α3/4-fucosylated PA-*N*-glycans with a sulfate group (solid triangle) lost one β4-Gal residue by β1-4 galactosidase (sky blue arrow).

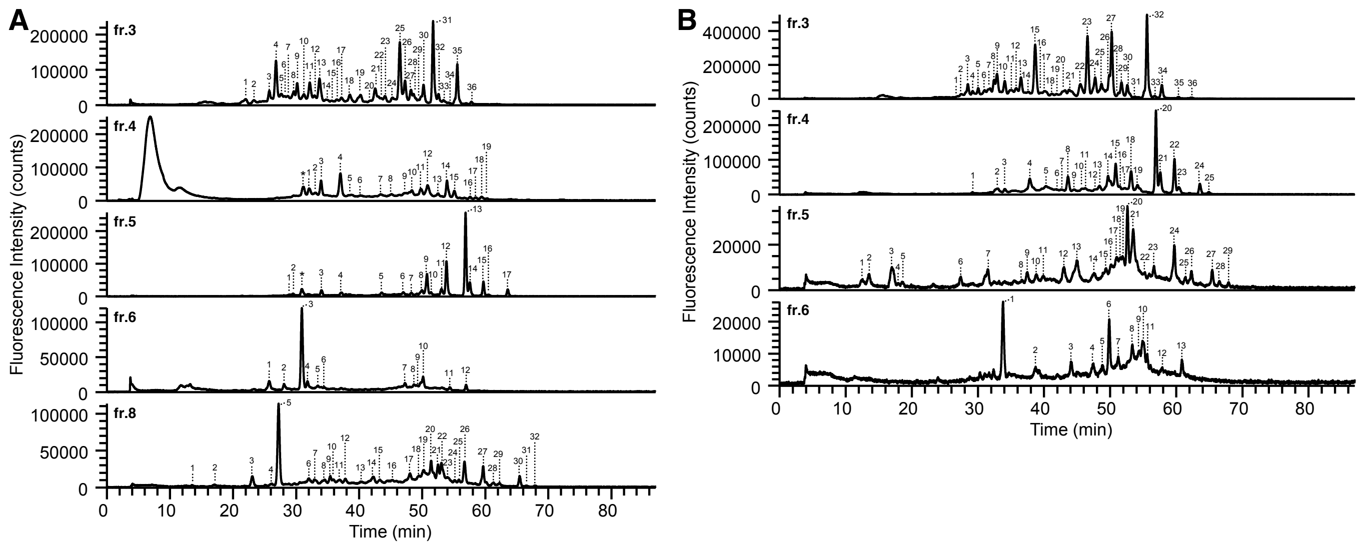

Figure S7. Elution profiles of alkylamidated PA-*N*-glycans from chicken trachea (A) and lung (B) on reversed-phase LC. Each fraction of PA-*N*-glycans from the DEAE column, except for the neutral fraction (fr.1), non-glycan fraction (fr.2), and non-sialylated fraction (fr. 7 of trachea), was analyzed by LC-MS and MS/MS following the SALSA method. Most of the detectable peaks eluted in 10–87 min were numbered, regardless of whether they contain PA-*N*-glycans. The results of LC-MS and MS/MS are summarized in Supplementary Table S2.
