# Supplementary material for: Structural analysis of N-glycans in chicken trachea and lung reveals potential receptors of chicken influenza viruses
Source: Sci Rep. 2022 Feb 8;12:2081. doi: 10.1038/s41598-022-05961-x (PMC8827061; doi:10.1038/s41598-022-05961-x)
Supplement: Supplementary file 2 — Supplementary Information. [file 41598_2022_5961_MOESM2_ESM.pdf]

Supporting information

Structural analysis of *N*-glycans of chicken trachea and lung revealed potential receptors of chicken influenza viruses

Noriko Suzuki[1,]*, Tatsuya Abe[1], and Shunji Natsuka[1,2]

[1]Graduate School of Science and Technology and [2]Faculty of Science, Niigata University, 8050 Ikarashi-nino-cho, Nishi-ku, Niigata 950-2181, Japan

*To whom correspondence should be addressed: E-mail: nrsuzuki@gs.niigata-u.ac.jp

Supplementary Information
Supplementary Figures S1, S2, S3, S4-1–S4-10, S5-1–S5-6, S6, S7
Supplementary Table S1A
Supplementary Table S1B
Supplementary Table S2A
Supplementary Table S2B
Supplementary Table S3
Supplementary Table S4

Supplementary Information

Data of CFG glycan arrays

Following data of influenza viruses derived from chickens were available in the online public data of glycan arrays by the Consortium for Functional Glycomics (CFG) (http://www.functionalglycomics.org/glycomics/publicdata/primaryscreen.jsp), which are now opereted by the National Center for Functional Glycomics (NCFG).

(1) Influenza A Chicken H5N1 hemagglutinin

---Information: A/Chicken H5N1 15ug HA- precomplex Slide#10223 CFG#1759 8/26/2009 Alexa488 Hong

---Mammalian Printed Array: Ver 4.0 (Date: 2010/1/23)

(2) Influenza A virus hemagglutinin (A/Chicken/PA/298101-4/2004) H2N2

---Information: Chicken H2N2 14ug HA precomplex Slide# 12829 CFG# 2066 Alexa488 6/8/10 Hong

---Mammalian Printed Array: Ver 4.1 (Date: 2011/1/22)

(3) Influenza A virus hemagglutinin (A/Chicken/Nanchang/7-010/2000) H3N6

---Information: Chicken H3N6 14 ug HA precomplex Slide# 12884 CFG#2066 Alexa488 6/8/10 Hong

---Mammalian Printed Array: Ver 4.1 (Date: 2011/1/23)

(4) Avian Influenza A/chicken/Pennsylvania/13609/1993 Low pathogenic H5N2

---Information: PA/13609/1993 (1^5pFu/ml) Slide#: 13446 Cfg#:2198 03/11/2011 V4.2 Cy5 CMZ

---Mammalian Printed Array: Ver 4.2 (Date: 2011/6/30)

(5) A/chicken/British Columbia/CN-7/2004 (H7N3) wt_2862

---Information: (H7N3)WT (Dilute with 0.1vol.10%BSA) CFG#2450 Slide#:15142 02/16/2012 Alexa488 CMZ

---Mammalian Printed Array: Ver 5.0 (Date: 2012/5/19)

(6) A/chicken/Hong Kong/G9/1997 (H9N2) wt_2859

---Information: (H9N2)WT (Dilute with 0.1vol.10%BSA) CFG#2450 Slide#:15120 02/14/2012 Alexa488 CMZ

---Mammalian Printed Array: Ver 5.0 (Date: 2012/5/19)

The results of glycan arrays downloaded from the web site were copied and pasted in an Excel file (file name: Table_S4_CFG_glycan_array.xlsx). We selected glycans with 10 to 100% signal intensity (the highest signal intensity was defined as 100%), and checked the presence of the following glycan sequences which are found in chicken trachea and/or lung (except (iv)):

(i) Neu5Acα2-3Galβ1-4GlcNAcβ (α2,3-sialyl LacNAc),
(ii) Neu5Acα2-3Galβ1-4[6OSO$_3$]GlcNAcβ (6-sulfo α2,3-sialyl LacNAc),
(iii) Neu5Acα2-3Galβ1-4(Fucα1-3)GlcNAcβ (sLe^x),
(iv) Neu5Acα2-3Galβ1-4(Fucα1-3)[6OSO$_3$]GlcNAcβ (6-sulfo sLe^x),
(v) Neu5Acα2-3Galβ1-4GlcNAcβ1-3Galβ1-4GlcNAcβ1-3Galβ1-4GlcNAcβ
(α2,3-sialyl LacNAc with LacNAc repeats)

(Notes)

1. Glycans with sequences (i)~(iv) present in Mammalian Printed Array Ver 4.0, 4.1, 4.2 and 5.0, and glycans with sequence (v) present only in Ver 5.0.
2. In Mammalian Printed Array Ver 4.0 and 4.1, sialylated glycans are mainly shorter oligosaccharides in *O*-glycan backbones or in ganglioside structures than in *N*-glycans. In case of sialylated *N*-glycans, only biantennary structures are in the arrays.
3. In Mammalian Printed Array Ver 4.2, one triantennary sialylated *N*-glycan are also included. However, it contains two branches with sialylated type I LacNAc (Neu5Acα2-3Galβ1-3GlcNAcβ1-) and one branch with sialylated type II LAcNAc (Neu5Acα2-3Galβ1-4GlcNAcβ1-), which is not found in chicken trachea and lung.
4. In Mammalian Printed Array Ver 5.0, sialylated *N*-glycans with biantennary, triantennary (both 2,2′,6′-tri and 2,4,2′-tri), and tetraantennary structures are included, and biantennary structures with LacNAc repeats containing two or three LacNAc units are also included.

5. *N*-Glycans with 6-sulfo α2,3-sialyl LacNAc, sLe^x, or 6-sulfo sLe^x are not included in the CFG glycan arrays.
6. The summary of the bindings of chicken influenza A viruses to the glycan sequences (i)~(v) on the CFG glycan arrays are shown in Table S4.

While most of the chicken IAVs preferentially bind to glycans with NeuAcα2-3Gal as a minimum structure, bindings to 6-sulfo α2,3-sialyl LacNAc and sLe^x, or 6-sulfo sLe^x are different among chicken IAVs as shown in Table S4.

(1) H5N1:

strongly binds to 6-sulfo α2,3-sialyl LacNAc.
moderately binds to α2,3-sialyl LacNAc.
weakly binds to sLe^x.
poorly binds to 6-sulfo sLe^x.
weakly binds to biantennary *N*-glycans with α2,3-sialyl LacNAc.

(2) H2N2:

strongly binds to glycans with
Neu5Acα2-3Galβ1-3(Neu5Acα2-3Galβ1-4GlcNAcβ1-6)GalNAcα- or
Neu5Acα2-3Galβ1-3(Neu5Acα2-3Galβ1-4)GlcNAcβ-.
only very weakly or poorly binds to most other glycans regardless the presence of NeuAcα2-3Gal.

(3) H3N6:

strongly binds to α2,3-sialyl LacNAc and 6-sulfo α2,3-sialyl LacNAc.
poorly binds to sLe^x and 6-sulfo sLe^x.
strongly binds to biantennary *N*-glycans with α2,3-sialyl LacNAc.

(4) H5N2:

strongly binds to 6-sulfo α2,3-sialyl LacNAc.
moderately binds to α2,3-sialyl LacNAc.
weakly binds to sLe^x and 6-sulfo sLe^x.
weakly binds to biantennary *N*-glycans with α2,3-sialyl LacNAc.

(5) H7N3:

strongly binds to sLe^x.

moderately binds to α2,3-sialyl LacNAc.

poorly binds to 6-sulfo α2,3-sialyl LacNAc and 6-sulfo sLe^x.

moderately binds to biantennary *N*-glycans with α2,3-sialyl LacNAc.

weakly binds to triantennary *N*-glycans with α2,3-sialyl LacNAc.

poorly binds to tetraantennary *N*-glycans with α2,3-sialyl LacNAc.

weakly binds to α2,3-sialyl LacNAc with LacNAc repeats.

(6) H9N2:

strongly binds to α2,3-sialyl LacNAc.

weakly binds to 6-sulfo α2,3-sialyl LacNAc.

poorly binds to sLe^x and 6-sulfo sLe^x.

weakly binds to bi-, tri-, and tetra-antennary *N*-glycans with α2,3-sialyl LacNAc.

poorly binds to α2,3-sialyl LacNAc with LacNAc repeats.

broad bindings to glycans with either α2,3-Sia, α2,6-Sia, or α2,8-Sia.
