# Supplementary material for: Structural analysis of N-glycans in chicken trachea and lung reveals potential receptors of chicken influenza viruses
Source: Sci Rep. 2022 Feb 8;12:2081. doi: 10.1038/s41598-022-05961-x (PMC8827061; doi:10.1038/s41598-022-05961-x)
Supplement: Supplementary file 3 — Supplementary Table S1. [file 41598_2022_5961_MOESM3_ESM.pdf]

**Table S1A. MS and MS/MS data for PA-*N*-glycans from chicken trachea on LC-MS**

[a] H, hexose; HN, *N*-acetylhexosamine; F, fucose (or deoxyhexose); GN, *N*-acetylglucosamine; M, mannose; NA, *N*-acetylneuraminic acid; SO3, sulfate group; HPO3, phosphate group; C, trimannosyl core; PA, 2-aminopyridine.

[b] Monosaccharide symbols for deduced glycan structures were as follows:

●: Man ○: Gal ●: Glc ■: GlcNAc □: GalNAc ▲: Fuc ◆: NeuAc Ⓢ: sulfate group Ⓟ: phosphate group

[c] Most of the detectable fluorescence peaks eluted in 10–87 min were numbered, but not all peaks were derived from PA-*N*-glycans. The compositions of some peaks could not be determined by MS and MS/MS analysis due to insufficent signals; these cases are indicated as "data not available".

[d] Individual peaks detected by fluorescence sometimes included more than two kinds of PA-glycans with different mass values. In such cases, the proportions were estimated using the ratios of integrated ion intensities for each *m/z* value detected at the corresponding elution time.

[e] Amounts of glycans relative to the most abundant glycan (pk.1-9-1), for which the amount was defined as 100.

[f] xMS, MS data not available; xMS2, MS/MS data not available.

[g] Linkages of sialic acids (α2,3, α2,6) were deduced based on the elution positions and the results of SALSA and/or SALSA/permethylation.

| Fr. No. (DEAE) | Peak. No. (ODS) | Full MS No. | Elusion time max (min) | Elusion time range (min) | Observed parent ion (*m/z* value) | Calculated (*m/z* value) | Estimated adduct | Estimated composition[a), c)] | Deduced glycan structure[b)] | Characteristic fragments[a)] | Relative amounts[d)] | Notes[f), g)] |
|---|---|---|---|---|---|---|---|---|---|---|---|---|
| fr.1 | pk.1-1 | 1 | 14.28 | 13.91-14.54 | 811.33 | 811.35 | M+H+ | H1HN2F1-PA | unknown | | 3.49 | non-*N*-glycan |
| | pk.1-2 | 1 | 14.97 | 14.61-15.30 | 899.77 | 900.33 | M+2H+ | H5C-PA (M8GN2-PA) | 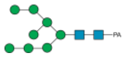 | 325(H2) | 5.87 | artifact (Schiff base by incomplete reduction of pk. 1-7-1) |
| | pk.1-3 | 1 | 15.57 | 15.37-15.72 | 980.13 | 981.36 | M+2H+ | H6C-PA (M9GN2-PA) | 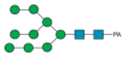 | 325(H2)<br>1151(H4HN2-PA) | 1.19 | artifact (Schiff base by incomplete reduction of pk. 1-9-1) |
| | | 2 | | | 818.74 | 819.31 | M+2H+ | H4C-PA (M7GN2-PA) | 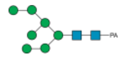 | 325(H2)<br>1151(H4HN2-PA) | 1.02 | artifact (epimer of pk.1-8-1) |
| | pk.1-4 | 1 | 16.09 | 15.79-16.35 | 980.93 | 981.36 | M+2H+ | H6C-PA (M9GN2-PA) | 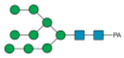 | 325(H2)<br>1151(H4HN2-PA) | 5.23 | artifact (epimer of pk.1-9-1) |
| | pk.1-5 | 1 | 18.04 | 17.68-18.52 | 1142.94 | | | data not available | | | 3.67 | (artifact) |
| | | 2 | | | 737.93 | 738.28 | M+2H+ | H3C-PA (M6GN2-PA) | 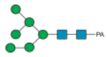 | 325(H2)<br>1151(H4HN2-PA) | 1.82 | artifact (epimer of pk.1-12-1) |
| | pk.1-6 | 1 | 21.54 | 21.24-21.94 | 656.93<br>1313.48 | 657.25<br>1313.50 | M+2H+<br>M+H+ | H2C-PA (M5GN2-PA) | 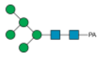 | 325(H2)<br>1151(H4HN2-PA) | 3.55 | artifact (epimer of pk.1-14-1) |
| | | 2 | | | 938.19 | | | data not available | | | 0.93 | non-*N*-glycan (Hex-oligomer) |
| | pk.1-7 | 1 | 23.55 | 22.85-24.17 | 899.96 | 900.33 | M+2H+ | H5C-PA (M8GN2-PA) | 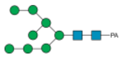 | 325(H2)<br>1151(H4HN2-PA) | 78.84 | |
| | pk.1-8 | 1 | 24.73 | 24.24-25.14 | 818.84 | 819.31 | M+2H+ | H4C-PA (M7GN2-PA) | 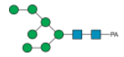 | 325(H2)<br>1151(H4HN2-PA) | 35.46 | |
| | pk.1-9 | 1 | 25.96 | 25.28-26.74 | 981.03 | 981.36 | M+2H+ | H6C-PA (M9GN2-PA) | 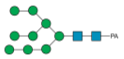 | 325(H2)<br>1151(H4HN2-PA) | 100.00 | |
| | pk.1-10 | 1 | 27.34 | 26.81-27.57 | 899.80 | 900.33 | M+2H+ | H5C-PA (M8GN2-PA) | 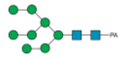 | 325(H2)<br>1151(H4HN2-PA) | 7.04 | |
| | pk.1-11 | 1 | 28.31 | 27.64-28.68 | 818.86<br>1637.60 | 819.31<br>1637.60 | M+2H+<br>M+H+ | H4C-PA (M7GN2-PA) | 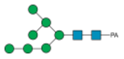 | 325(H2)<br>1151(H4HN2-PA) | 18.31 | |
| | | 2 | | | 912.73 | 912.85 | M+2H+ | H3HN1F1C-PA | 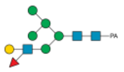 | 366(H1HN1)<br>512(H1HN1F1)<br>1314(H5HN2-PA) | 13.72 | Le[X] |
| | pk.1-12 | 1 | 29.22 | 28.75-30.07 | 737.90<br>1475.52 | 738.28<br>1475.55 | M+3H+<br>M+2H+ | H3C-PA (M6GN2-PA) | 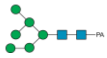 | 325(H2)<br>1151(H4HN2-PA) | 87.43 | |

Table S1A Continued.

| Fr. No. (DEAE) | Peak. No. (ODS) | Full MS No. | Elusion time max (min) | Elusion time range (min) | Observed parent ion (*m/z* value) | Calculated (*m/z* value) | Estimated adduct | Estimated composition[a), c)] | Deduced glycan structure[b)] | Characteristic fragments[a)] | Relative amounts[d)] | Notes[f), g)] |
|---|---|---|---|---|---|---|---|---|---|---|---|---|
| | pk.1-13 | 1 | 31.94 | 31.53-32.43 | 1062.20 | 1062.39 | $M+2H^+$ | H7C-PA (Glc1M9GN2-PA) | 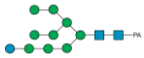 | 325(H2)<br>1151(H4HN2-PA) | 22.42 | |
| | pk.1-14 | 1 | 33.21 | 32.50-33.89 | 656.90<br>1313.45 | 657.25<br>1313.50 | $M+2H^+$<br>$M+H^+$ | H2C-PA (M5GN2-PA) | 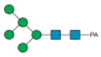 | 325(H2)<br>1151(H4HN2-PA) | 70.14 | |
| | pk.1-15 | 1 | 34.34 | 34.03-34.72 | 900.09 | 900.33 | $M+2H^+$ | H5C-PA (Glc1M7GN2-PA) | 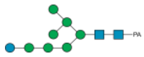 | 325(H2)<br>1151(H4HN2-PA) | 2.40 | |
| | | 2 | | | 1095.69 | 1095.41 | $M+2H^+$ | H4HN2F1C-PA | 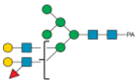 | 366(H1HN1)<br>512(H1HN1F1)<br>1151(H4HN2-PA) | 3.03 | $Le^X$ |
| | pk.1-16 | 1 | 35.43 | 34.93-36.04 | 839.83 | 839.82 | $M+2H^+$ | H3HN1C-PA | 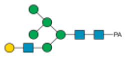 | 366(H1HN1)<br>1151(H4HN2-PA) | 10.25 | |
| | | 2 | | | 1143.03 | 1143.41 | $M+2H^+$ | H8C-PA (Glc2M9GN2-PA) | 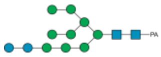 | 325(H2)<br>1151(H4HN2-PA) | 2.04 | |
| | pk.1-17 | 1 | 37.02 | 36.18-37.78 | 1108.22 | 1107.93 | $M+2H^+$ | H2HN3F2C-PA | 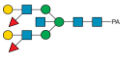 | 366(H1HN1)<br>512(H1HN1F1)<br>1339(H3HN3F1-PA)<br>1542(H3HN4F1-PA) | 20.02 | $Le^X$, $Le^X$ |
| | | 2 | | | 1181.38 | 1180.96 | $M+2H^+$ | H2HN3F3C-PA | 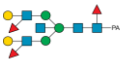 | 366(H1HN1)<br>512(H1HN1F1)<br>1135(F1C-PA)<br>1339(H3HN3F1-PA)<br>1542(H3HN4F1-PA) | 5.24 | $Le^X$, $Le^X$<br>artifact (epimer of pk.1-24-1) |
| | pk.1-18 | 1 | 38.28 | 37.85-38.40 | 985.83 | 985.88 | $M+2H^+$ | H3HN1F2C-PA | 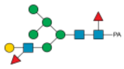 | 366(H1HN1)<br>512(H1HN1F1)<br>1298(H4HN2F1-PA) | 4.36 | $Le^X$ |
| | pk.1-19 | 1 | 38.98 | 38.54-39.24 | 1188.87 | 1188.96 | $M+2H^+$ | H3HN3F2C-PA | 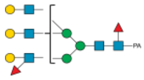 | 366(H1HN1)<br>512(H1HN1F1)<br>1135(F1C-PA) | 7.99 | $Le^X$ |
| | pk.1-20 | 1 | 39.68 | 39.38-40.49 | 1079.90 | 1079.42 | $M+2H^+$ | H2HN2F3C-PA | 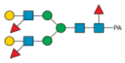 | 366(H1HN1)<br>512(H1HN1F1)<br>973(H2HN2F1-PA)<br>1135(F1C-PA) | 24.64 | $Le^X$, $Le^X$ |
| | pk.1-21 | 1 | 43.81 | 43.40-44.17 | 860.46 | 860.33 | $M+2H^+$ | H2HN2C-PA | 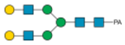 | 366(H1HN1)<br>1192(H3HN3-PA) | 16.86 | Standard E |
| | pk.1-22 | 1 | 44.75 | 44.24-45.49 | 973.41 | 973.40 | $M+H^+$ | H2HN2F1-PA | 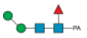 | 446(HN1F1-PA) | 16.44 | |
| | | 2 | | | 1135.44 | 1135.45 | $M+H^+$ | F1C-PA | 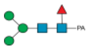 | 446(HN1F1-PA) | 16.15 | |
| | | 3 | | | 1108.50 | 1107.93 | $M+2H^+$ | H2HN3F2C-PA | 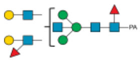 | 366(H1HN1)<br>512(H1HN1F1)<br>1135(F1C-PA) | 2.61 | $Le^X$<br>artifact (epimer of pk.1-28-1 and/or pk.1-29-1) |
| | pk.1-23 | 1 | 46.76 | 44.24-45.49 | 1006.29 | 1006.39 | $M+2H^+$ | H2HN2F2C-PA | 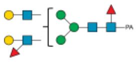 | 366(H1HN1)<br>512(H1HN1F1)<br>1135(F1C-PA)<br>1339(H3HN3F1-PA) | 28.21 | $Le^X$ |
| | pk.1-24 | 1 | 48.04 | 47.22-48.96 | 787.75<br>1181.47 | 787.64<br>1180.96 | $M+3H^+$<br>$M+2H^+$ | H2HN3F3C-PA | 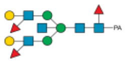 | 366(H1HN1)<br>512(H1HN1F1)<br>1339(H3HN3F1-PA) | 87.51 | $Le^X$, $Le^X$ |
| | pk.1-25 | 1 | 50.19 | 49.51-50.83 | 771.76 | 771.31 | $M+2H^+$ | HN2F1C-PA | 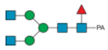 | 446(HN1F1-PA)<br>1339(H3HN3F1-PA) | 20.75 | Standard J |

Table S1A Continued.

| Fr. No. (DEAE) | Peak. No. (ODS) | Full MS No. | Elusion time max (min) | Elusion time range (min) | Observed parent ion (*m/z* value) | Calculated (*m/z* value) | Estimated adduct | Estimated composition[a), c)] | Deduced glycan structure[b)] | Characteristic fragments[a)] | Relative amounts[d)] | | Notes[f), g)] |
|---|---|---|---|---|---|---|---|---|---|---|---|---|---|
| | | 2 | | | 974.07 | 974.39 | M+2H$^+$ | HN4F1C-PA | 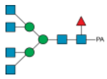 | 1339(H3HN3F1-PA)<br>1542(H3HN4F1-PA) | 2.13 | | |
| | pk.1-26 | 1 | 54.44 | 53.57-55.32 | 933.36 | 933.36 | M+2H$^+$ | H2HN2F1C-PA | 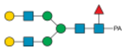 | 366(H1HN1)<br>446(HN1F1-PA)<br>1339(H3HN3F1-PA) | 28.77 | | Standard F |
| | pk.1-27 | 1 | 57.48 | 56.53-58.75 | 1026.88 | 1026.90 | M+2H$^+$ | H1HN3F2C-PA | 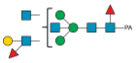 | 366(H1HN1)<br>512(H1HN1F1)<br>1542(H3HN4F1-PA) | 16.67 | | Le$^X$ |
| | | 2 | | | 880.88 | 880.85 | M+2H$^+$ | H1HN3C-PA | 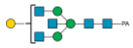 | 366(H1HN1)<br>1192(H3HN3-PA) | 3.29 | | |
| | | 3 | | | 1217.79 | 1217.47 | M+2H$^+$ | H3HN4F1C-PA | 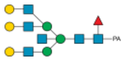 | 366(H1HN1)<br>1339(H3HN3F1-PA)<br>1542(H3HN4F1-PA) | 7.71 | | |
| | pk.1-28 | 1 | 59.89 | 59.03-60.97 | 739.03<br>1108.47 | 738.96<br>1107.93 | M+3H$^+$<br>M+2H$^+$ | H2HN3F2C-PA | 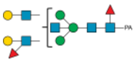 | 366(H1HN1)<br>512(H1HN1F1)<br>1339(H3HN3F1-PA)<br>1542(H3HN4F1-PA) | 21.41 | | Le$^X$ |
| | | 2 | | | 962.40 | 961.87 | M+2H$^+$ | H2HN3C-PA | 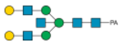 | 366(H1HN1)<br>1192(H3HN3-PA) | 12.39 | | Standard G |
| | pk.1-29 | 1 | 61.91 | 61.18-62.57 | 738.95<br>1107.90 | 738.96<br>1107.93 | M+3H$^+$<br>M+2H$^+$ | H2HN3F2C-PA | 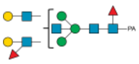 | 366(H1HN1)<br>512(H1HN1F1)<br>1339(H3HN3F1-PA)<br>1542(H3HN4F1-PA) | 25.14 | | Le$^X$ |
| | pk.1-30 | 1 | 69.23 | 68.68-69.93 | 872.83 | 872.85 | M+2H$^+$ | HN3F1C-PA | 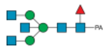 | 446(HN1F1-PA)<br>1339(H3HN3F1-PA)<br>1542(H3HN4F1-PA) | 9.12 | | Standard L |
| | pk.1-31 | 1 | 71.49 | 70.83-72.01 | 954.31 | 953.87 | M+2H$^+$ | H1HN3F1C-PA | 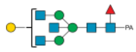 | 366(H1HN1)<br>446(HN1F1-PA)<br>1339(H3HN3F1-PA)<br>1542(H3HN4F1-PA) | 11.23 | | |
| | pk.1-32 | 1 | 72.64 | 72.26-72.81 | 953.85 | 953.87 | M+2H$^+$ | H1HN3F1C-PA | 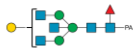 | 366(H1HN1)<br>446(HN1F1-PA)<br>1135(F1C-PA)<br>1339(H3HN3F1-PA)<br>1542(H3HN4F1-PA) | 11.99 | | |
| | pk.1-33 | 1 | 74.28 | 73.33-75.00 | 690.50<br>1034.85 | 690.27<br>1034.90 | M+3H$^+$<br>M+2H$^+$ | H2HN3F1C-PA | 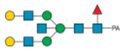 | 366(H1HN1)<br>446(HN1F1-PA)<br>512(H1HN1F1)<br>1135(F1C-PA)<br>1339(H3HN3F1-PA) | 43.37 | | Standard H |
| | pk.1-34 | 1 | 76.52 | 76.25-77.01 | 1115.99 | 1115.93 | M+2H$^+$ | H3HN3F1C-PA | 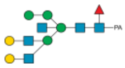 | 366(H1HN1)<br>1542(H3HN4F1-PA) | 3.40 | | |
| | pk.1-35 | 1 | 83.67 | 83.30-84.21 | 860.77<br>1290.85 | | | data not available | | | 3.30 | | xMS2 |
| | pk.1-36 | 1 | 85.46 | 83.30-84.21 | 812.22<br>1217.91 | | | data not available | | | 3.31 | | xMS2 |
| | | | | | | | | | | | | | |
| fr.3 | pk.3-1 | 1 | 17.47 | 17.12-17.68 | 899.38 | 899.36 | M+H$^+$ | H1HN1F1NA1-PA | unknown | | 2.30 | | non-*N*-glycan |
| | | 2 | | | 1113.91 | 1113.92 | M+2H$^+$ | H2HN2F2NA1C-OH | 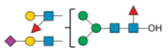 | 366(H1HN1)<br>657(H1HN1NA1)<br>803(H1HN1F1NA1) | - | - | non-PA-glycan (reduced form of pk.3-19-1) |
| | | 3 | | | 1069.41 | 1069.40 | M+2H$^+$ | H2HN3NA1C-OH | 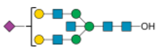 | 366(H1HN1)<br>657(H1HN1NA1) | - | - | non-PA-glycan (reduced form of pk.3-26-2) |

Table S1A Continued.

| Fr. No. (DEAE) | Peak. No. (ODS) | Full MS No. | Elusion time max (min) | Elusion time range (min) | Observed parent ion (*m/z* value) | Calculated (*m/z* value) | Estimated adduct | Estimated composition[a), c)] | Deduced glycan structure[b)] | Characteristic fragments[a)] | Relative amounts[d)] | Notes[f), g)] |
|---|---|---|---|---|---|---|---|---|---|---|---|---|
| | pk.3-2 | 1 | 18.07 | 17.75-18.87 | 880.05 | 879.80 | $M+2H^+$ | H3HN1(HPO3)1C-PA | 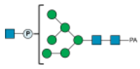 | 405(H2(HPO3)1)<br>1151(H4HN2-PA) | 10.04 | HPO3 |
| | pk.3-3 | 1 | 19.32 | 19.01-19.78 | 1041.59 | 1041.86 | $M+2H^+$ | H5HN1(HPO3)1C-PA | 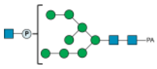 | 405(H2(HPO3)1)<br>1151(H4HN2-PA) | 4.30 | HPO3 |
| | pk.3-4 | 1 | 20.25 | 22.99-23.62 | 960.42 | 960.83 | $M+2H^+$ | H4HN1(HPO3)1C-PA | 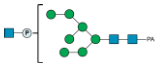 | 405(H2(HPO3)1)<br>1151(H4HN2-PA) | 3.67 | HPO3 |
| | pk.3-5 | 1 | 23.29 | 23.12-23.43 | 879.82 | 879.80 | $M+2H^+$ | H3HN1(HPO3)1C-PA | 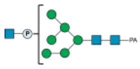 | 1192(H3HN3-PA) | 2.01 | HPO3 |
| | | 2 | | | 1288.39 | 1288.49 | $M+2H^+$ | H2HN3F3NA1C-OH | 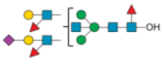 | 366(H1HN1)<br>657(H1HN1NA1)<br>803(H1HN1F1NA1) | - - | non-PA-glycan (reduced form of pk.3-24-1 and/or pk.3-25-2) |
| | pk.3-6 | 1 | 30.37 | 29.91-30.89 | 1142.48 | 1142.43 | $M+2H^+$ | H2HN3F1NA1C-OH | 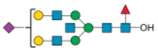 | 366(H1HN1)<br>657(H1HN1NA1) | - - | non-PA-glycan (reduced form of pk.3-30-1) |
| | | 2 | | | 973.14 | 973.34 | $M+2H^+$ | H2HN2F1(SO3)1C-PA | 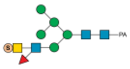 | 407(HN2)<br>553(HN2F1)<br>663(HN2F1(SO3)1)<br>1151(H4HN2-PA)<br>1135(F1C-PA) | 3.97 | LacdiNAc with Fuc and SO3 |
| | pk.3-7 | 1 | 31.63 | 30.96-32.50 | 1066.58 | 1066.88 | $M+2H^+$ | H1HN3F2(SO3)1C-PA | 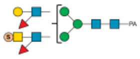 | 366(H1HN1)<br>553(HN2F1)<br>663(HN2F1(SO3)1)<br>1135(F1C-PA) | 4.14 | $Le^X$,<br>LacdiNAc with Fuc and SO3 |
| | | 2 | | | 892.14 | 892.31 | $M+2H^+$ | H1HN2F1(SO3)1C-PA | 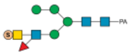 | 407(HN2)<br>553(HN2F1)<br>1151(H4HN2-PA) | 2.08 | LacdiNAc with Fuc and SO3 |
| | pk.3-8 | 1 | 33.23 | 32.57-33.61 | 1006.27 | 1005.88 | $M+2H^+$ | H2HN2NA1C-PA | 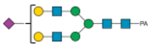 | 366(H1HN1)<br>657(H1HN1NA1) | 4.59 | α2,6-Sia artifact (epimer of pk.3-16-1) |
| | pk.3-9 | 1 | 37.07 | 36.39-37.29 | 705.84<br>1058.32 | 705.93<br>1058.40 | $M+3H^+$<br>$M+2H^+$ | H3HN1F1NA1C-PA | 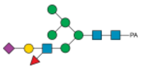 | 366(H1HN1)<br>657(H1HN1NA1)<br>803(H1HN1F1NA1)<br>1314(H5HN2-PA) | 10.58 | $sLe^X$ |
| | | 2 | | | 792.53<br>1188.56 | 792.63<br>1188.45 | $M+3H^+$<br>$M+2H^+$ | H3HN3NA1C-PA | 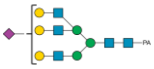 | 366(H1HN1)<br>657(H1HN1NA1) | 2.96 | α2,6-Sia |
| | pk.3-10 | 1 | 37.54 | 37.36-37.85 | 985.77 | 985.37 | $M+2H^+$ | H3HN1NA1C-PA | 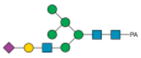 | 366(H1HN1)<br>657(H1HN1NA1)<br>1314(H5HN2-PA) | 7.95 | α2,6-Sia |
| | | 2 | | | 823.75 | 823.31 | $M+2H^+$ | H1HN1NA1C-PA | 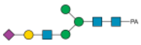 | 366(H1HN1)<br>657(H1HN1NA1)<br>989(H3HN2-PA) | 1.37 | α2,6-Sia |
| | pk.3-11 | 1 | 38.19 | 37.99-38.82 | 1079.42 | 1078.91 | $M+2H^+$ | H2HN2F1NA1C-PA | 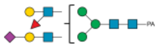 | 366(H1HN1)<br>512(H1HN1F1)<br>657(H1HN1NA1)<br>1192(H3HN3-PA) | 7.12 | $Le^X$<br>α2,6-Sia |
| | | 2 | | | 904.19 | 904.34 | $M+2H^+$ | H2HN1NA1C-PA | 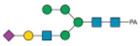 | 366(H1HN1)<br>657(H1HN1NA1)<br>1151(H4HN2-PA) | 4.60 | α2,6-Sia |
| | | 3 | | | 1152.25 | 1151.94 | $M+2H^+$ | H2HN2F2NA1C-PA | 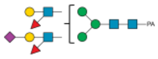 | 366(H1HN1)<br>512(H1HN1F1)<br>803(H1HN1F1NA1)<br>1192(H3HN3-PA) | 1.47 | $Le^X$, $sLe^X$ |
| | pk.3-12 | 1 | 39.20 | 38.89-40.21 | 1225.32 | | | data not available | | | 8.35 | xMS2 |

Table S1A Continued.

| Fr. No. (DEAE) | Peak. No. (ODS) | Full MS No. | Elusion time max (min) | Elusion time range (min) | Observed parent ion (*m/z* value) | Calculated (*m/z* value) | Estimated adduct | Estimated composition[a), c)] | Deduced glycan structure[b)] | Characteristic fragments[a)] | Relative amounts[d)] | Notes[f), g)] |
|---|---|---|---|---|---|---|---|---|---|---|---|---|
| | pk.3-13 | 1 | 41.39 | 40.49-41.53 | 985.34 | 985.37 | M+2H$^+$ | H3HN1NA1C-PA | 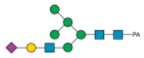 | 366(H1HN1)<br>657(H1HN1NA1)<br>1314(H5HN2-PA) | 3.47 | α2,3-Sia |
| | | 2 | | | 1151.82 | 1151.94 | M+2H$^+$ | H2HN2F2NA1C-PA | 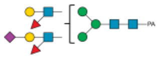 | 512(H1HN1F1)<br>657(H1HN1NA1) | 1.45 | Le$^X$, sLe$^X$ |
| | | 3 | | | 1046.93 | 1046.91 | M+2H$^+$ | data not available | | | 1.44 | xMS2 |
| | pk.3-14 | 1 | 42.26 | 41.74-42.99 | 1140.34 | 1139.91 | M+2H$^+$ | H1HN3F3(SO3)1C-PA | 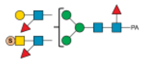 | 366(H1HN1)<br>407(HN2)<br>512(H1HN1F1)<br>553(HN2F1)<br>663(HN2F1(SO3)1) | 35.75 | Le$^X$,<br>LacdiNAc with Fuc and SO3 |
| | | 2 | | | 1119.37 | 1119.40 | M+2H$^+$ | H2HN2F3(SO3)1C-PA | 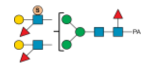 | 366(H1HN1)<br>446(H1HN1\(SO3)1)<br>512(H1HN1F1)<br>592(H1HN1F1(SO3)1) | 1.68 | Le$^X$,<br>Le$^X$ with SO3 |
| | pk.3-15 | 1 | 43.22 | 43.06-44.03 | 965.18 | 965.34 | M+2H$^+$ | H1HN2F2(SO3)1C-PA | 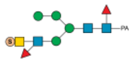 | 533(HN2F1)<br>663(HN2F1(SO3)1)<br>1135(F1C-PA) | 6.20 | LacdiNAc with Fuc and SO3 |
| | pk.3-16 | 1 | 44.91 | 44.17-46.04 | 671.17<br>1006.49 | 670.92<br>1005.88 | M+3H$^+$<br>M+2H$^+$ | H2HN2NA1C-PA | 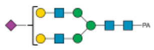 | 366(H1HN1)<br>657(H1HN1NA1)<br>1192(H3HN3-PA) | 64.01 | Standard a<br>α2,6-Sia |
| | | 2 | | | 1180.49 | 1180.45 | M+2H$^+$ | H2HN3F1NA1C-PA | 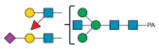 | 366(H1HN1)<br>512(H1HN1F1)<br>657(H1HN1NA1)<br>803(H1HN1F1NA1)<br>1192(H3HN3-PA) | 6.30 | Le$^X$<br>α2,6-Sia |
| | | 3 | | | 1078.73 | 1078.91 | M+2H$^+$ | H2HN2F1NA1C-PA | 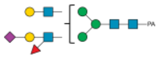 | 366(H1HN1)<br>657(H1HN1NA1)<br>803(H1HN1F1NA1)<br>1192(H3HN3-PA) | 1.29 | sLe$^X$ |
| | pk.3-17 | 1 | 46.72 | 46.18-47.15 | 816.87<br>1224.83 | 816.98<br>1224.97 | M+3H$^+$<br>M+2H$^+$ | H2HN2F3NA1C-PA | 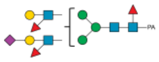 | 366(H1HN1)<br>657(H1HN1NA1)<br>803(H1HN1F1NA1)<br>1339(H3HN3F1-PA) | 12.44 | Le$^X$, sLe$^X$ |
| | pk.3-18 | 1 | 47.89 | 47.22-48.13 | 817.10<br>1225.30 | 816.98<br>1224.97 | M+3H$^+$<br>M+2H$^+$ | H2HN2F3NA1C-PA | 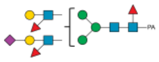 | 366(H1HN1)<br>657(H1HN1NA1)<br>803(H1HN1F1NA1)<br>1339(H3HN3F1-PA) | 13.73 | Le$^X$, sLe$^X$ |
| | | 2 | | | 1261.85 | 1261.48 | M+2H$^+$ | H3HN3F1NA1C-PA | 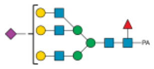 | 366(H1HN1)<br>657(H1HN1NA1)<br>1339(H3HN3F1-PA) | 3.31 | α2,6-Sia |
| | pk.3-19 | 1 | 48.67 | 48.20-48.96 | 768.48<br>1152.30 | 768.29<br>1151.94 | M+3H$^+$<br>M+2H$^+$ | H2HN2F2NA1C-PA | 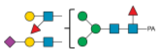 | 366(H1HN1)<br>512(H1HN1F1)<br>657(H1HN1NA1)<br>1135(F1C-PA) | 18.75 | Le$^X$<br>α2,6-Sia |
| | pk.3-20 | 1 | 49.42 | 49.17-50.56 | 1006.41 | 1005.88 | M+2H$^+$ | H2HN2NA1C-PA | 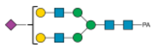 | 366(H1HN1)<br>657(H1HN1NA1)<br>1192(H3HN3-PA) | 9.76 | Standard c<br>α2,3-Sia |
| | | 2 | | | 896.40 | 896.34 | M+2H$^+$ | H1HN1F1NA1C-PA | 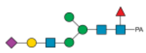 | 366(H1HN1)<br>657(H1HN1NA1)<br>1135(F1C-PA)<br>1339(H3HN3F1-PA) | 6.78 | α2,6-Sia |
| | pk.3-21 | 1 | 51.82 | 51.46-52.22 | 792.83<br>1188.55 | 792.63<br>1188.45 | M+3H$^+$<br>M+2H$^+$ | H3HN3NA1C-PA | 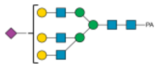 | 366(H1HN1)<br>657(H1HN1NA1)<br>1192(H3HN3-PA) | 8.38 | α2,6-Sia |
| | pk.3-22 | 1 | 52.68 | 52.29-53.34 | | | | data not available | | | 10.25 | xMS2 |
| | pk.3-23 | 1 | 54.01 | 53.54-54.17 | 1180.52 | 1180.45 | M+2H$^+$ | H2HN3F1NA1C-PA | 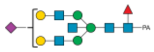 | 366(H1HN1)<br>657(H1HN1NA1)<br>1339(H3HN3F1-PA)<br>1542(H3HN4F1-PA) | 3.67 | α2,6-Sia<br>artifact (epimer of pk.3-30-1) |

Table S1A Continued.

| Fr. No. (DEAE) | Peak. No. (ODS) | Full MS No. | Elusion time max (min) | Elusion time range (min) | Observed parent ion (*m/z* value) | Calculated (*m/z* value) | Estimated adduct | Estimated composition[a), c)] | Deduced glycan structure[b)] | Characteristic fragments[a)] | Relative amounts[d)] | Notes[f), g)] |
|---|---|---|---|---|---|---|---|---|---|---|---|---|
| | | 2 | | | 909.12<br>1363.45 | 909.01<br>1363.01 | $M+3H^+$<br>$M+2H^+$ | data not available | | | 2.32 | xMS2 |
| | pk.3-24 | 1 | 54.93 | 54.38-55.63 | 884.78<br>1326.49 | 884.67<br>1326.51 | $M+3H^+$<br>$M+2H^+$ | H2HN3F3NA1C-PA | 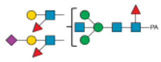 | 366(H1HN1)<br>657(H1HN1NA1)<br>803(H1HN1F1NA1)<br>1339(H3HN3F1-PA) | 19.02 | $Le^X$, $sLe^X$ |
| | pk.3-25 | 1 | 56.21 | 55.70-56.46 | 719.88<br>1079.00 | 719.61<br>1078.91 | $M+3H^+$<br>$M+2H^+$ | H2HN2F1NA1C-PA | 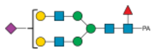 | 366(H1HN1)<br>657(H1HN1NA1)<br>1135(F1C-PA)<br>1339(H3HN3F1-PA) | 8.64 | α2,6-Sia |
| | | 2 | | | 884.47<br>1326.91 | 884.67<br>1326.51 | $M+3H^+$<br>$M+2H^+$ | H2HN3F3NA1C-PA | 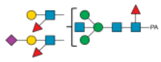 | 657(H1HN1NA1)<br>803(H1HN1F1NA1)<br>1542(H3HN4F1-PA) | 5.81 | $Le^X$, $sLe^X$ |
| | pk.3-26 | 1 | 57.32 | 56.53-59.10 | 836.22<br>1254.01 | 835.99<br>1253.48 | $M+3H^+$<br>$M+2H^+$ | H2HN3F2NA1C-PA | 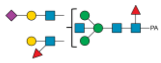 | 366(H1HN1)<br>512(H1HN1F1)<br>657(H1HN1NA1)<br>1339(H3HN3F1-PA)<br>1542(H3HN4F1-PA) | 73.24 | $Le^X$<br>α2,6-Sia |
| | | 2 | | | 1107.54 | 1107.42 | $M+2H^+$ | H2HN3NA1C-PA | 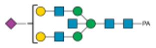 | 366(H1HN1)<br>657(H1HN1NA1) | 6.02 | α2,6-Sia |
| | | 3 | | | 890.51<br>1335.29 | 890.01<br>1334.50 | $M+3H^+$<br>$M+2H^+$ | H3HN3F2NA1C-PA | 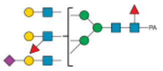 | 366(H1HN1)<br>512(H1HN1F1)<br>657(H1HN1NA1)<br>1339(H3HN3F1-PA) | 4.23 | $Le^X$<br>α2,6-Sia |
| | pk.3-27 | 1 | 62.10 | 61.53-62.99 | 1078.90 | 1078.91 | $M+2H^+$ | H2HN2F1NA1C-PA | 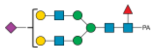 | 366(H1HN1)<br>657(H1HN1NA1)<br>1135(F1C-PA)<br>1339(H3HN3F1-PA)<br>1542(H3HN4F1-PA) | 10.78 | α2,3-Sia |
| | pk.3-28 | 1 | 66.18 | 65.56-67.09 | 1006.89<br>1509.01 | 1006.38<br>1509.07 | $M+3H^+$<br>$M+2H^+$ | H3HN4F3NA1C-PA | 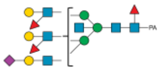 | 366(H1HN1)<br>512(H1HN1F1)<br>657(H1HN1NA1)<br>1542(H3HN4F1-PA) | 7.08 | $Le^X$, $Le^X$<br>α2,6-Sia |
| | pk.3-29 | 1 | 68.80 | 68.06-69.52 | 836.26<br>1253.58 | 835.99<br>1253.48 | $M+3H^+$<br>$M+2H^+$ | H2HN3F2NA1C-PA | 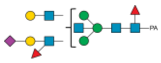 | 366(H1HN1)<br>657(H1HN1NA1)<br>803(H1HN1F1NA1)<br>1542(H3HN4F1-PA) | 4.58 | $sLe^X$ |
| | | 2 | | | 1099.70 | 1099.42 | $M+2H^+$ | H1HN3F1NA1C-PA | 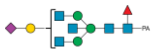 | 366(H1HN1)<br>657(H1HN1NA1)<br>1135(F1C-PA)<br>1339(H3HN3F1-PA)<br>1542(H3HN4F1-PA) | 3.47 | α2,6-Sia |
| | pk.3-30 | 1 | 72.47 | 71.81-73.20 | 787.52<br>1180.96 | 787.30<br>1180.45 | $M+3H^+$<br>$M+2H^+$ | H2HN3F1NA1C-PA | 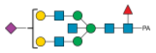 | 366(H1HN1)<br>657(H1HN1NA1)<br>1135(F1C-PA)<br>1339(H3HN3F1-PA)<br>1542(H3HN4F1-PA) | 28.59 | Standard g<br>α2,6-Sia |
| | pk.3-31 | 1 | 73.48 | 73.21-73.74 | 1262.00 | 1261.48 | $M+2H^+$ | H3HN3F1NA1C-PA | 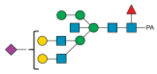 | 366(H1HN1)<br>657(H1HN1NA1)<br>1339(H3HN3F1-PA) | 1.18 | α2,6-Sia |
| | pk.3-32 | 1 | 77.97 | 77.22-78.62 | 1180.37 | 1180.45 | $M+2H^+$ | H2HN3F1NA1C-PA | 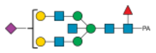 | 366(H1HN1)<br>657(H1HN1NA1)<br>1542(H3HN4F1-PA) | 5.50 | α2,3-Sia |
| | pk.3-33 | 1 | 80.13 | 79.74-81.00 | 909.24<br>1363.57 | 909.01<br>1363.01 | $M+3H^+$<br>$M+2H^+$ | H3HN4F1NA1C-PA | 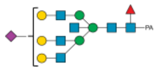 | 366(H1HN1)<br>657(H1HN1NA1)<br>1339(H3HN3F1-PA) | 3.04 | α2,6-Sia |
| | | 2 | | | 1180.26 | 1180.45 | $M+2H^+$ | H2HN3F1NA1C-PA | 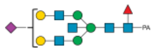 | 366(H1HN1)<br>657(H1HN1NA1)<br>1339(H3HN3F1-PA) | 2.06 | α2,3-Sia |
| | pk.3-34 | 1 | 81.94 | 81.94-82.67 | 1026.99 | | | data not available | | | 2.94 | xMS2 |
| | | | | | | | | | | | | |
| fr.4 | pk.4-1 | 1 | 16.17 | 15.72-16.49 | 550.32 | | | data not available | | | 6.69 | xMS |

Table S1A Continued.

| Fr. No. (DEAE) | Peak. No. (ODS) | Full MS No. | Elusion time max (min) | Elusion time range (min) | Observed parent ion (*m/z* value) | Calculated (*m/z* value) | Estimated adduct | Estimated composition[a), c)] | Deduced glycan structure[b)] | Characteristic fragments[a)] | Relative amounts[d)] | Notes[f), g)] |
|---|---|---|---|---|---|---|---|---|---|---|---|---|
| | pk.4-2 | 1 | 16.77 | 16.56-17.05 | 753.31 | 753.30 | $M+H^+$ | H1HN1NA1-PA | unknown | | 1.54 | non-*N*-glycan |
| | pk.4-3 | 1 | 17.42 | 17.19-17.75 | 899.40 | 899.36 | $M+H^+$ | H1HN1F1NA1-PA | unknown | | 4.07 | non-*N*-glycan |
| | pk.4-4 | 1 | 19.06 | 18.73-19.64 | 591.30 | | | | data not available | | 2.95 | xMS2 |
| | | 2 | | | 753.37 | 753.30 | $M+H^+$ | H1HN1NA1-PA | unknown | | 0.55 | non-*N*-glycan |
| | pk.4-5 | 1 | 22.35 | 21.94-22.85 | 753.34 | 753.30 | $M+H^+$ | H1HN1NA1-PA | unknown | | 1.23 | non-*N*-glycan |
| | pk.4-6 | 1 | 38.24 | 37.88-38.92 | 775.42 | | | | data not available | | 2.02 | non-glycan |
| | pk.4-7 | 1 | 45.80 | 45.21-46.26 | 1084.49 | | | | data not available | | 4.46 | xMS2 |
| | pk.4-8 | 1 | 48.24 | 45.73-48.92 | 1152.54 | | | | data not available | | 3.93 | xMS2 |
| | | 2 | | | 1084.87 | | | | data not available | | 3.53 | xMS2 |
| | pk.4-9 | 1 | 49.68 | 48.99-50.24 | 1035.73 | | | | data not available | | 4.97 | xMS2 |
| | | 2 | | | 1206.38 | | | | data not available | | 4.05 | xMS2 |
| | pk.4-10 | 1 | 52.85 | 52.01-53.61 | 914.47<br>1370.50 | 914.01<br>1370.51 | $M+3H^+$<br>$M+2H^+$ | H2HN2F3NA2C-PA | 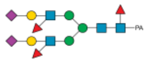 | 366(H1HN1)<br>657(H1HN1NA1)<br>803(H1HN1F1NA1)<br>1135(F1C-PA) | 13.80 | sLe$^X$, sLe$^X$<br>(identical to pk.5-6-1) |
| | pk.4-11 | 1 | 54.28 | 53.67-55.28 | 1035.78 | 1035.72 | $M+3H^+$ | H3HN3F3NA2C-PA | 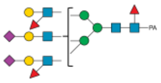 | 366(H1HN1)<br>657(H1HN1NA1)<br>803(H1HN1F1NA1) | 5.39 | Le$^X$, sLe$^X$<br>α2,6-Sia |
| | | 2 | | | 1298.08 | | | | data not available | | 4.92 | xMS2 |
| | pk.4-12 | 1 | 61.32 | 60.35-62.08 | 981.70<br>1471.94 | 981.71<br>1472.05 | $M+3H^+$<br>$M+2H^+$ | H2HN3F3NA2C-PA | 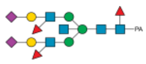 | 366(H1HN1)<br>657(H1HN1NA1)<br>803(H1HN1F1NA1)<br>1339(H3HN3F1-PA) | 17.20 | sLe$^X$, sLe$^X$<br>(identical to pk.5-10-1) |
| | pk.4-13 | 1 | 62.96 | 62.29-63.40 | 933.18<br>1399.08 | 933.02<br>1399.03 | $M+3H^+$<br>$M+2H^+$ | H2HN3F2NA2C-PA | 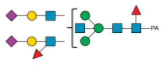 | 366(H1HN1)<br>657(H1HN1NA1)<br>803(H1HN1F1NA1) | 9.04 | sLe$^X$<br>α2,6-Sia<br>(identical to pk.5-11-1) |
| | pk.4-14 | 1 | 69.47 | 68.68-70.42 | 1103.98 | 1103.42 | $M+3H^+$ | H3HN4F3NA2C-PA | 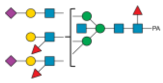 | 366(H1HN1)<br>512(H1HN1F1)<br>657(H1HN1NA1)<br>803(H1HN1F1NA1)<br>1542(H3HN4F1-PA) | 7.41 | Le$^X$, sLe$^X$<br>α2,6-Sia |
| fr.5 | pk.5-1 | 1 | 37.15 | 36.60-37.57 | 768.22<br>1151.49 | 767.95<br>1151.43 | $M+3H^+$<br>$M+2H^+$ | H2HN2NA2C-PA | 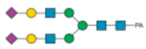 | 366(H1HN1)<br>657(H1HN1NA1)<br>1192(H3HN3-PA) | 3.15 | α2,6-Sia, α2,6-Sia artifact (epimer of pk.5-4-1) |

Table S1A Continued.

| Fr. No. (DEAE) | Peak. No. (ODS) | Full MS No. | Elusion time max (min) | Elusion time range (min) | Observed parent ion (*m/z* value) | Calculated (*m/z* value) | Estimated adduct | Estimated composition[a), c)] | Deduced glycan structure[b)] | Characteristic fragments[a)] | Relative amounts[d)] | Notes[f), g)] |
|---|---|---|---|---|---|---|---|---|---|---|---|---|
| | pk.5-2 | 1 | 44.11 | 43.61-44.72 | 816.61<br>1224.87 | 816.64<br>1224.46 | M+3H$^+$<br>M+2H$^+$ | H2HN2F1NA2C-PA | 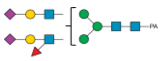 | 366(H1HN1)<br>512(H1HN1F1)<br>657(H1HN1NA1)<br>803(H1HN1F1NA1)<br>1192(H3HN3-PA) | 4.00 | sLe$^X$<br>α2,6-Sia |
| | pk.5-3 | 1 | 46.97 | 46.60-47.64 | 1285.77 | 1285.46 | M+2H$^+$ | H1HN3F3NA1(SO3)1C-PA | 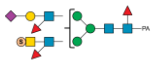 | 407(HN2)<br>553(HN2F1)<br>657(H1HN1NA1)<br>1542(H3HN4F1-PA) | 5.52 | sLe$^X$,<br>LacdiNAc with Fuc and SO3<br>(identical to pk.6-8-1) |
| | pk.5-4 | 1 | 48.59 | 47.85-49.24 | 768.20<br>1151.85 | 767.95<br>1151.43 | M+3H$^+$<br>M+2H$^+$ | H2HN2NA2C-PA | 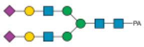 | 366(H1HN1)<br>657(H1HN1NA1)<br>1192(H3HN3-PA) | 52.39 | Standard A<br>α2,6-Sia, α2,6-Sia<br>(identical to pk.6-9-1) |
| | pk.5-5 | 1 | 50.13 | 49.44-50.90 | 768.16<br>1151.53 | 767.95<br>1151.43 | M+3H$^+$<br>M+2H$^+$ | H2HN2NA2C-PA | 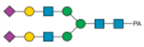 | 366(H1HN1)<br>657(H1HN1NA1)<br>1192(H3HN3-PA) | 4.66 | α2,3-Sia, α2,6-Sia |
| | pk.5-6 | 1 | 52.65 | 51.60-53.40 | 914.06<br>1370.86 | 914.01<br>1370.51 | M+3H$^+$<br>M+2H$^+$ | H2HN2F3NA2C-PA | 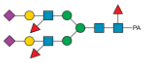 | 366(H1HN1)<br>657(H1HN1NA1)<br>803(H1HN1F1NA1)<br>1135(F1C-PA) | 7.67 | sLe$^X$<br>(identical to pk.4-10-1) |
| | | 2 | | | 1318.10 | 1318.00 | M+2H$^+$ | H1HN3F2NA2C-PA | 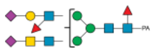 | 407(HN2)<br>698(HN2NA1)<br>803(H1HN1F1NA1) | 1.63 | sLe$^X$<br>sLacdiNAc with α2,6-Sia |
| | pk.5-7 | 1 | 54.39 | 53.68-55.07 | 865.42<br>1297.66 | 865.33<br>1297.49 | M+3H$^+$<br>M+2H$^+$ | H2HN2F2NA2C-PA | 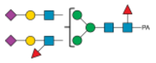 | 366(H1HN1)<br>657(H1HN1NA1)<br>803(H1HN1F1NA1)<br>1339(H3HN3F1-PA) | 13.76 | sLe$^X$<br>α2,6-Sia |
| | pk.5-8 | 1 | 55.78 | 55.28-56.67 | 889.67<br>1334.49 | 889.67<br>1333.99 | M+3H$^+$<br>M+2H$^+$ | H3HN3NA2C-PA | 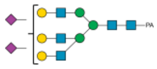 | 366(H1HN1)<br>657(H1HN1NA1)<br>1192(H3HN3-PA) | 10.18 | α2,6-Sia, α2,6-Sia |
| | pk.5-9 | 1 | 58.18 | 57.22-59.10 | 816.88<br>1224.55 | 816.64<br>1224.46 | M+3H$^+$<br>M+2H$^+$ | H2HN2F1NA2C-PA | 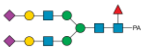 | 366(H1HN)<br>657(H1HN1NA1)<br>973(H2HN2F1-PA)<br>1135(F1C-PA) | 12.32 | Standard B<br>α2,6-Sia, α2,6-Sia |
| | pk.5-10 | 1 | 60.99 | 59.86-61.60 | 981.80<br>1472.44 | 981.71<br>1472.05 | M+3H$^+$<br>M+2H$^+$ | H2HN3F3NA2C-PA | 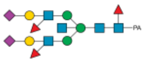 | 657(H1HN1NA1)<br>803(H1HN1F1NA1)<br>1339(H3HN3F1-PA) | 6.24 | sLe$^X$, sLe$^X$<br>(identical to pk.4-12-1) |
| | pk.5-11 | 1 | 62.65 | 61.74-63.68 | 933.03<br>1399.49 | 933.02<br>1399.03 | M+3H$^+$<br>M+2H$^+$ | H2HN3F2NA2C-PA | 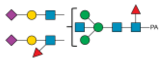 | 366(H1HN1)<br>657(H1HN1NA1)<br>803(H1HN1F1NA1)<br>1339(H3HN3F1-PA) | 23.04 | sLe$^X$,<br>α2,6-Sia<br>(identical to pk.4-13-1) |
| | | 2 | | | 884.58 | 884.33 | M+2H$^+$ | H2HN3F1NA2C-PA | 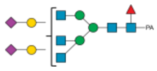 | 366(H1HN1)<br>657(H1HN1NA1)<br>1339(H3HN3F1-PA) | 1.63 | α2,6-Sia, α2,6-Sia |
| | pk.5-12 | 1 | 67.40 | 67.10-67.99 | 1224.77 | 1224.46 | M+2H$^+$ | H2HN2F1NA2C-PA | 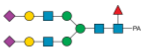 | 657(H1HN1NA1)<br>1135(F1C-PA)<br>1339(H3HN3F1-PA) | 2.76 | α2,3-Sia, α2,3-Sia |
| | pk.5-13 | 1 | 73.11 | 72.36-73.75 | 884.59<br>1326.11 | 884.33<br>1326.00 | M+3H$^+$<br>M+2H$^+$ | H2HN3F1NA2C-PA | 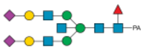 | 366(H1HN1)<br>657(H1HN1NA1)<br>1339(H3HN3F1-PA)<br>1542(H3HN4F1-PA) | 6.27 | Standard D<br>α2,6-Sia, α2,6-Sia |
| | pk.5-14 | 1 | 76.07 | 75.56-76.67 | 884.90<br>1325.92 | 884.33<br>1326.00 | M+3H$^+$<br>M+2H$^+$ | H2HN3F1NA2C-PA | 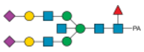 | 657(H1HN1NA1)<br>1542(H3HN4F1-PA) | 3.15 | α2,3-Sia, α2,6-Sia |
| | | | | | | | | | | | | |
| fr.6 | pk.6-1 | 1 | 12.95 | 12.52-13.28 | 859.07 | 859.29 | M+2H$^+$ | H4(HPO3)C-PA | 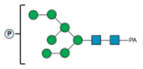 | 243(H1(HPO3)1)<br>405(H2(HPO3)1)<br>1151(H4HN2-PA) | 1.93 | HPO3 |
| | pk.6-2 | 1 | 13.95 | 13.35-14.84 | 1101.94 | | | data not available | | | 6.10 | (artifact) |
| | | 2 | | | 1247.44 | | | data not available | | | 2.21 | (artifact) |

Table S1A Continued.

| Fr. No. (DEAE) | Peak. No. (ODS) | Full MS No. | Elution time max (min) | Elution time range (min) | Observed parent ion (*m/z* value) | Calculated (*m/z* value) | Estimated adduct | Estimated composition[a), c)] | Deduced glycan structure[b)] | Characteristic fragments[a)] | Relative amounts[d)] | Notes[f), g)] |
|---|---|---|---|---|---|---|---|---|---|---|---|---|
| | pk.6-3 | 1 | 15.81 | 15.08-16.53 | 1021.07 | | | data not available | | | 2.31 | (artifact) |
| | pk.6-4 | 1 | 18.14 | 17.91-18.60 | 1183.13 | | | data not available | | | 1.20 | xMS2 |
| | pk.6-5 | 1 | 19.12 | 18.81-19.43 | 858.96 | 859.29 | M+2H+ | H4(HPO3)C-PA | 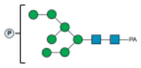 | 243(H1(HPO3)1)<br>405(H2(HPO3)1)<br>1151(H4HN2-PA) | 0.62 | HPO3 |
| | pk.6-6 | 1 | 36.83 | 36.34-37.69 | 1212.24 | | | data not available | | | 1.76 | xMS2 |
| | pk.6-7 | 1 | 38.83 | 38.04-39.00 | 1285.63 | | | data not available | | | 0.71 | xMS2 |
| | pk.6-8 | 1 | 47.09 | 46.32-47.78 | 857.34<br>1285.25 | 857.31<br>1285.46 | $M+3H^+$<br>$M+2H^+$ | H1HN3F3NA1(SO3)1C-PA | 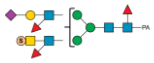 | 553(HN2F1)<br>657(H1HN1NA1)<br>803(H1HN1F1NA1)<br>1135(F1C-PA) | 12.75 | sLe$^x$,<br>LacdiNAc with Fuc and SO3<br>(identical to pk.5-3-1) |
| | pk.6-9 | 1 | 48.87 | 48.06-49.79 | 1152.03 | 1151.43 | $M+2H^+$ | H2HN2NA2C-PA | 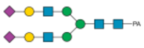 | 366(H1HN1)<br>657(H1HN1NA1)<br>1192(H3HN3-PA) | 3.06 | Standard A<br>α2,6-Sia, α2,6-Sia<br>(identical to pk.5-4-1) |
| | pk.6-10 | 1 | 50.91 | 50.27-51.21 | 808.74<br>1212.90 | 808.62<br>1212.43 | $M+3H^+$<br>$M+2H^+$ | H1HN3F2NA1(SO3)1C-PA | 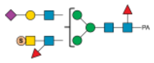 | 407(HN2)<br>553(HN2F1)<br>657(H1HN1NA1)<br>1339(H3HN3F1-PA) | 2.62 | LacdiNAc with Fuc and SO3 |
| | pk.6-11 | 1 | 51.72 | 51.28-52.09 | 1212.71 | | | data not available | | | 1.76 | xMS2 |
| | | | | | | | | | | | | |
| fr.7 | pk.7-1 | 1 | 14.43 | 14.07-14.89 | 777.96 | 778.26 | $M+2H^+$ | H3(HPO3)C-PA | 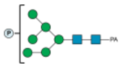 | 243(H1(HPO3)1)<br>405(H2(HPO3)1)<br>1151(H4HN2-PA) | 2.95 | HPO3 |
| | pk.7-2 | 1 | 40.67 | 40.29-41.32 | 1180.34 | | | data not available | | | 1.25 | xMS2 |
| | pk.7-3 | 1 | 48.41 | 47.91-49.28 | 1192.27 | | | data not available | | | 1.38 | xMS2 |
| | pk.7-4 | 1 | 55.43 | 54.98-56.21 | 1118.79 | | | data not available | | | 1.48 | xMS2 |
| | | | | | | | | | | | | |
| fr.8 | pk.8-1 | 1 | 14.44 | 14.14-14.83 | 778.14 | 778.26 | $M+2H^+$ | H3(HPO3)C-PA | 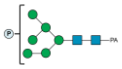 | 243(H1(HPO3)1)<br>405(H2(HPO3)1)<br>1151(H4HN2-PA) | 1.65 | HPO3 |
| | pk.8-2 | 1 | 18.97 | 18.50-19.68 | | | | data not available | | | 0.24 | xMS2 |
| | pk.8-3 | 1 | 22.25 | 21.34-22.59 | 777.96 | | | data not available | | | 0.43 | xMS2 |
| | pk.8-4 | 1 | 33.50 | 33.06-34.16 | 1127.29 | | | data not available | | | 0.91 | xMS2 |
| | | 2 | | | 1200.23 | | | data not available | | | 0.52 | xMS2 |

Table S1A Continued.

| Fr. No. (DEAE) | Peak. No. (ODS) | Full MS No. | Elusion time max (min) | Elusion time range (min) | Observed parent ion (*m/z* value) | Calculated (*m/z* value) | Estimated adduct | Estimated composition[a), c)] | Deduced glycan structure[b)] | Characteristic fragments[a)] | Relative amounts[d)] | Notes[f), g)] |
|---|---|---|---|---|---|---|---|---|---|---|---|---|
| | pk.8-5 | 1 | 42.95 | 41.63-43.76 | 800.77<br>1200.21 | 800.60<br>1200.40 | $M+3H^+$<br>$M+2H^+$ | HN4F3(SO3)2C-PA | 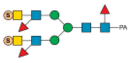 | 407(HN2)<br>553(HN2F1)<br>633(HN2F1(SO3)1) | 14.87 | LacdiNAc with Fuc and SO3, LacdiNAc with Fuc and SO3 |
| | pk.8-6 | 1 | 47.52 | 46.99-47.96 | 1181.41 | 1181.44 | $M+3H^+$ | H3HN3F4NA3C-PA | 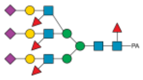 | 366(H1HN1)<br>512(H1HN1F1)<br>657(H1HN1NA1)<br>1339(H3HN3F1-PA) | 2.18 | $sLe^X$, $sLe^X$, $sLe^X$ |
| | pk.8-7 | 1 | 49.36 | 49.00-51.14 | | | | | data not available | | 5.69 | xMS2 |
| | pk.8-8 | 1 | 52.47 | 51.83-53.20 | 1132.62 | 1132.75 | $M+3H^+$ | H3HN3F3NA3C-PA | 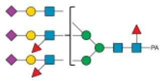 | 803(H1HN1F1NA1) | 2.02 | $sLe^X$, $sLe^X$<br>α2,6-Sia |
| | pk.8-9 | 1 | 54.08 | 53.43-55.16 | 1200.85 | | | | data not available | | 5.02 | xMS2 |
| | pk.8-10 | 1 | 56.08 | 55.17-55.99 | 1132.88 | 1132.75 | $M+3H^+$ | H3HN3F3NA3C-PA | 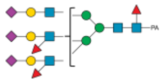 | 366(H1HN1)<br>512(H1HN1F1)<br>657(H1HN1NA1)<br>803(H1HN1F1NA1) | 1.46 | $sLe^X$, $sLe^X$<br>α2,6-Sia |
| | pk.8-11 | 1 | 57.53 | 56.93-58.30 | 986.60<br>1479.46 | 986.70<br>1479.54 | $M+3H^+$<br>$M+2H^+$ | H3HN3NA3C-PA | 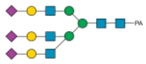 | 366(H1HN1)<br>657(H1HN1NA1)<br>1192(H3HN3-PA) | 4.67 | α2,6-Sia, α2,6-Sia, α2,6-Sia |
| | pk.8-12 | 1 | 60.01 | 58.79-61.13 | 1132.72 | 1132.75 | $M+3H^+$ | H3HN3F3NA3C-PA | 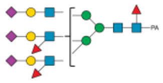 | 366(H1HN1)<br>657(H1HN1NA1)<br>803(H1HN1F1NA1) | 3.07 | $sLe^X$, $sLe^X$<br>α2,6-Sia |
| | pk.8-13 | 1 | 61.89 | 61.34-62.99 | 986.65<br>1480.22 | 986.70<br>1479.54 | $M+3H^+$<br>$M+2H^+$ | H3HN3NA3C-PA | 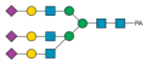 | 366(H1HN1)<br>657(H1HN1NA1)<br>1192(H3HN3-PA) | 2.87 | α2,3-Sia, α2,6-Sia, α2,6-Sia |
| | pk.8-14 | 1 | 69.86 | 69.72-70.62 | 1249.78 | 1249.13 | $M+3H^+$ | H3HN4F4NA3C-PA | 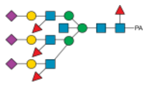 | 657(H1HN1NA1)<br>803(H1HN1F1NA1) | 1.32 | $sLe^X$, $sLe^X$, $sLe^X$ |
| | pk.8-15 | 1 | 71.85 | 70.99-72.92 | 1200.60 | 1200.45 | $M+3H^+$ | H3HN4F3NA3C-PA | 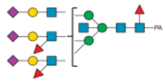 | 366(H1HN1)<br>657(H1HN1NA1)<br>803(H1HN1F1NA1) | 3.84 | $sLe^X$, $sLe^X$<br>α2,6-Sia |
| | pk.8-16 | 1 | 80.17 | 79.83-80.80 | 1151.90 | | | | data not available | | 0.77 | xMS2 |
| | pk.8-17 | 1 | 82.04 | 81.91-82.54 | 1152.40 | | | | data not available | | 0.59 | xMS2 |
