# Supplementary material for: Structural analysis of N-glycans in chicken trachea and lung reveals potential receptors of chicken influenza viruses
Source: Sci Rep. 2022 Feb 8;12:2081. doi: 10.1038/s41598-022-05961-x (PMC8827061; doi:10.1038/s41598-022-05961-x)
Supplement: Supplementary file 4 — Supplementary Table S2. [file 41598_2022_5961_MOESM4_ESM.pdf]

Table S1B. MS and MS/MS data for PA-*N*-glycans from chicken lung on LC-MS

[a] H, hexose; HN, *N*-acetylhexosamine; F, fucose (or deoxyhexose); GN, *N*-acetylglucosamine; M, mannose; NA, *N*-acetylneuraminic acid; SO3, sulfate group; HPO3, phosphate group; C, trimannosyl core; PA, 2-aminopyridine.

[b] Monosaccharide symbols for deduced glycan structures were as follows:

: Man : Gal : Glc : GlcNAc : GalNAc : Fuc : NeuAc : sulfate group : phosphate group

[c] Most of the detectable fluorescence peaks eluted in 10–87 min were numbered, but not all peaks were derived from PA-*N*-glycans. The compositions of some peaks could not be determined by MS and MS/MS analysis due to insufficent signals; these cases are indicated as "data not available".

[d] Individual peaks detected by fluorescence sometimes included more than two kinds of PA-glycans with different mass values. In such cases, the proportions were estimated using the ratios of integrated ion intensities for each *m/z* value detected at the corresponding elution time.

[e] Amounts of glycans relative to the most abundant glycan (pk.1-28-1), for which the amount was defined as 100.

[f] xMS, MS data not available; xMS2, MS/MS data not available.

[g] Linkages of sialic acids (α2,3, α2,6) were deduced based on the elution positions and the results of SALSA and/or SALSA/permethylation.

Fr. No. (DEAE)	Peak. No. (ODS)	Full MS No.	Elution time max (min)	Elusion time range (min)	Observed parent ion (*m/z* value)	Calculated (*m/z* value)	Estimated adduct	Estimated composition[a), c)]	Deduced glycan structure[b)]	Characteristic fragments[a)]	Relative amounts[d)]	Notes[f), g)]
fr.1	pk.1-1	1	14.92	14.59-15.15	900.00	900.33	M+2H+	H5C-PA (M8GN2-PA)	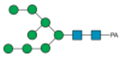	325(H2)<br>1151(H4HN2-PA)	1.42	artifact (Schiff base by incomplete reduction of pk. 1-6-1)
	pk.1-2	1	15.51	15.21-15.70	818.78	819.31	M+2H+	H4C-PA (M7GN2-PA)	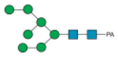	325(H2)<br>1151(H4HN2-PA)	0.29	artifact (epimer of pk.1-7-1)
		2			980.15	981.36	M+2H+	H6C-PA (M9GN2-PA)	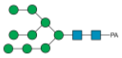	325(H2)<br>1151(H4HN2-PA)	0.31	artifact (Schiff base by incomplete reduction of pk.1-8-1)
	pk.1-3	1	16.04	15.84-16.46	980.87	981.36	M+2H+	H6C-PA (M9GN2-PA)	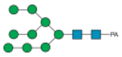	325(H2)<br>1151(H4HN2-PA)	1.02	artifact (epimer of pk.1-8-1)
	pk.1-4	1	18.05	17.70-18.46	737.83	738.28	M+2H+	H3C-PA (M6GN2-PA)	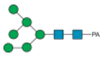	325(H2)<br>1151(H4HN2-PA)	1.07	artifact (epimer of pk.1-11-1)
		2			923.95			data not available			0.18	(artifact)
	pk.1-5	1	21.55	21.02-22.20	656.95<br>1313.51	657.25<br>1313.50	M+2H+<br>M+H+	H2C-PA (M5GN2-PA)	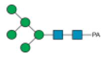	325(H2)<br>1151(H4HN2-PA)	1.28	artifact (epimer of pk.1-14-1)
	pk.1-6	1	23.59	22.75-24.07	900.10	900.33	M+2H+	H5C-PA (M8GN2-PA)	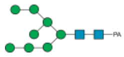	325(H2)<br>1151(H4HN2-PA)	42.95	
	pk.1-7	1	24.77	24.14-25.38	818.87	819.31	M+2H+	H4C-PA (M7GN2-PA)	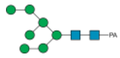	325(H2)<br>1151(H4HN2-PA)	23.81	
	pk.1-8	1	26.02	25.45-26.76	981.17	981.36	M+2H+	H6C-PA (M9GN2-PA)	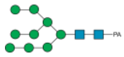	325(H2)<br>1151(H4HN2-PA)	43.67	
	pk.1-9	1	27.21	26.90-27.66	900.32	900.33	M+2H+	H5C-PA (M8GN2-PA)	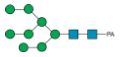	325(H2)<br>1151(H4HN2-PA)	3.80	
	pk.1-10	1	28.22	27.80-28.70	818.89	819.31	M+2H+	H4C-PA (M7GN2-PA)	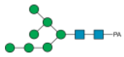	325(H2)<br>1151(H4HN2-PA)	8.92	
		2			737.85	738.28	M+2H+	H3C-PA (M6GN2-PA)	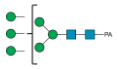	325(H2)<br>1151(H4HN2-PA)	3.33	
	pk.1-11	1	29.30	28.84-30.29	738.01<br>1475.57	738.28<br>1475.55	M+2H+<br>M+H+	H3C-PA (M6GN2-PA)	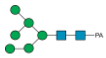	325(H2)<br>1151(H4HN2-PA)	49.30	
	pk.1-12	1	31.22	30.50-31.60	997.19			data not available			3.16	(artifact)
		2			860.50	860.33	M+2H+	H2HN2C-PA	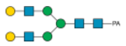	366(H1HN1)	1.28	artifact (epimer of pk.1-22-1)

Table S1B Continued.

Fr. No. (DEAE)	Peak. No. (ODS)	Full MS No.	Elusion time max (min)	Elusion time range (min)	Observed parent ion (*m/z* value)	Calculated (*m/z* value)	Estimated adduct	Estimated composition[a), c)]	Deduced glycan structure[b)]	Characteristic fragments[a)]	Relative amounts[d)]	Notes[f), g)]
		3			1143.51	1143.41	M+2H$^+$	H8C-PA (Glc1M8GN2-PA)	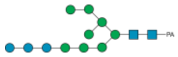	1151(H4HN2-PA)<br>1314(H5HN2-PA)	1.36	
		4			1298.99	1298.49	M+2H$^+$	H4HN4F1C-PA	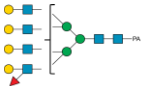	366(H1HN1)<br>512(H1HN1F1)	1.10	Le^x
	pk.1-13	1	32.05	31.67-32.43	1061.96	1062.39	M+2H$^+$	H7C-PA (Glc1M9GN2-PA)	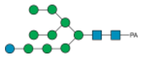	325(H2)<br>1151(H4HN2-PA)	9.83	
	pk.1-14	1	33.29	32.50-34.30	657.00<br>1313.53	657.25<br>1313.50	M+2H$^+$<br>M+H$^+$	H2C-PA (M5GN2-PA)	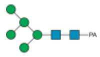	325(H2)<br>1151(H4HN2-PA)	96.71	
	pk.1-15	1	35.33	34.65-36.38	695.71<br>1043.50	695.60<br>1042.90	M+3H$^+$<br>M+2H$^+$	H3HN3C-PA	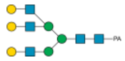	366(H1HN1)	26.83	
		2			840.42	839.82	M+2H$^+$	H3HN1C-PA	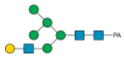	366(H1HN)<br>1314(H5HN2-PA)	19.28	
		3			759.39	758.79	M+2H$^+$	H2HN1C-PA	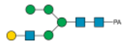	366(H1HN1)<br>1151(H4HN2-PA)	7.19	
	pk.1-16	1	36.91	36.51-37.55	763.38<br>1144.85	763.29<br>1144.44	M+3H$^+$<br>M+2H$^+$	H3HN4C-PA	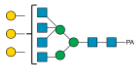	366(H1HN1)<br>1192(H3HN3-PA)	8.42	
		2			738.06	738.28	M+2H$^+$	H3C-PA (M6GN2-PA)	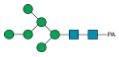	325(H2)<br>1151(H4HN2-PA)	1.40	
		3			933.30	933.36	M+2H$^+$	H2HN2F1C-PA	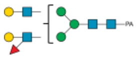	366(H1HN1)<br>512(H1HN1F1)<br>1192(H3HN3-PA)	1.14	Le^x
	pk.1-17	1	38.29	37.69-38.93	817.34<br>1225.60	817.31<br>1225.46	M+3H$^+$<br>M+2H$^+$	H4HN4C-PA	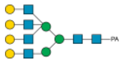	366(H1HN1)<br>1192(H3HN3-PA)	30.68	
	pk.1-18	1	39.21	39.00-39.70	860.84	860.33	M+2H$^+$	H2HN2C-PA	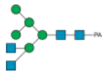	1151(H4HN2-PA)<br>1314(H5HN2-PA)	2.87	
		2			698.35	698.28	M+2H$^+$	HN2C-PA	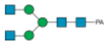	204(HN1)<br>1192(H3HN3-PA)	1.07	Standard I
		3			792.93<br>1189.37	792.97<br>1188.96	M+3H$^+$<br>M+2H$^+$	H3HN3F2C-PA	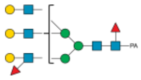	366(H1HN1)<br>512(H1HN1F1)<br>1339(H3HN3F1-PA)	1.03	Le^x
	pk.1-19	1	40.24	39.83-40.94	817.50<br>1226.53	817.31<br>1225.46	M+3H$^+$<br>M+2H$^+$	H4HN4C-PA	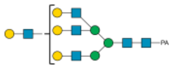	366(H1HN1)	3.86	LacNAc repeat
		2			953.73	953.87	M+2H$^+$	H1HN3F1C-PA	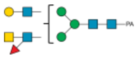	366(H1HN1)<br>407(HN2)<br>553(HN2F1)	1.95	LacdiNAc with Fuc
	pk.1-20	1	42.00	41.01-42.71	933.51	933.36	M+2H$^+$	H2HN2F1C-PA	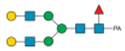	366(H1HN1)<br>1339(H3HN3F1-PA)	10.72	artifact (epimer of pk.1-28-1)
		2			779.92	779.31	M+2H$^+$	H1HN2C-PA	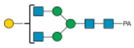	366(H1HN1)<br>1192(H3HN3-PA)	7.00	

Table S1B Continued.

Fr. No. (DEAE)	Peak. No. (ODS)	Full MS No.	Elusion time max (min)	Elusion time range (min)	Observed parent ion (*m/z* value)	Calculated (*m/z* value)	Estimated adduct	Estimated composition[a), c)]	Deduced glycan structure[b)]	Characteristic fragments[a)]	Relative amounts[d)]	Notes[f), g)]
		3			941.35	941.36	$M+2H^+$	H3HN2C-PA	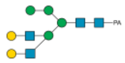	366(H1HN1)<br>1151(H4HN2-PA)<br>1192(H3HN3-PA)<br>1314(H5HN2-PA)	4.10	
	pk.1-21	1	43.15	42.76-43.44	872.60	872.85	$M+2H^+$	HN3F1C-PA	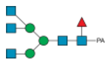	973(H2HN2F1-PA)<br>1542(H3HN4F1-PA)	1.38	
		2			1034.96	1034.90	$M+2H^+$	H2HN3F1C-PA	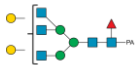	366(H1HN1)<br>1501(H1HN1F1C-PA)<br>1704(H1HN2F1C-PA)	0.41	
		3			941.62	941.36	$M+2H^+$	H3HN2C-PA	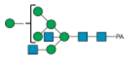	366(H1HN1)<br>1151(H4HN2-PA)<br>1314(H5HN2-PA)<br>1679(H3HN1C-PA)	0.67	
	pk.1-22	1	44.06	43.45-44.54	860.82	860.33	$M+2H^+$	H2HN2C-PA	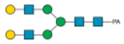	366(H1HN1)<br>1192(H3HN3-PA)	32.74	Standard E
		2			677.68	677.77	$M+2H^+$	H1HN1C-PA	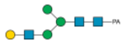	366(H1HN1)<br>989(H3HN2-PA)	2.69	
	pk.1-23	1	45.41	44.61-46.40	744.80<br>1116.52	744.29<br>1115.93	$M+3H^+$<br>$M+2H^+$	H3HN3F1C-PA	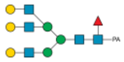	366(H1HN1)<br>1135(F1C-PA)<br>1339(H3HN3F1-PA)	25.53	
		2			973.45	973.40	$M+H^+$	H2HN2F1-PA	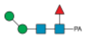	446(HN1F1-PA)	7.20	
		3			912.92	912.85	$M+2H^+$	H3HN1F1C-PA	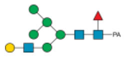	366(H1HN1)<br>1135(F1C-PA)<br>1460(H2F1C-PA)	4.54	
		4			1135.49	1135.45	$M+2H^+$	F1C-PA	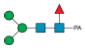	446(HN1F1-PA)	6.21	
		5			1006.69	1006.39	$M+2H^+$	H2HN2F2C-PA	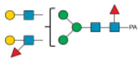	366(H1HN1)<br>512(H1HN1F1)<br>1339(H3HN3F1-PA)	1.03	Le^X
	pk.1-24	1	47.04	46.47-47.79	866.15<br>1298.63	866.00<br>1298.49	$M+3H^+$<br>$M+2H^+$	H4HN4F1C-PA	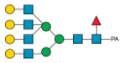	366(H1HN1)<br>1339(H3HN3F1-PA)<br>1542(H3HN4F1-PA)	17.25	
		2			832.34	831.82	$M+2H^+$	H2HN1F1C-PA	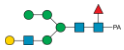	366(H1HN1)<br>973(H2HN2F1-PA)<br>1135(F1C-PA)<br>1297(H1F1C-PA)	5.96	
		3			750.71	750.80	$M+2H^+$	H1HN1F1C-PA	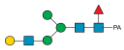	366(H1HN1)<br>973(H2HN2F1-PA)<br>1135(F1C-PA)	3.61	
	pk.1-25	1	48.08	47.84-48.50	941.46	941.36	$M+2H^+$	H3HN2C-PA	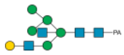	366(H1HN1)<br>1151(H4HN2-PA)<br>1192(H3HN3-PA)	5.18	
		2			1181.16	1180.96	$M+2H^+$	H2HN3F3C-PA	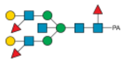	366(H1HN1)<br>512(H1HN1F1)<br>1501(H1HN1F1C-PA)	1.19	Le^X
	pk.1-26	1	50.39	49.65-51.04	771.17	771.31	$M+2H^+$	HN2F1C-PA	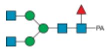	973(H2HN2F1-PA)<br>1135(F1C-PA)<br>1339(H3HN3F1-PA)	6.95	Standard J
		2			866.21<br>1298.52	866.00<br>1298.49	$M+3H^+$<br>$M+2H^+$	H4HN4F1C-PA	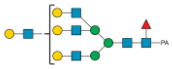	366(H1HN1)<br>731(H2HN2)<br>1339(H3HN3F1-PA)	3.68	LacNAc repeat

Table S1B Continued.

Fr. No. (DEAE)	Peak. No. (ODS)	Full MS No.	Elusion time max (min)	Elusion time range (min)	Observed parent ion (*m/z* value)	Calculated (*m/z* value)	Estimated adduct	Estimated composition[a), c)]	Deduced glycan structure[b)]	Characteristic fragments[a)]	Relative amounts[d)]	Notes[f), g)]
		3			1027.81	1026.90	$M+2H^+$	H1HN3F2C-PA	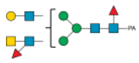	366(H1HN1)<br>407(HN2)<br>1501(H1HN1F1C-PA)	1.11	LacdiNAc with Fuc
	pk.1-27	1	52.47	51.59-53.32	852.32	852.33	$M+2H^+$	H1HN2F1C-PA	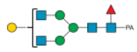	366(H1HN1)<br>973(H2HN2F1-PA)<br>1135(F1C-PA)<br>1339(H3HN3F1-PA)	20.52	
	pk.1-28	1	54.59	53.46-56.43	622.79<br>933.49	622.58<br>933.36	$M+3H^+$<br>$M+2H^+$	H2HN2F1C-PA	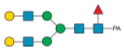	366(H1HN1)<br>973(H2HN2F1-PA)<br>1135(F1C-PA)	100.00	Standard F
		2			954.42	953.87	$M+2H^+$	H1HN3F1C-PA	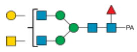	366(H1HN1)<br>407(HN2)<br>1339(H3HN3F1-PA)	4.28	LacdiNAc
	pk.1-29	1	57.21	56.64-57.68	881.51	880.85	$M+2H^+$	H1HN3C-PA	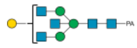	366(H1HN1)	4.10	
		2			987.94	987.71	$M+3H^+$	H5HN5F1C-PA	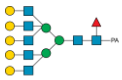	366(H1HN1)<br>1339(H3HN3F1-PA)	1.73	
	pk.1-30	1	58.20	57.75-59.06	881.48	880.85	$M+2H^+$	H1HN3C-PA	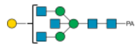	366(H1HN1)<br>1192(H3HN3-PA)	7.05	
		2			1218.08	1217.47	$M+2H^+$	H3HN4F1C-PA	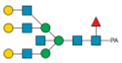	366(H1HN1)<br>1542(H3HN4F1-PA)	2.94	
		3			954.28	953.87	$M+2H^+$	H1HN3F1C-PA	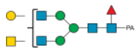	366(H1HN1)<br>407(HN2)<br>1339(H3HN3F1-PA)	0.68	LacdiNAc
	pk.1-31	1	59.81	59.13-60.10	933.49	933.36	$M+2H^+$	H2HN2F1C-PA	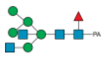	366(H1HN1)<br>973(H2HN2F1-PA)<br>1135(F1C-PA)	3.53	
	pk.1-32	1	60.85	60.30-61.62	641.63<br>961.81	641.58<br>961.87	$M+3H^+$<br>$M+2H^+$	H2HN3C-PA	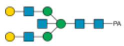	366(H1HN1)<br>1192(H3HN3-PA)	17.35	Standard G
	pk.1-33	1	63.11	62.45-63.69	744.40<br>1115.62	744.29<br>1115.93	$M+3H^+$<br>$M+2H^+$	H3HN3F1C-PA	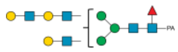	366(H1HN1)<br>731(H2HN2)<br>973(H2HN2F1-PA)<br>1135(F1C-PA)	4.24	LacNAc repeat
	pk.1-34	1	65.79	65.01-66.25	744.32<br>1116.47	744.29<br>1115.93	$M+3H^+$<br>$M+2H^+$	H3HN3F1C-PA	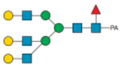	366(H1HN1)<br>973(H2HN2F1-PA)<br>1135(F1C-PA)<br>1339(H3HN3F1-PA)	2.21	
		2			933.51	933.36	$M+2H^+$	H2HN2F1C-PA	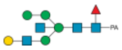	366(H1HN1)<br>1339(H3HN3F1-PA)	0.60	
	pk.1-35	1	69.27	68.33-70.06	582.40<br>873.35	582.23<br>872.85	$M+3H^+$<br>$M+2H^+$	HN3F1C-PA	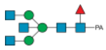	1339(H3HN3F1-PA)	5.65	Standard L
	pk.1-36	1	71.44	70.54-72.06	636.35<br>954.31	636.25<br>953.87	$M+3H^+$<br>$M+2H^+$	H1HN3F1C-PA	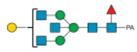	366(H1HN1)<br>973(H2HN2F1-PA)<br>1135(F1C-PA)<br>1339(H3HN3F1-PA)	11.86	
	pk.1-37	1	72.68	72.13-73.37	636.21<br>954.41	636.25<br>953.87	$M+3H^+$<br>$M+2H^+$	H1HN3F1C-PA	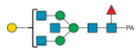	366(H1HN1)<br>973(H2HN2F1-PA)<br>1135(F1C-PA)<br>1339(H3HN3F1-PA)	13.09	
	pk.1-38	1	74.36	73.51-75.24	690.85<br>1035.11	690.27<br>1034.90	$M+3H^+$<br>$M+2H^+$	H2HN3F1C-PA	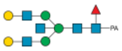	366(H1HN1)<br>1135(F1C-PA)<br>1339(H3HN3F1-PA)	78.27	Standard H

Table S1B Continued.

Fr. No. (DEAE)	Peak. No. (ODS)	Full MS No.	Elusion time max (min)	Elusion time range (min)	Observed parent ion (*m/z* value)	Calculated (*m/z* value)	Estimated adduct	Estimated composition[a), c)]	Deduced glycan structure[b)]	Characteristic fragments[a)]	Relative amounts[d)]		Notes[f), g)]
	pk.1-39	1	76.74	76.07-77.39	744.36<br>1116.38	744.29<br>1115.93	$M+3H^+$<br>$M+2H^+$	H3HN3F1C-PA	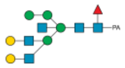	366(H1HN1)<br>1339(H3HN3F1-PA)	1.00		
	pk.1-40	1	79.86	79.46-80.43	812.51<br>1217.84	811.98<br>1217.47	$M+3H^+$<br>$M+2H^+$	H3HN4F1C-PA	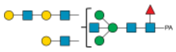	366(H1HN1)<br>731(H2HN2)<br>1339(H3HN3F1-PA)<br>1542(H3HN4F1-PA)	1.00		LacNAc repeat
	pk.1-41	1	85.62	85.13-86.31	812.29<br>1217.95	811.98<br>1217.47	$M+3H^+$<br>$M+2H^+$	H3HN4F1C-PA	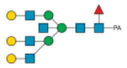	366(H1HN1)<br>1339(H3HN3F1-PA)<br>1542(H3HN4F1-PA)	3.15		
fr.3	pk.3-1	1	12.67	12.45-12.93	967.71			data not available			0.44		(artifact)
	pk.3-2	1	15.19	14.94-15.49	915.33			data not available			0.34		(artifact)
	pk.3-3	1	16.70	16.39-17.15	753.31	753.30	$M+H^+$	H1HN1NA1-PA	unknown		1.03		non-*N*-glycan
	pk.3-4	1	18.08	17.57-18.40	880.05	879.80	$M+2H^+$	H3HN1(HPO3)1C-PA	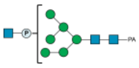	405(H2(HPO3)1)<br>1151(H4HN2-PA)	3.11		HPO3
	pk.3-5	1	19.31	18.95-19.71	1042.06	1041.86	$M+2H^+$	H5HN1(HPO3)1C-PA	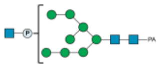	405(H2(HPO3)1)<br>1151(H4HN2-PA)	2.14		HPO3
	pk.3-6	1	20.22	19.78-20.61	960.63	960.83	$M+2H^+$	H4HN1(HPO3)1C-PA	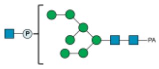	405(H2(HPO3)1)<br>1151(H4HN2-PA)	1.08		HPO3
	pk.3-7	1	22.41	22.06-22.75	1040.74			data not available			0.13		(artifact)
	pk.3-8	1	23.33	22.96-23.65	879.99	879.80	$M+2H^+$	H3HN1(HPO3)1C-PA	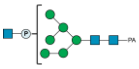	405(H2(HPO3)1)<br>1151(H4HN2-PA)	0.57		HPO3
	pk.3-9	1	33.29	32.64-33.82	670.89<br>1005.89	670.92<br>1005.88	$M+3H^+$<br>$M+2H^+$	H2HN2NA1C-PA	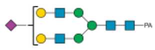	366(H1HN1)<br>657(H1HN1NA1)	1.55		α2,6-Sia<br>artifact (epimer of pk.3-17-1)
	pk.3-10	1	37.76	36.65-38.11	657.28<br>985.17	657.25<br>985.37	$M+3H^+$<br>$M+2H^+$	H3HN1NA1C-PA	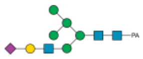	366(H1HN1)<br>657(H1HN1NA1)<br>1314(H5HN2-PA)	3.59		α2,6-Sia
		2			823.42	823.31	$M+2H^+$	H1HN1NA1C-PA	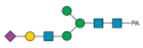	366(H1HN1)<br>657(H1HN1NA1)	0.73		α2,6-Sia
		3			1188.99	1188.45	$M+2H^+$	H3HN3NA1C-PA	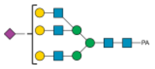	366(H1HN1)<br>657(H1HN1NA1)<br>1355(H1HN1C-PA)	0.85		α2,6-Sia
	pk.3-11	1	38.45	38.18-38.87	904.40	904.34	$M+2H^+$	H2HN1NA1C-PA	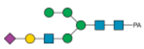	366(H1HN1)<br>657(H1HN1NA1)<br>1151(H4HN2-PA)	1.46		α2,6-Sia
	pk.3-12	1	40.00	39.66-40.22	792.73<br>1188.68	792.63<br>1188.45	$M+3H^+$<br>$M+2H^+$	H3HN3NA1C-PA	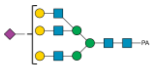	366(H1HN1)<br>657(H1HN1NA1)<br>1192(H3HN3-PA)	4.15		α2,3-Sia
	pk.3-13	1	41.60	40.80-41.91	657.09<br>985.33	657.25<br>985.37	$M+3H^+$<br>$M+2H^+$	H3HN1NA1C-PA	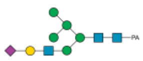	366(H1HN1)<br>657(H1HN1NA1)<br>1314(H5HN2-PA)	3.79		α2,3-Sia

Table S1B Continued.

Fr. No. (DEAE)	Peak. No. (ODS)	Full MS No.	Elusion time max (min)	Elusion time range (min)	Observed parent ion (*m/z* value)	Calculated (*m/z* value)	Estimated adduct	Estimated composition[a), c)]	Deduced glycan structure[b)]	Characteristic fragments[a)]	Relative amounts[d)]	Notes[f), g)]
		2			1188.38	1188.45	$M+2H^+$	H3HN3NA1C-PA	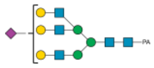	366(H1HN1)<br>657(H1HN1NA1)<br>1355(H1HN1C-PA)	0.74	α2,3-Sia
	pk.3-14	1	42.34	41.98-42.53	914.35<br>1371.63	914.34<br>1137.01	$M+3H^+$<br>$M+2H^+$	H4HN4NA1C-PA	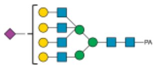	366(H1HN1)<br>657(H1HN1NA1)<br>1355(H1HN1C-PA)	1.57	α2,3-Sia
		2			792.60<br>1188.89	792.63<br>1188.45	$M+3H^+$<br>$M+2H^+$	H3HN3NA1C-PA	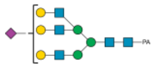	366(H1HN1)<br>657(H1HN1NA1)<br>1192(H3HN3-PA)	1.06	α2,3-Sia
		3			904.42	904.34	$M+2H^+$	H2HN1NA1C-PA	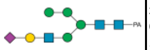	366(H1HN1)<br>657(H1HN1NA1)<br>1151(H4HN2-PA)	0.21	α2,3-Sia
	pk.3-15	1	42.88	42.67-43.36	904.64	904.34	$M+2H^+$	H2HN1NA1C-PA	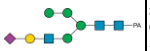	366(H1HN1)<br>657(H1HN1NA1)<br>1151(H4HN2-PA)	2.02	α2,3-Sia
		2			924.92	924.85	$M+2H^+$	H1HN2NA1C-PA	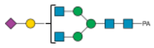	366(H1HN1)<br>657(H1HN1NA1)<br>1192(H3HN3-PA)	1.55	α2,6-Sia
	pk.3-16	1	43.87	43.50-44.33	914.48	914.34	$M+3H^+$	H4HN4NA1C-PA	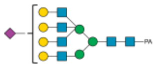	366(H1HN1)<br>657(H1HN1NA1)	1.43	α2,3-Sia
		2			1026.42	1026.39	$M+2H^+$	H1HN3NA1C-PA	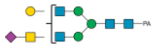	366(H1HN1)<br>407(HN2)<br>698(HN2NA1)<br>1192(H3HN3-PA)	1.58	sLacdiNAc with α2,6-Sia
	pk.3-17	1	45.19	44.47-46.06	671.16<br>1006.41	670.92<br>1005.88	$M+3H^+$<br>$M+2H^+$	H2HN2NA1C-PA	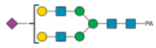	366(H1HN1)<br>657(H1HN1NA1)<br>1192(H3HN3-PA)	29.45	Standard a<br>α2,6-Sia
	pk.3-18	1	46.80	46.27-47.37	1036.60	1036.05	$M+3H^+$	H5HN5NA1C-PA	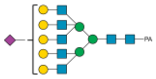	366(H1HN1)<br>657(H1HN1NA1)	3.56	α2,6-Sia
	pk.3-19	1	48.16	47.51-48.48	841.55<br>1261.59	841.32<br>1261.48	$M+3H^+$<br>$M+2H^+$	H3HN3F1NA1C-PA	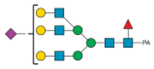	366(H1HN1)<br>657(H1HN1NA1)<br>1339(H3HN3F1-PA)	3.27	α2,3-Sia
		2			1078.58	1078.91	$M+2H^+$	H2HN2F1NA1C-PA	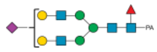	366(H1HN1)<br>657(H1HN1NA1)<br>1135(F1C-PA)<br>1339(H3HN3F1-PA)	2.76	α2,3-Sia<br>artifact (epimer of pk.3-30-1)
	pk.3-20	1	48.92	48.55-49.17	963.30<br>1444.34	963.03<br>1444.04	$M+3H^+$<br>$M+2H^+$	H4HN4F1NA1C-PA	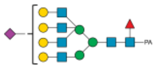	366(H1HN1)<br>657(H1HN1NA1)	4.37	α2,6-Sia
	pk.3-21	1	49.75	49.31-49.93	896.51	896.34	$M+2H^+$	H1HN1F1NA1C-PA	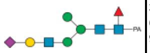	366(H1HN1)<br>657(H1HN1NA1)<br>973(H2HN2F1-PA)<br>1135(F1C-PA)	3.40	α2,6-Sia
		2			671.02<br>1006.64	670.92<br>1005.88	$M+3H^+$<br>$M+2H^+$	H2HN2NA1C-PA	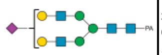	366(H1HN1)<br>657(H1HN1NA1)<br>1192(H3HN3-PA)	1.87	Standard b<br>α2,6-Sia
		3			977.42	977.37	$M+2H^+$	H2HN1F1NA1C-PA	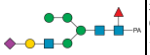	366(H1HN1)<br>657(H1HN1NA1)<br>1135(F1C-PA)<br>1298(H4HN2F1-PA)	0.64	α2,6-Sia
	pk.3-22	1	50.37	50.07-50.97	670.92<br>1006.37	670.92<br>1005.88	$M+3H^+$<br>$M+2H^+$	H2HN2NA1C-PA	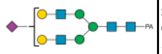	366(H1HN1)<br>657(H1HN1NA1)<br>1192(H3HN3-PA)	6.54	Standard c<br>α2,3-Sia
		2			841.38<br>1261.50	841.32<br>1261.48	$M+3H^+$<br>$M+2H^+$	H3HN3F1NA1C-PA	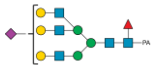	366(H1HN1)<br>657(H1HN1NA1)<br>1135(F1C-PA)	2.84	α2,3-Sia

Table S1B Continued.

Fr. No. (DEAE)	Peak. No. (ODS)	Full MS No.	Elusion time max (min)	Elusion time range (min)	Observed parent ion (*m/z* value)	Calculated (*m/z* value)	Estimated adduct	Estimated composition[a), c)]	Deduced glycan structure[b)]	Characteristic fragments[a)]	Relative amounts[d)]	Notes[f), g)]
	pk.3-23	1	52.22	51.04-52.70	670.99<br>1005.94	670.92<br>1005.88	M+3H+<br>M+2H+	H2HN2NA1C-PA	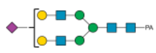	366(H1HN1)<br>657(H1HN1NA1)<br>1192(H3HN3-PA)	2.88	Standard d<br>α2,3-Sia
		2			963.01	963.03	M+3H+	H4HN4F1NA1C-PA	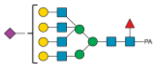	366(H1HN1)<br>657(H1HN1NA1)	0.69	α2,3-Sia
		3			793.13<br>1188.60	792.63<br>1188.45	M+3H+<br>M+2H+	H3HN3NA1C-PA	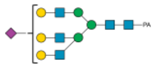	366(H1HN1)<br>657(H1HN1NA1)	4.88	α2,6-Sia
		4			841.38<br>1261.89	841.32<br>1261.48	M+3H+<br>M+2H+	H3HN3F1NA1C-PA	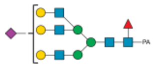	366(H1HN1)<br>657(H1HN1NA1)<br>1339(H3HN3F1-PA)	4.08	α2,3-Sia
	pk.3-24	1	53.18	52.84-53.46	679.34<br>1018.46	679.27<br>1018.40	M+3H+<br>M+2H+	HN3F1NA1C-PA	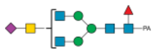	407(HN2)<br>698(HN2NA1)<br>973(H2HN2F1-PA)<br>1135(F1C-PA)	2.85	sLacdiNAc with α2,6-Sia
		2			994.37	993.85	M+2H+	H1HN3F1(SO3)1C-PA	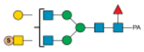	366(H1HN1)<br>407(HN2)<br>1339(H3HN3F1-PA)<br>1622(HN2F1(SO3)1C-PA)	0.52	LacdiNAc with SO3
	pk.3-25	1	54.91	53.60-55.60	733.47<br>1099.73	733.28<br>1099.42	M+3H+<br>M+2H+	H1HN3F1NA1C-PA	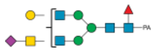	366(H1HN1)<br>698(HN2NA1)<br>973(H2HN2F1-PA)<br>1135(F1C-PA)	13.15	sLacdiNAc with α2,6-Sia
		2			787.48<br>1180.29	787.30<br>1180.45	M+3H+<br>M+2H+	H2HN3F1NA1C-PA	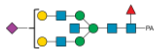	366(H1HN1)<br>657(H1HN1NA1)<br>1135(F1C-PA)<br>1339(H3HN3F1-PA)	2.37	α2,6-Sia artifact (epimer of pk.3-35-1)
		3			963.31	963.03	M+3H+	H4HN4F1NA1C-PA	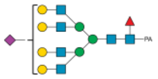	366(H1HN1)<br>657(H1HN1NA1)	1.17	α2,6-Sia
		4			977.25	977.37	M+2H+	H2HN1F1NA1C-PA	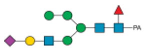	366(H1HN1)<br>657(H1HN1NA1)<br>973(H2HN2F1-PA)<br>1135(F1C-PA)<br>1298(H4HN2F1-PA)	1.60	α2,3-Sia
		5			998.00	997.88	M+2H+	H1HN2F1NA1C-PA	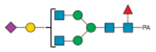	366(H1HN1)<br>657(H1HN1NA1)<br>973(H2HN2F1-PA)<br>1135(F1C-PA)	2.43	α2,6-Sia
		6			896.28	896.34	M+2H+	H1HN1F1NA1C-PA	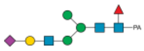	366(H1HN1)<br>657(H1HN1NA1)<br>973(H2HN2F1-PA)<br>1135(F1C-PA)	0.68	α2,3-Sia
	pk.3-26	1	56.38	55.67-56.99	719.89<br>1078.97	719.61<br>1078.91	M+3H+<br>M+2H+	H2HN2F1NA1C-PA	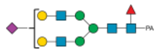	366(H1HN1)<br>657(H1HN1NA1)<br>973(H2HN2F1-PA)<br>1135(F1C-PA)	26.14	Standard e<br>α2,6-Sia
		2			1084.84	1084.74	M+3H+	H5HN5F1NA1C-PA	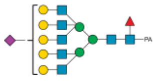	366(H1HN1)<br>657(H1HN1NA1)<br>1704(H1HN2F1C-PA)	3.99	α2,6-Sia
	pk.3-27	1	57.46	57.06-58.30	738.82<br>1107.49	738.62<br>1107.42	M+3H+<br>M+2H+	H2HN3NA1C-PA	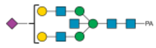	366(H1HN1)<br>657(H1HN1NA1)<br>1192(H3HN3-PA)	7.99	α2,6-Sia
		2			973.23	973.34	M+2H+	H2HN2F1(SO3)1C-PA	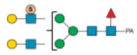	366(H1HN1)<br>446(H1HN1(SO3)1)<br>1135(F1C-PA)	2.58	LacNAc with SO3
	pk.3-28	1	59.05	58.37-59.68	747.15<br>1120.03	746.96<br>1119.94	M+3H+<br>M+2H+	HN4F1NA1C-PA	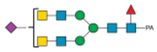	407(HN2)<br>698(HN2NA1)<br>973(H2HN2F1-PA)<br>1135(F1C-PA)	3.54	LacdiNAc, sLacdiNAc with α2,6-Sia
		2			1079.15	1078.91	M+2H+	H2HN2F1NA1C-PA	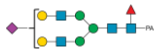	366(H1HN1)<br>657(H1HN1NA1)<br>1135(F1C-PA)<br>1339(H3HN3F1-PA)	1.36	Standard f<br>α2,6-Sia

Table S1B Continued.

Fr. No. (DEAE)	Peak. No. (ODS)	Full MS No.	Elusion time max (min)	Elusion time range (min)	Observed parent ion (*m/z* value)	Calculated (*m/z* value)	Estimated adduct	Estimated composition[a), c)]	Deduced glycan structure[b)]	Characteristic fragments[a)]	Relative amounts[d)]	Notes[f), g)]
		3			963.45	963.03	$M+3H^+$	H4HN4F1NA1C-PA	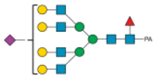	366(H1HN1)<br>657(H1HN1NA1)<br>1339(H3HN3F1-PA)	0.81	α2,3-Sia
		4			1206.65	1206.45	$M+3H^+$	H6HN6F1NA1C-PA	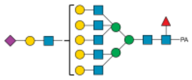	366(H1HN1)<br>657(H1HN1NA1)<br>731(H2HN2)<br>1022(H2HN2NA1)	0.87	sLacNAc repeat with α2,3-Sia
	pk.3-29	1	60.35	59.75-61.34	997.83	997.88	$M+2H^+$	H1HN2F1NA1C-PA	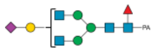	366(H1HN1)<br>657(H1HN1NA1)<br>1135(F1C-PA)	4.19	α2,3-Sia
		2			963.23	963.03	$M+3H^+$	H4HN4F1NA1C-PA	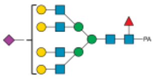	366(H1HN1)<br>657(H1HN1NA1)<br>1339(H3HN3F1-PA)	3.32	α2,3-Sia
		3			1099.49	1099.42	$M+2H^+$	H1HN3F1NA1C-PA	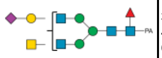	366(H1HN1)<br>407(HN2)<br>657(H1HN1NA1)	1.42	LacdiNAc, α2,6-Sia
	pk.3-30	1	62.24	61.41-63.63	719.78<br>1079.30	719.61<br>1078.91	$M+3H^+$<br>$M+2H^+$	H2HN2F1NA1C-PA	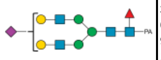	366(H1HN1)<br>657(H1HN1NA1)<br>973(H2HN2F1-PA)<br>1135(F1C-PA)	38.12	α2,3-Sia
		2			1153.25	1152.43	$M+3H^+$	H5HN6F1NA1C-PA	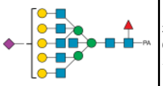	366(H1HN1)<br>657(H1HN1NA1)<br>1907(H1HN3F1C-PA)	1.29	α2,6-Sia
	pk.3-31	1	64.31	63.83-64.94	841.46<br>1261.61	841.32<br>1261.48	$M+3H^+$<br>$M+2H^+$	H3HN3F1NA1C-PA	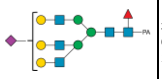	366(H1HN1)<br>657(H1HN1NA1)<br>1339(H3HN3F1-PA)	5.80	α2,6-Sia
	pk.3-32	1	65.26	65.08-66.18	1107.43	1107.42	$M+2H^+$	H2HN3NA1C-PA	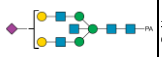	366(H1HN1)<br>657(H1HN1NA1)	1.30	α2,3-Sia
		2			1030.74	1030.72	$M+3H^+$	H4HN5F1NA1C-PA	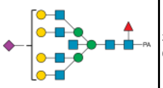	366(H1HN1)<br>657(H1HN1NA1)	1.61	α2,6-Sia
	pk.3-33	1	67.80	67.01-68.26	841.69<br>1261.78	841.32<br>1261.48	$M+3H^+$<br>$M+2H^+$	H3HN3F1NA1C-PA	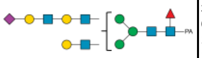	366(H1HN1)<br>657(H1HN1NA1)<br>1022(H2HN2NA1)<br>1135(F1C-PA)	3.34	sLacNAc repeat with α2,3-Sia
	pk.3-34	1	68.91	68.33-69.57	733.60<br>1099.54	733.28<br>1099.42	$M+3H^+$<br>$M+2H^+$	H1HN3F1NA1C-PA	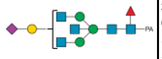	366(H1HN1)<br>657(H1HN1NA1)<br>1135(F1C-PA)<br>1339(H3HN3F1-PA)	3.72	α2,6-Sia
		2			860.27	860.33	$M+3H^+$	H3HN4NA1C-PA	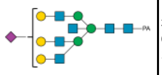	366(H1HN1)<br>657(H1HN1NA1)<br>1192(H3HN3-PA)	0.51	α2,6-Sia
	pk.3-35	1	72.55	71.65-73.24	787.82<br>1180.86	787.30<br>1180.45	$M+3H^+$<br>$M+2H^+$	H2HN3F1NA1C-PA	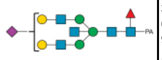	366(H1HN1)<br>657(H1HN1NA1)<br>973(H2HN2F1-PA)<br>1135(F1C-PA)	37.34	Standard g<br>α2,6-Sia
	pk.3-36	1	73.56	73.38-74.14	841.59<br>1261.99	841.32<br>1261.48	$M+3H^+$<br>$M+2H^+$	H3HN3F1NA1C-PA	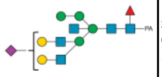	366(H1HN1)<br>657(H1HN1NA1)<br>1339(H3HN3F1-PA)	2.91	α2,6-Sia
	pk.3-37	1	74.48	74.18-74.84	963.15	963.03	$M+3H^+$	H4HN4F1NA1C-PA	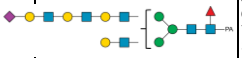	366(H1HN1)<br>657(H1HN1NA1)<br>731(H2HN2)<br>1022(H2HN2NA1)<br>1096(H3HN3)	1.72	sLacNAc repeat with α2,3-Sia
	pk.3-38	1	75.94	75.26-76.91	801.17<br>1200.78	800.98<br>1200.96	$M+3H^+$<br>$M+2H^+$	H1HN4F1NA1C-PA	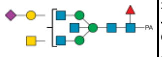	366(H1HN1)<br>407(HN2)<br>657(H1HN1NA1)<br>1339(H3HN3F1-PA)	0.95	LacdiNAc, α2,6-Sia
		2			733.95<br>1099.66	733.28<br>1099.42	$M+3H^+$<br>$M+2H^+$	H1HN3F1NA1C-PA	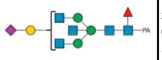	366(H1HN1)<br>657(H1HN1NA1)<br>1339(H3HN3F1-PA)	1.22	α2,6-Sia

Table S1B Continued.

Fr. No. (DEAE)	Peak. No. (ODS)	Full MS No.	Elusion time max (min)	Elusion time range (min)	Observed parent ion (*m/z* value)	Calculated (*m/z* value)	Estimated adduct	Estimated composition[a), c)]	Deduced glycan structure[b)]	Characteristic fragments[a)]	Relative amounts[d)]	Notes[f), g)]
		3			841.61<br>1261.36	841.32<br>1261.48	M+3H$^+$<br>M+2H$^+$	H3HN3F1NA1C-PA	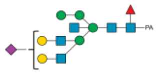	366(H1HN1)<br>657(H1HN1NA1)<br>1339(H3HN3F1-PA)	1.04	α2,3-Sia
	pk.3-39	1	78.07	77.25-78.84	787.29<br>1180.99	787.30<br>1180.45	M+3H$^+$<br>M+2H$^+$	H2HN3F1NA1C-PA	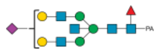	366(H1HN1)<br>657(H1HN1NA1)<br>1339(H3HN3F1-PA)<br>1542(H3HN4F1-PA)	5.59	α2,3-Sia
	pk.3-40	1	79.30	78.79-79.33	1099.48	1099.42	M+2H$^+$	H1HN3F1NA1C-PA	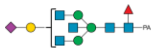	366(H1HN1)<br>657(H1HN1NA1)<br>1339(H3HN3F1-PA)	1.72	α2,3-Sia
	pk.3-41	1	80.35	79.67-81.12	909.11<br>1363.39	909.01<br>1363.01	M+3H$^+$<br>M+2H$^+$	H3HN4F1NA1C-PA	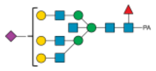	366(H1HN1)<br>657(H1HN1NA1)<br>1339(H3HN3F1-PA)	5.86	α2,6-Sia
		2			787.56<br>1180.40	787.30<br>1180.45	M+3H$^+$<br>M+2H$^+$	H2HN3F1NA1C-PA	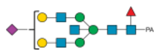	366(H1HN1)<br>657(H1HN1NA1)<br>1339(H3HN3F1-PA)	3.12	α2,3-Sia
	pk.3-42	1	81.98	81.33-82.78	909.42<br>1363.29	909.01<br>1363.01	M+3H$^+$<br>M+2H$^+$	H3HN4F1NA1C-PA	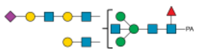	366(H1HN1)<br>657(H1HN1NA1)<br>731(H2HN2)<br>1022(H2HN2NA1)<br>1339(H3HN3F1-PA)	1.59	sLacNAc repeat with α2,3-Sia
		2			963.19			data not available			0.86	xMS2
fr.4	pk.4-1	1	12.89	12.52-13.21	858.98	859.29	M+2H$^+$	H4(HPO3)1C-PA	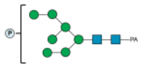	243(H1(HPO3)1)<br>325(H2)<br>405(H2(HPO3)1)<br>1151(H4HN2-PA)	0.70	HPO3
	pk.4-2	1	13.86	13.42-14.32	742.78<br>1113.48			data not available			1.68	(artifact)
		2			1102.14			data not available			1.02	(artifact)
	pk.4-3	1	15.66	15.28-15.84	1020.92			data not available			0.50	non-glycan
	pk.4-4	1	16.09	15.91-16.60	550.30	550.22	M+H$^+$	H1NA1-PA	unknown		1.11	non-*N*-glycan
	pk.4-5	1	19.00	18.46-19.50	858.92	859.29	M+2H$^+$	H4(HPO3)1C-PA	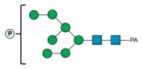	243(H1(HPO3)1)<br>405(H2(HPO3)1)<br>1151(H4HN2-PA)	4.11	HPO3
	pk.4-6	1	22.44	22.13-22.68	753.41	753.30	M+H$^+$	H1HN1NA1-PA	unknown		0.15	non-*N*-glycan
		2			1187.00			data not available			0.23	(artifact)
	pk.4-7	1	37.31	36.79-37.90	768.02<br>1151.80	767.95<br>1151.43	M+3H$^+$<br>M+2H$^+$	H2HN2NA2C-PA	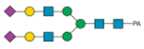	366(H1HN1)<br>657(H1HN1NA1)<br>1192(H3HN3-PA)	0.82	α2,6-Sia, α2,6-Sia artifact (epimer of pk.4-15-1)
	pk.4-8	1	38.49	38.17-38.93	775.38			data not available			0.63	non-glycan
	pk.4-9	1	41.51	40.94-41.84	889.56<br>1334.65	889.67<br>1333.99	M+3H$^+$<br>M+2H$^+$	H3HN3NA2C-PA	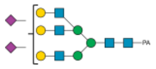	366(H1HN1)<br>657(H1HN1NA1)<br>1355(H1HN1C-PA)	0.35	

Table S1B Continued.

Fr. No. (DEAE)	Peak. No. (ODS)	Full MS No.	Elusion time max (min)	Elusion time range (min)	Observed parent ion (*m/z* value)	Calculated (*m/z* value)	Estimated adduct	Estimated composition[a), c)]	Deduced glycan structure[b)]	Characteristic fragments[a)]	Relative amounts[d)]	Notes[f), g)]
	pk.4-10	1	42.31	42.05-42.81	890.17	889.67	$M+3H^+$	H3HN3NA2C-PA	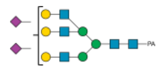	366(H1HN1)<br>657(H1HN1NA1)<br>1355(H1HN1C-PA)	0.46	
	pk.4-11	1	43.72	43.15-44.19	889.69<br>1333.95	889.67<br>1333.99	$M+3H^+$<br>$M+2H^+$	H3HN3NA2C-PA	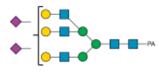	366(H1HN1)<br>657(H1HN1NA1)<br>1355(H1HN1C-PA)	0.80	
	pk.4-12	1	45.02	44.47-45.30	890.19<br>1334.04	889.67<br>1333.99	$M+3H^+$<br>$M+2H^+$	H3HN3NA2C-PA	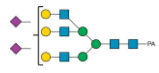	366(H1HN1)<br>657(H1HN1NA1)<br>1355(H1HN1C-PA)	0.71	
	pk.4-13	1	46.31	45.37-46.89	889.58<br>1334.41	889.67<br>1333.99	$M+3H^+$<br>$M+2H^+$	H3HN3NA2C-PA	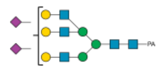	366(H1HN1)<br>657(H1HN1NA1)<br>1355(H1HN1C-PA)	1.79	
	pk.4-14	1	47.43	46.96-48.06	816.83<br>1225.10	816.64<br>1224.46	$M+3H^+$<br>$M+2H^+$	H2HN2F1NA2C-PA	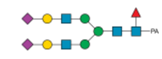	366(H1HN1)<br>657(H1HN1NA1)<br>973(H2HN2F1-PA)<br>1135(F1C-PA)	1.26	α2,6-Sia, α2,6-Sia artifact (epimer of pk.4-20-1)
	pk.4-15	1	49.05	48.27-50.00	768.14<br>1151.87	767.95<br>1151.43	$M+3H^+$<br>$M+2H^+$	H2HN2NA2C-PA	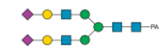	366(H1HN1)<br>657(H1HN1NA1)<br>1192(H3HN3-PA)	20.94	Standard A<br>α2,6-Sia, α2,6-Sia
	pk.4-16	1	50.86	50.21-52.14	768.16<br>1151.88	767.95<br>1151.43	$M+3H^+$<br>$M+2H^+$	H2HN2NA2C-PA	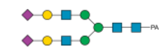	366(H1HN1)<br>657(H1HN1NA1)<br>1192(H3HN3-PA)	2.79	α2,3-Sia, α2,6-Sia
		2			1060.06	1060.06	$M+3H^+$	H4HN4F1NA2C-PA	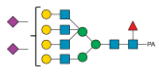	366(H1HN1)<br>657(H1HN1NA1)<br>1704(H1HN2F1C-PA)	1.26	
	pk.4-17	1	53.15	52.62-53.48	938.49<br>1407.58	938.35<br>1407.02	$M+3H^+$<br>$M+2H^+$	H3HN3F1NA2C-PA	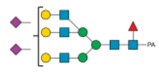	366(H1HN1)<br>657(H1HN1NA1)<br>1339(H3HN3F1-PA)	1.64	
	pk.4-18	1	55.24	54.50-55.81	938.35<br>1407.37	938.35<br>1407.02	$M+3H^+$<br>$M+2H^+$	H3HN3F1NA2C-PA	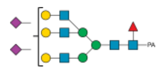	366(H1HN1)<br>657(H1HN1NA1)<br>1339(H3HN3F1-PA)	2.26	
		2			889.63<br>1334.60	889.67<br>1333.99	$M+3H^+$<br>$M+2H^+$	H3HN3NA2C-PA	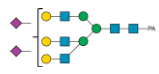	366(H1HN1)<br>657(H1HN1NA1)	1.04	
	pk.4-19	1	56.59	55.95-57.40	889.71<br>1334.98	889.67<br>1333.99	$M+3H^+$<br>$M+2H^+$	H3HN3NA2C-PA	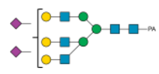	366(H1HN1)<br>657(H1HN1NA1)	6.37	
	pk.4-20	1	59.02	58.23-60.10	817.28<br>1224.55	816.64<br>1224.46	$M+3H^+$<br>$M+2H^+$	H2HN2F1NA2C-PA	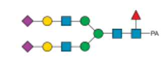	366(H1HN1)<br>657(H1HN1NA1)<br>973(H2HN2F1-PA)<br>1135(F1C-PA)	9.00	Standard B<br>α2,6-Sia, α2,6-Sia
		2			1181.93	1181.77	$M+3H^+$	H5HN5F1NA2C-PA	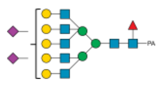	366(H1HN1)<br>657(H1HN1NA1)	3.63	
		3			1060.25	1060.06	$M+3H^+$	H4HN4F1NA2C-PA	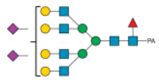	366(H1HN1)<br>657(H1HN1NA1)<br>1501(H1HN1F1C-PA)	0.51	
	pk.4-21	1	61.68	60.44-61.76	830.48<br>1245.04	830.32<br>1244.97	$M+3H^+$<br>$M+2H^+$	H1HN3F1NA2C-PA	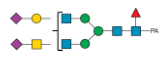	657(H1HN1NA1)<br>698(HN2NA1)<br>973(H2HN2F1-PA)<br>1135(F1C-PA)	1.74	sLacdiNAc with α2,6-Sia,<br>α2,3-Sia
		2			1182.12	1181.77	$M+3H^+$	H5HN5F1NA2C-PA	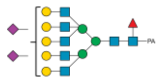	366(H1HN1)<br>657(H1HN1NA1)<br>1704(H1HN2F1C-PA)	1.35	
		3			773.83<br>1159.79	773.61<br>1159.91	$M+3H^+$<br>$M+2H^+$	HN4F1NA1(SO3)1C-PA	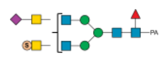	407(HN2)<br>487(HN2(SO3)1)<br>698(HN2NA1)<br>1339(H3HN3F1-PA)<br>1418(H3HN3F1(SO3)1-PA)	1.10	LacdiNAc with SO3, sLacdiNAc with α2,6-Sia

Table S1B Continued.

Fr. No. (DEAE)	Peak. No. (ODS)	Full MS No.	Elusion time max (min)	Elusion time range (min)	Observed parent ion (*m/z* value)	Calculated (*m/z* value)	Estimated adduct	Estimated composition[a), c)]	Deduced glycan structure[b)]	Characteristic fragments[a)]	Relative amounts[d)]	Notes[f), g)]
	pk.4-22	1	62.50	61.96-63.28	816.68<br>1225.09	816.64<br>1224.46	$M+3H^+$<br>$M+2H^+$	H2HN2F1NA2C-PA	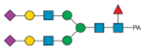	366(H1HN1)<br>657(H1HN1NA1)<br>973(H2HN2F1-PA)<br>1135(F1C-PA)	4.35	α2,3-Sia, α2,6-Sia
		2			1182.22	1181.77	$M+3H^+$	H5HN5F1NA2C-PA	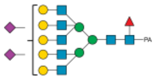	366(H1HN1)<br>657(H1HN1NA1)<br>1704(H1HN2F1C-PA)	0.50	
		3			1060.13	1060.06	$M+3H^+$	H4HN4F1NA2C-PA	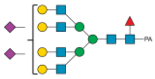	366(H1HN1)<br>657(H1HN1NA1)<br>1704(H1HN2F1C-PA)	0.42	
		4			1303.62	1303.48	$M+3H^+$	H6HN6F1NA2C-PA	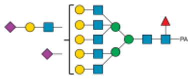	366(H1HN1)<br>657(H1HN1NA1)<br>731(H2HN2)<br>1022(H2HN2NA1)	0.38	sLacNAc repeat
	pk.4-23	1	64.91	64.39-65.76	1304.54	1303.48	$M+3H^+$	H6HN6F1NA2C-PA	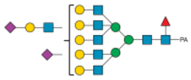	366(H1HN1)<br>657(H1HN1NA1)<br>731(H2HN2)<br>1022(H2HN2NA1)	2.25	sLacNAc repeat
	pk.4-24	1	66.91	65.84-67.43	939.03<br>1407.05	938.35<br>1407.02	$M+3H^+$<br>$M+2H^+$	H3HN3F1NA2C-PA	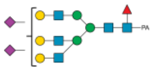	366(H1HN1)<br>657(H1HN1NA1)<br>973(H2HN2F1-PA)	3.24	
		2			1059.99	1060.06	$M+3H^+$	H4HN4F1NA2C-PA	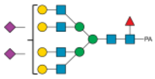	366(H1HN1)<br>657(H1HN1NA1)<br>1339(H3HN3F1-PA)	1.47	
	pk.4-25	1	68.21	67.50-69.29	816.65<br>1224.76	816.64<br>1224.46	$M+3H^+$<br>$M+2H^+$	H2HN2F1NA2C-PA	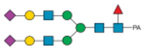	366(H1HN1)<br>657(H1HN1NA1)<br>973(H2HN2F1-PA)<br>1135(F1C-PA)	7.11	α2,3-Sia, α2,3-Sia
		2			938.66<br>1407.22	938.35<br>1407.02	$M+3H^+$<br>$M+2H^+$	H3HN3F1NA2C-PA	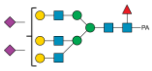	366(H1HN1)<br>657(H1HN1NA1)<br>973(H2HN2F1-PA)	1.41	
	pk.4-26	1	73.42	72.61-74.07	884.45<br>1326.03	884.33<br>1326.00	$M+3H^+$<br>$M+2H^+$	H2HN3F1NA2C-PA	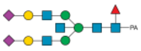	366(H1HN1)<br>657(H1HN1NA1)<br>1339(H3HN3F1-PA)	5.09	Standard D<br>α2,6-Sia, α2,6-Sia
	pk.4-27	1	74.46	74.20-75.03	938.41<br>1406.78	938.35<br>1407.02	$M+3H^+$<br>$M+2H^+$	H3HN3F1NA2C-PA	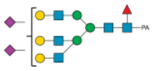	366(H1HN1)<br>657(H1HN1NA1)<br>973(H2HN2F1-PA)	1.83	
	pk.4-28	1	76.45	75.86-77.04	884.46<br>1326.45	884.33<br>1326.00	$M+3H^+$<br>$M+2H^+$	H2HN3F1NA2C-PA	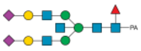	366(H1HN1)<br>657(H1HN1NA1)<br>973(H2HN2F1-PA)	2.77	α2,3-Sia, α2,6-Sia
		2			938.35<br>1407.07	938.35<br>1407.02	$M+3H^+$<br>$M+2H^+$	H3HN3F1NA2C-PA	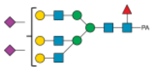	366(H1HN1)<br>657(H1HN1NA1)<br>973(H2HN2F1-PA)	0.98	
	pk.4-29	1	81.45	80.84-81.81	1006.09	1006.04	$M+3H^+$	H3HN4F1NA2C-PA	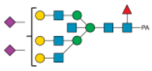	366(H1HN1)<br>657(H1HN1NA1)<br>1501(H1HN1F1C-PA)	0.96	
		2			884.39	884.33	$M+3H^+$	H2HN3F1NA2C-PA	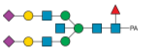	366(H1HN1)<br>657(H1HN1NA1)<br>1339(H3HN3F1-PA)	0.48	α2,3-Sia, α2,3-Sia
	pk.4-30	1	82.58	81.95-83.13	1006.60	1006.04	$M+3H^+$	H3HN4F1NA2C-PA	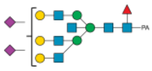	366(H1HN1)<br>657(H1HN1NA1)<br>1542(H3HN4F1-PA)	2.04	
	pk.4-31	1	83.72	83.20-84.23	1006.29	1006.04	$M+3H^+$	H3HN4F1NA2C-PA	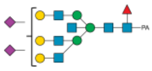	366(H1HN1)<br>657(H1HN1NA1)<br>1542(H3HN4F1-PA)	0.93	
fr.5	pk.5-1	1	14.41	13.86-14.96	777.98	778.26	$M+2H^+$	H3(HPO3)1C-PA	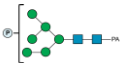	243(H1(HPO3)1)<br>1151(H4HN2-PA)	4.11	HPO3

Table S1B Continued.

Fr. No. (DEAE)	Peak. No. (ODS)	Full MS No.	Elusion time max (min)	Elusion time range (min)	Observed parent ion (*m/z* value)	Calculated (*m/z* value)	Estimated adduct	Estimated composition[a), c)]	Deduced glycan structure[b)]	Characteristic fragments[a)]	Relative amounts[d)]		Notes[f), g)]
		2			696.91	697.24	$M+2H^+$	H2(HPO3)1C-PA	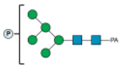	243(H1(HPO3)1)<br>405(H2(HPO3)1)	0.25		HPO3
	pk.5-2	1	22.09	21.55-22.65	777.96	778.26	$M+2H^+$	H3(HPO3)1C-PA	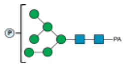	243(H1(HPO3)1)<br>405(H2(HPO3)1)<br>1151(H4HN2-PA)	0.85		HPO3
	pk.5-3	1	43.41	42.96-43.93				data not available			0.49		xMS
	pk.5-4	1	46.76	46.40-47.36	986.61	986.70	$M+3H^+$	H3HN3NA3C-PA	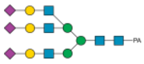	366(H1HN1)<br>657(H1HN1NA1)<br>1192(H3HN3-PA)	0.70		
	pk.5-5	1	53.71	53.19-55.11	1013.33	1013.32	$M+2H^+$	H2HN2F1(SO3)2C-PA	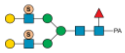	366(H1HN1)<br>446(H1HN1(SO3)1)<br>1135(F1C-PA)	2.14		LacNAc with SO3,<br>LacNAc with SO3
	pk.5-6	1	58.22	57.58-58.82	987.24<br>1480.10	986.70<br>1479.54	$M+3H^+$<br>$M+2H^+$	H3HN3NA3C-PA	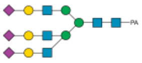	366(H1HN1)<br>657(H1HN1NA1)<br>1355(H1HN1C-PA)	2.11		
		2			1279.13	1278.80	$M+3H^+$	H5HN5F1NA3C-PA	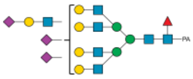	366(H1HN1)<br>657(H1HN1NA1)<br>1022(H2HN2NA1)	0.27		sLacNAc repeat
	pk.5-7	1	60.49	59.85-61.43	959.72<br>1279.12	959.36<br>1278.80	$M+4H^+$<br>$M+3H^+$	H5HN5F1NA3C-PA	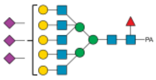	366(H1HN1)<br>657(H1HN1NA1)	2.94		
	pk.5-8	1	62.34	61.77-63.76	959.57<br>1279.19	959.36<br>1278.80	$M+4H^+$<br>$M+3H^+$	H5HN5F1NA3C-PA	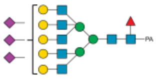	366(H1HN1)<br>657(H1HN1NA1)	2.53		
		2			987.23	986.70	$M+3H^+$	H3HN3NA3C-PA	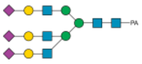	366(H1HN1)<br>657(H1HN1NA1)	0.91		
		3			1157.31	1157.09	$M+3H^+$	H4HN4F1NA3C-PA	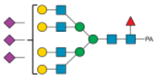	657(H1HN1NA1)	0.36		
	pk.5-9	1	66.46	65.48-67.60	1157.50	1157.09	$M+3H^+$	H4HN4F1NA3C-PA	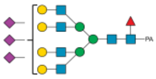	366(H1HN1)<br>657(H1HN1NA1)<br>1339(H3HN3F1-PA)	2.40		
	pk.5-10	1	68.68	67.81-69.46	1035.36	1035.38	$M+3H^+$	H3HN3F1NA3C-PA	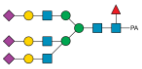	366(H1HN1)<br>657(H1HN1NA1)<br>973(H2HN2F1-PA)	1.81		
	pk.5-11	1	71.34	70.69-72.07	1035.36	1035.38	$M+3H^+$	H3HN3F1NA3C-PA	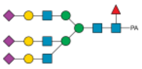	366(H1HN1)<br>657(H1HN1NA1)<br>973(H2HN2F1-PA)<br>1135(F1C-PA)	0.86		
		2			1008.25			data not available			0.70		non-glycan
	pk.5-12	1	76.80	76.12-77.28	1035.65	1035.38	$M+3H^+$	H3HN3F1NA3C-PA	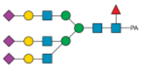	657(H1HN1NA1)<br>973(H2HN2F1-PA)	0.77		
	pk.5-13	1	82.20	81.88-82.70	1103.10	1103.08	$M+3H^+$	H3HN4F1NA3C-PA	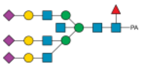	366(H1HN1)<br>657(H1HN1NA1)	0.41		
fr.6	pk.6-1	1	43.42	42.76-43.99	967.46			data not available			1.28		(artifact)

Table S1B Continued.

Fr. No. (DEAE)	Peak. No. (ODS)	Full MS No.	Elusion time max (min)	Elusion time range (min)	Observed parent ion (*m/z* value)	Calculated (*m/z* value)	Estimated adduct	Estimated composition[a), c)]	Deduced glycan structure[b)]	Characteristic fragments[a)]	Relative amounts[d)]	Notes[f), g)]
	pk.6-2	1	44.50	44.06-44.89				data not available			0.43	x MS
	pk.6-3	1	53.61	52.99-54.02	1041.35			data not available			0.57	x MS2
	pk.6-4	1	56.14	55.53-56.97	843.90<br>1265.02	843.29<br>1264.44	$M+3H^+$<br>$M+2H^+$	H2HN2F1NA2(SO3)1C-PA	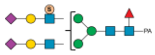	657(H1HN1NA1)<br>1339(H3HN3F1-PA)	1.03	sLacNAc with SO3 and α2,6-Sia, α2,6-Sia
	pk.6-5	1	59.17	58.13-59.92	1054.33	1054.34	$M+2H^+$	HN4F1(SO3)2C-PA	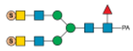	407(HN2)<br>487(HN2(SO3)1)	2.69	LacdiNAc with SO3, LacdiNAc with SO3
	pk.6-6	1	61.55	61.03-62.07	843.84<br>1264.13	843.29<br>1264.44	$M+3H^+$<br>$M+2H^+$	H2HN2F1NA2(SO3)1C-PA	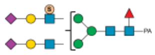	446(H1HN1(SO3)1)<br>657(H1HN1NA1)<br>1418(HN1F1(SO3)1C-PA)	0.75	sLacNAc with SO3 and α2,3-Sia, α2,3-Sia
