# Supplementary material for: Structural analysis of N-glycans in chicken trachea and lung reveals potential receptors of chicken influenza viruses
Source: Sci Rep. 2022 Feb 8;12:2081. doi: 10.1038/s41598-022-05961-x (PMC8827061; doi:10.1038/s41598-022-05961-x)
Supplement: Supplementary file 5 — Supplementary Table S3. [file 41598_2022_5961_MOESM5_ESM.pdf]

Table S2A. MS and MS/MS data for PA-*N*-glycans from chicken trachea after two-step alkylamidation

a) H, hexose; HN, *N*-acetylhexosamine; F, fucose (or deoxyhexose); NA, *N*-acetylneuraminic acid; SO3, sulfate group; HPO3, phosphate group; C, trimannosyl core; MA, methylamine; PA, 2-aminopyridine.

b) Sialyl linkage : ◇: α2,3; ◆ : α2,6

c) Most of the detectable fluorescence peaks eluted in 10–87 min were numbered, but not all peaks were derived from PA-*N*-glycans. The compositions of some peaks could not be determined by MS and MS/MS analysis due to insufficent signals; these cases are indicated as "data not available".

d) Individual peaks detected by fluorescence sometimes included more than two kinds of PA-glycans with different mass values. In such cases, the proportions were estimated using the ratios of integrated ion intensities for each *m/z* value detected at the corresponding elution time.

e) Amounts of glycans relative to the most abundant glycan (pk. 3-31-1), for which the amount was defined as 100.

Fr. No. (DEAE)	Peak. No. (ODS)	Full MS No.	Elusion time max (min)	Elusion time range (min)	Observed parent ion (m/z value)	Calculated (m/z value)	Estimated adduct	Estimated composition[a), b), c)]		Characteristic fragments[a)]	Area[d)]	Relative amounts[e)]	Notes
fr.3	pk.3-1	1	22.06	21.11-22.57	1067.06	1066.88	M+2H$^+$	H1HN3F2(SO3)1C-PA		553(HN2F1)	409862	7.79	LacdiNAc(Fuc)
		2			972.90	973.34	M+2H$^+$	H2HN2F1(SO3)1C-PA		553(HN2F1)	302401	5.75	LacdiNAc(Fuc)
		3			891.95	892.31	M+2H$^+$	H1HN2F1(SO3)1C-PA		553(HN2F1)	148855	2.83	LacdiNAc(Fuc)
	pk.3-2	1	23.35	22.71-23.82	1139.67	1139.91	M+2H$^+$	H1HN3F3(SO3)1C-PA		553(HN2F1)	201237	3.82	LacdiNAc(Fuc)
	pk.3-3	1	25.77	25.42-25.97	710.01<br>1064.45	710.28<br>1064.91	M+3H$^+$<br>M+2H$^+$	H3HN1F1NA1C-PA	◇1	816(H1HN1F1NA1)	912419	17.34	sLe^X
	pk.3-4	1	26.82	26.39-27.22	760.18<br>1140.04	760.28<br>1139.91	M+3H$^+$<br>M+2H$^+$	H1HN3F3(SO3)1C-PA		553(HN2F1)	2129219	40.46	LacdiNAc(Fuc)
		2			1166.43	1166.45	M+2H$^+$	H3HN2F1NA1C-PA	◇1	816(H1HN1F1NA1)	409661	7.78	sLe^X
		3			988.40			data not available			710884	13.51	
		4			1119.33	1119.40	M+2H$^+$	H2HN2F3(SO3)1C-PA		512(H1HN1F1)	124904	2.37	Le^X
	pk.3-5	1	27.63	27.29-27.92	965.07	965.34	M+2H$^+$	H1HN2F2(SO3)1C-PA		553(HN2F1)	538525	10.23	LacdiNAc(Fuc)
		2			1160.66	1160.42	M+2H$^+$	HN4F3(SO3)1C-PA		553(HN2F1)	307891	5.85	LacdiNAc(Fuc)
		3			884.44	884.32	M+2H$^+$	HN2F2(SO3)1C-PA		553(HN2F1)	174170	3.31	LacdiNAc(Fuc)
	pk.3-6	1	28.21	27.99-28.33	987.37			data not available			499392	9.49	
	pk.3-7	1	28.64	28.40-28.89	1134.60			data not available			305930	5.81	
		2			987.32			data not available			162183	3.08	
		3			1332.72			data not available			148443	2.82	
		4			1247.22			data not available			68967	1.31	
	pk.3-8	1	29.64	29.17-29.79	821.17<br>1231.32	821.32<br>1231.48	M+3H$^+$<br>M+2H$^+$	H2HN2F3NA1C-PA	◇1	816(H1HN1F1NA1)	595033	11.31	sLe^X
		2			839.71<br>1260.03	840.33<br>1259.99	M+3H$^+$<br>M+2H$^+$	H2HN3F2NA1C-PA	◇1	816(H1HN1F1NA1)	358893	6.82	sLe^X
		3			1085.34	1085.42	M+2H$^+$	H2HN2F1NA1C-PA	◇1	816(H1HN1F1NA1)	163507	3.11	sLe^X
	pk.3-9	1	30.18	29.86-30.63	821.87<br>1231.67	821.32<br>1231.48	M+3H$^+$<br>M+2H$^+$	H2HN2F3NA1C-PA	◇1	816(H1HN1F1NA1)	1360893	25.86	sLe^X
		2			838.99			data not available			89270	1.70	xMS2
		3			991.51	991.88	M+2H$^+$	H3HN1NA1C-PA	◇1		152115	2.89	
		4			1067.02			data not available			108964	2.07	
	pk.3-10	1	31.27	30.76-31.67	1061.46			data not available			670935	12.75	
		2			1056.69	1056.91	M+2H$^+$	H2HN1F2NA1C-PA	◇1	816(H1HN1F1NA1)	229369	4.36	sLe^X
		3			1089.73			data not available			348334	6.62	
	pk.3-11	1	32.17	31.74-32.57	888.96<br>1332.91	889.02<br>1333.02	M+3H$^+$<br>M+2H$^+$	H2HN3F3NA1C-PA	◇1	816(H1HN1F1NA1)	1812571	34.44	sLe^X
		2			1076.97	1077.43	M+2H$^+$	H1HN2F2NA1C-PA	◇1		105964	2.01	
	pk.3-12	1	32.94	32.64-33.20	824.37<br>1235.94			data not available			704150	13.38	
		2			1012.32	1012.40	M+2H$^+$	H2HN2NA1C-PA	◇1		57099	1.08	
		3			1158.14	1158.45	M+2H$^+$	H2HN2F2NA1C-PA	◇1		26002	0.49	
	pk.3-13	1	33.71	33.33-34.31	888.87<br>1332.47	889.02<br>1333.02	M+3H$^+$<br>M+2H$^+$	H2HN3F3NA1C-PA	◇1	816(H1HN1F1NA1)	1822379	34.63	sLe^X
		2			1158.21	1158.45	M+2H$^+$	H2HN2F2NA1C-PA	◇1	816(H1HN1F1NA1)	296571	5.64	sLe^X
		3			1012.10	1012.40	M+2H$^+$	H2HN2NA1C-PA	◇1		300719	5.71	
	pk.3-14	1	34.82	34.38-35.35	1186.48	1186.96	M+2H$^+$	H2HN3F1NA1C-PA	◇1	816(H1HN1F1NA1)	359566	6.83	sLe^X
	pk.3-15	1	35.61	35.49-35.97	983.78	983.89	M+2H$^+$	H2HN1F1NA1C-PA	◇1		315546	6.00	
	pk.3-16	1	36.46	36.18-36.74	1085.24	1085.43	M+2H$^+$	H2HN2F1NA1C-PA	◇1		211941	4.03	
		2			1025.54	1026.41	M+2H$^+$	H2HN2NA1C-PA	◆1		149336	2.84	
		3			1178.71	1178.97	M+2H$^+$	H1HN3F2NA1C-PA	◇1	816(H1HN1F1NA1)	80114	1.52	sLe^X
	pk.3-17	1	37.18	36.88-37.71	840.13<br>1259.77	840.33<br>1259.99	M+3H$^+$<br>M+2H$^+$	H2HN3F2NA1C-PA	◇1	816(H1HN1F1NA1)	387866	7.37	sLe^X
		2			1099.28	1099.44	M+2H$^+$	H2HN2F1NA1C-PA	◆1		70903	1.35	
		3			1006.29			data not available			76226	1.45	xMS2
		4			1162.72			data not available			235997	4.48	
	pk.3-18	1	38.52	38.13-39.31	1085.59	1085.43	M+2H$^+$	H2HN2F1NA1C-PA	◇1		1000432	19.01	
		2			1259.75	1259.99	M+2H$^+$	H2HN3F2NA1C-PA	◇1	816(H1HN1F1NA1)	186640	3.55	sLe^X
	pk.3-19	1	40.20	39.38-40.70	1209.00	1208.98	M+2H$^+$	H3HN3NA1C-PA	◆1		473952	9.01	
		2			1026.72	1026.41	M+2H$^+$	H2HN2NA1C-PA	◆1		460435	8.75	
		3			1259.86	1259.99	M+2H$^+$	H2HN3F2NA1C-PA	◇1	816(H1HN1F1NA1)	476961	9.06	sLe^X

Table S2A Continued.

Fr. No. (DEAE)	Peak. No. (ODS)	Full MS No.	Elusion time max (min)	Elusion time range (min)	Observed parent ion (m/z value)	Calculated (m/z value)	Estimated adduct	Estimated composition[a), b), c)]		Characteristic fragments[a)]	Area[d)]	Relative amounts[e)]	Notes
	pk.3-20	1	41.78	41.39-41.95	1119.69	1119.95	M+2H+	H1HN3F1NA1C-PA	◆1	739(HN2NA1)	255810	4.86	sLacdiNAc
		2			1261.86			data not available			104719	1.99	
	pk.3-21	1	42.55	42.15-43.20	1005.75	1005.90	M+2H+	H3HN1NA1C-PA	◆1		878630	16.70	
		2			1099.65	1099.44	M+2H+	H2HN2F1NA1C-PA	◆1		654113	12.43	
		3			1026.35	1026.41	M+2H+	H2HN2NA1C-PA	◆1		232001	4.41	
		4			1172.30	1172.47	M+2H+	H2HN2F2NA1C-PA	◆1		87550	1.66	
	pk.3-22	1	43.54	43.33-43.82	1186.54	1186.96	M+2H+	H2HN3F1NA1C-PA	◇1		145679	2.77	
		2			1354.98	1355.04	M+2H+	H3HN3F2NA1C-PA	◆1		150833	2.87	
		3			1026.18	1026.41	M+2H+	H2HN2NA1C-PA	◆1		84895	1.61	
	pk.3-23	1	44.07	43.89-44.65	925.18	924.87	M+2H+	H2HN1NA1C-PA	◆1		483832	9.19	
		2			843.73	843.85	M+2H+	H1HN1NA1C-PA	◆1		291173	5.53	
	pk.3-24	1	45.30	44.86-45.56	855.09<br>1282.19	855.01<br>1282.01	M+3H+<br>M+2H+	H3HN3F1NA1C-PA	◆1		454302	8.63	
		2			1099.12	1099.44	M+2H+	H2HN2F1NA1C-PA	◆1		135418	2.57	
		3			1187.34	1186.96	M+2H+	H2HN3F1NA1C-PA	◇1		74314	1.41	
	pk.3-25	1	46.45	46.04-46.88	684.68<br>1026.17	684.61<br>1026.41	M+3H+<br>M+2H+	H2HN2NA1C-PA	◆1		4517616	85.84	
	pk.3-26	1	47.28	46.95-47.78	782.04<br>1172.35	781.98<br>1172.47	M+3H+<br>M+2H+	H2HN2F2NA1C-PA	◆1	512(H1HN1F1)	2014618	38.28	Le[x]
	pk.3-27	1	48.22	47.85-78.40	800.81<br>1200.77	800.99<br>1200.98	M+3H+<br>M+2H+	H2HN3F1NA1C-PA	◆1		1017834	19.34	
	pk.3-28	1	48.60	48.47-49.17	806.46<br>1209.26	806.32<br>1208.98	M+3H+<br>M+2H+	H3HN3NA1C-PA	◆1		946167	17.98	
	pk.3-29	1	49.40	49.24-49.45	917.24	916.87	M+2H+	H1HN1F1NA1C-PA	◆1		172320	3.27	
	pk.3-30	1	50.22	49.65-50.70	733.38<br>1099.21	733.30<br>1099.44	M+3H+<br>M+2H+	H2HN2F1NA1C-PA	◆1		83667	1.59	
		2			1018.29	1018.41	M+2H+	H1HN2F1NA1C-PA	◆1		90396	1.72	
		3			1200.61	1200.98	M+2H+	H2HN3F1NA1C-PA	◆1		118830	2.26	
		4			922.74	922.70	M+3H+	H3HN4F1NA1C-PA	◆1		83667	1.59	
	pk.3-31	1	51.73	51.39-52.29	849.63<br>1274.35	849.68<br>1274.01	M+3H+<br>M+2H+	H2HN3F2NA1C-PA	◆1	512(H1HN1F1)	5262733	100.00	Le[x]
	pk.3-32	1	52.57	52.36-52.99	752.35<br>1127.48	752.30<br>1127.95	M+3H+<br>M+2H+	H2HN3NA1C-PA	◆1		620587	11.79	
		2			1273.78	1274.01	M+2H+	H2HN3F2NA1C-PA	◆1		136569	2.60	
	pk.3-33	1	53.34	53.20-53.89	1020.04<br>1529.48	1020.07<br>1529.60	M+3H+<br>M+2H+	H3HN4F3NA1C-PA	◆1	512(H1HN1F1)	410150	7.79	Le[x]
	pk.3-34	1	54.20	53.96-54.65	937.52	937.39	M+2H+	HN2F1NA1C-PA	◆1		208651	3.96	
	pk.3-35	1	55.57	54.93-56.25	801.58<br>1201.10	800.99<br>1200.98	M+3H+<br>M+2H+	H2HN3F1NA1C-PA	◆1		2393027	45.47	
		2			854.99<br>1282.28	855.01<br>1282.01	M+3H+<br>M+2H+	H3HN3F1NA1C-PA	◆1		109711	2.08	
		3			1120.24	1119.95	M+2H+	H1HN3F1NA1C-PA	◆1		86618	1.65	
		4			1193.03	1192.98	M+2H+	H1HN3F2NA1C-PA	◆1		120368	2.29	
	pk.3-36	1	57.81	57.43-58.27	922.63<br>1383.34	922.70<br>1383.55	M+3H+<br>M+2H+	H3HN4F1NA1C-PA	◆1		231948	4.41	
fr.4	pk.4-1	1	32.07	31.67-32.50	1160.69	1160.79	M+3H+	H3HN4F4NA2C-PA	◇2	816(H1HN1F1NA1)	354707	6.74	sLe[x]
		2			990.27	990.39	M+3H+	H2HN3F3NA2C-PA	◇2	816(H1HN1F1NA1)	243517	4.63	sLe[x]
	pk.4-2	1	33.11	32.57-33.40	1093.38			data not available			432289	8.21	
	pk.4-3	1	33.97	33.47-34.31	922.58<br>1383.91	922.70<br>1383.55	M+3H+<br>M+2H+	H2HN2F3NA2C-PA	◇2	816(H1HN1F1NA1)	785123	14.92	sLe[x]
	pk.4-4	1	37.04	36.46-37.85	990.14<br>1484.81	990.39<br>1485.09	M+3H+<br>M+2H+	H2HN3F3NA2C-PA	◇2	816(H1HN1F1NA1)	1352492	25.70	sLe[x]
	pk.4-5	1	38.82	37.92-39.38	1161.43	1160.79	M+3H+	H3HN4F4NA2C-PA	◇2	816(H1HN1F1NA1)	503482	9.57	sLe[x]
	pk.4-6	1	40.23	39.86-40.76	1069.07			data not available			281349	5.35	
	pk.4-7	1	43.43	42.92-44.17	1005.17			data not available			456633	8.68	
	pk.4-8	1	45.13	44.24-45.76	1112.06	1112.10	M+3H+	H3HN4F3NA2C-PA	◇2	816(H1HN1F1NA1)	542096	10.30	sLe[x]
	pk.4-9	1	47.46	46.94-47.50	1224.29	1224.15	M+3H+	H4HN4F4NA2C-PA	◇1◆1	816(H1HN1F1NA1)	303177	5.76	sLe[x]
	pk.4-10	1	48.36	47.64-48.96	1053.89<br>1579.78	1053.75<br>1580.13	M+3H+<br>M+2H+	H3HN3F3NA2C-PA	◇1◆1	816(H1HN1F1NA1)	780911	14.84	sLe[x]
	pk.4-11	1	49.81	49.38-50.28	1122.07	1121.45	M+3H+	H3HN4F3NA2C-PA	◇1◆1	816(H1HN1F1NA1)	396026	7.53	sLe[x]
		2			1200.24	1199.80	M+3H+	H5HN5F1NA2C-PA	◇1◆1	731(H2HN2)	222269	4.22	LacNAc repeat
	pk.4-12	1	50.85	50.35-51.67	1053.65<br>1579.73	1053.75<br>1580.13	M+3H+<br>M+2H+	H3HN3F3NA2C-PA	◇1◆1	816(H1HN1F1NA1)	379260	7.21	sLe[x]
		2			1324.85	1324.53	M+2H+	H2HN2F2NA2C-PA	◇1◆1	816(H1HN1F1NA1)	311453	5.92	sLe[x]
		3			1199.72	1199.80	M+3H+	H5HN5F1NA2C-PA	◇1◆1	1035(H2HN2NA1)	298965	5.68	sLacNAc repeat
	pk.4-13	1	52.55	51.94-52.92	1121.20	1121.45	M+3H+	H3HN4F3NA2C-PA	◇1◆1	816(H1HN1F1NA1)	111861	2.13	sLe[x]
	pk.4-14	1	53.93	53.54-54.58	951.62<br>1425.67	951.05<br>1426.07	M+3H+<br>M+2H+	H2HN3F2NA2C-PA	◇1◆1	816(H1HN1F1NA1)	710839	13.51	sLe[x]
	pk.4-15	1	55.09	54.65-55.56	1122.03<br>1681.63	1121.45<br>1681.67	M+3H+<br>M+2H+	H3HN4F3NA2C-PA	◇1◆1	816(H1HN1F1NA1)	389254	7.40	sLe[x]
	pk.4-16	1	57.63	57.22-58.06	917.45<br>1374.82	917.04<br>1375.06	M+3H+<br>M+2H+	H3HN3NA2C-PA	◆2		102017	1.94	

Table S2A Continued.

Fr. No. (DEAE)	Peak. No. (ODS)	Full MS No.	Elusion time max (min)	Elusion time range (min)	Observed parent ion (m/z value)	Calculated (m/z value)	Estimated adduct	Estimated composition[a), b), c)]		Characteristic fragments[a)]	Area[d)]	Relative amounts[e)]	Notes
	pk.4-17	1	58.54	58.19-58.89	1073.12	1072.76	M+3H$^+$	H3HN4F2NA2C-PA	◇1◆1	816(H1HN1F1NA1)	74877	1.42	sLe^X
	pk.4-18	1	59.41	59.10-60.00	1023.97	1024.08	M+3H$^+$	H3HN4F1NA2C-PA	◇1◆1		114974	2.18	
	pk.4-19	1	60.34	60.07-60.56	966.15<br>1447.87	965.73<br>1448.09	M+3H$^+$<br>M+2H$^+$	H3HN3F1NA2C-PA	◆2		25743	0.49	
fr.5	pk.5-1	1	29.09	28.75-29.24	1031.87			data not available			145496	2.76	
	pk.5-2	1	29.51	29.31-29.72	874.17<br>1310.40	874.01<br>1310.52	M+3H$^+$<br>M+2H$^+$	H2HN2F2NA2C-PA	◇2	816(H1HN1F1NA1)	169370	3.22	sLe^X
	pk.5-3	1	34.04	33.75-34.38	922.60<br>1383.05	922.70<br>1383.55	M+3H$^+$<br>M+2H$^+$	H2HN2F3NA2C-PA	◇2	816(H1HN1F1NA1)	442474	8.41	sLe^X
	pk.5-4	1	37.19	36.74-37.64	990.87<br>1485.13	990.39<br>1485.09	M+3H$^+$<br>M+2H$^+$	H2HN3F3NA2C-PA	◇2	816(H1HN1F1NA1)	220128	4.18	sLe^X
		2			925.45<br>1388.12			data not available			176011	3.34	
	pk.5-5	1	43.56	43.13-43.96	891.88<br>1337.15	892.70<br>1338.55	M+3H$^+$<br>M+2H$^+$	H2HN2F2NA2C-PA	◆2		213653	4.06	
		2			1237.46	1237.49	M+2H$^+$	H2HN2F1NA2C-PA	◇2		168341	3.20	
	pk.5-6	1	47.02	46.74-47.29	834.61<br>1251.84	834.67<br>1251.50	M+3H$^+$<br>M+2H$^+$	H2HN2F1NA2C-PA	◇1◆1		320495	6.09	
	pk.5-7	1	48.27	48.06-48.68	1227.32			data not available			163078	3.10	
		2			1178.21	1178.48	M+2H$^+$	H2HN2NA2C-PA	◇1◆1		71559	1.36	
		3			1324.86	1324.53	M+2H$^+$	H2HN2F2NA2C-PA	◇1◆1	816(H1HN1F1NA1)	52853	1.00	sLe^X
	pk.5-8	1	49.97	49.45-50.21	785.91<br>1178.37	785.99<br>1178.48	M+3H$^+$<br>M+2H$^+$	H2HN2NA2C-PA	◇1◆1		601376	11.43	
	pk.5-9	1	50.76	50.28-51.11	883.47<br>1324.78	883.36<br>1324.53	M+3H$^+$<br>M+2H$^+$	H2HN2F2NA2C-PA	◇1◆1	816(H1HN1F1NA1)	1551417	29.48	sLe^X
		2			1345.34	1345.05	M+2H$^+$	H1HN3F2NA2C-PA	◇1◆1	739(HN2NA1)<br>816(H1HN1F1NA1)	104682	1.99	sLacdiNAc<br>sLe^X
	pk.5-10	1	51.40	51.25-51.67	908.14<br>1361.53	907.70<br>1361.04	M+3H$^+$<br>M+2H$^+$	H3HN3NA2C-PA	◇1◆1		154090	2.93	
		2			1324.80	1324.53	M+2H$^+$	H2HN2F2NA2C-PA	◇1◆1	816(H1HN1F1NA1)	85890	1.63	sLe^X
	pk.5-11	1	53.06	52.71-53.47	795.43<br>1192.58	795.33<br>1192.49	M+3H$^+$<br>M+2H$^+$	H2HN2NA2C-PA	◆2		423763	8.05	
		2			1251.43	1251.50	M+2H$^+$	H2HN2F1NA2C-PA	◇1◆1		206067	3.92	
	pk.5-12	1	53.86	53.54-54.45	951.50<br>1426.28	951.05<br>1426.07	M+3H$^+$<br>M+2H$^+$	H2HN3F2NA2C-PA	◇1◆1	816(H1HN1F1NA1)	2005354	38.10	sLe^X
		2			902.78	902.36	M+3H$^+$	H2HN3F1NA2C-PA	◇1◆1		336835	6.40	
	pk.5-13	1	56.88	56.39-57.22	795.30<br>1192.45	795.33<br>1192.49	M+3H$^+$<br>M+2H$^+$	H2HN2NA2C-PA	◆2		4782824	90.88	
	pk.5-14	1	57.56	57.29-58.06	917.00<br>1374.90	917.04<br>1375.06	M+3H$^+$<br>M+2H$^+$	H3HN3NA2C-PA	◆2		1025868	19.49	
	pk.5-15	1	59.71	59.17-60.07	843.87<br>1265.38	844.02<br>1265.52	M+3H$^+$<br>M+2H$^+$	H2HN2F1NA2C-PA	◆2		938645	17.84	
	pk.5-16	1	60.48	60.14-60.97	862.94<br>1294.22	863.02<br>1294.03	M+3H$^+$<br>M+2H$^+$	H2HN3NA2C-PA	◆2		99989	1.90	
	pk.5-17	1	63.57	63.20-64.10	912.22<br>1367.33	911.71<br>1367.06	M+3H$^+$<br>M+2H$^+$	H2HN3F1NA2C-PA	◆2		456350	8.67	
fr.6	pk.6-1	1	25.71	25.14-26.11	813.07<br>1219.05	812.97<br>1218.95	M+3H$^+$<br>M+2H$^+$	H1HN3F2NA1(SO3)1C-PA	◇1	553(HN2F1)<br>816(H1HN1F1NA1)	245205	4.66	LacdiNAc(Fuc)<br>sLe^X
	pk.6-2	1	28.11	27.85-28.47	861.66<br>1292.24	861.65<br>1291.97	M+3H$^+$<br>M+2H$^+$	H1HN3F3NA1(SO3)1C-PA	◇1	553(HN2F1)<br>816(H1HN1F1NA1)	141719	2.69	LacdiNAc(Fuc)<br>sLe^X
	pk.6-3	1	30.92	30.49-31.39	861.79<br>1291.74	861.65<br>129.97	M+3H$^+$<br>M+2H$^+$	H1HN3F3NA1(SO3)1C-PA	◇1	553(HN2F1)<br>816(H1HN1F1NA1)	1368188	26.00	LacdiNAc(Fuc)<br>sLe^X
	pk.6-4	1	31.82	31.60-32.22	861.69<br>1291.88	861.65<br>1291.97	M+3H$^+$<br>M+2H$^+$	H1HN3F3NA1(SO3)1C-PA	◇1	553(HN2F1)<br>816(H1HN1F1NA1)	194903	3.70	LacdiNAc(Fuc)<br>sLe^X
	pk.6-5	1	33.50	33.13-33.75	1218.81	1218.95	M+2H$^+$	H1HN3F2NA1(SO3)1C-PA	◇1	816(H1HN1F1NA1)	95410	1.81	sLe^X
	pk.6-6	1	34.44	34.24-34.72	1218.40			data not available			80839	1.54	
	pk.6-7	1	47.26	47.01-47.57	822.66<br>1232.52	822.31<br>1232.96	M+3H$^+$<br>M+2H$^+$	H1HN3F2NA1(SO3)1C-PA	◆1	553(HN2F1)	162469	3.09	LacdiNAc(Fuc)
	pk.6-8	1	48.68	48.40-48.89	808.49<br>1212.85	808.63<br>1212.45	M+3H$^+$<br>M+2H$^+$	H2HN2F2NA1(SO3)1C-PA	◆1		126836	2.41	
	pk.6-9	1	49.19	48.96-49.51	1213.18	1212.45	M+2H$^+$	H2HN2F2NA1(SO3)1C-PA	◆1		138118	2.62	
	pk.6-10	1	50.14	49.58-50.42	822.37<br>1233.07	822.31<br>1232.96	M+3H$^+$<br>M+2H$^+$	H1HN3F2NA1(SO3)1C-PA	◆1	553(HN2F1)	243248	4.62	LacdiNAc(Fuc)
		2			835.97<br>1253.31	835.99<br>1253.47	M+3H$^+$<br>M+2H$^+$	HN4F2NA1(SO3)1C-PA	◆1	553(HN2F1)<br>739(HN2NA1)	116288	2.21	LacdiNAc(Fuc)<br>sLacdiNAc
	pk.6-11	1	54.34	54.03-54.79	889.94<br>1334.81	890.00<br>1334.50	M+3H$^+$<br>M+2H$^+$	H1HN4F2NA1(SO3)1C-PA	◆1	553(HN2F1)	90067	1.71	LacdiNAc(Fuc)
	pk.6-12	1	56.95	56.60-57.29	795.29<br>1192.85	795.33<br>1192.49	M+3H$^+$<br>M+2H$^+$	H2HN2NA2C-PA	◆2		104232	1.98	
fr.8	pk.8-1	1	13.46	13.26-13.82	777.91	778.26	M+2H$^+$	H3(HPO3)1C-PA			31209	0.59	HPO3
	pk.8-2	1	17.11	16.60-17.50	784.68	784.78	M+2H$^+$	H3(HPO3)1(MA)1C-PA			59135	1.12	HPO3+MA
	pk.8-3	1	23.07	22.57-23.40	751.83<br>1126.91	751.92<br>1127.37	M+3H$^+$<br>M+2H$^+$	HN4F2(SO3)2C-PA		553(HN2F1)	99353	1.89	LacdiNAc(Fuc)
		2			800.86<br>1200.58	800.60<br>1200.40	M+3H$^+$<br>M+2H$^+$	HN4F3(SO3)2C-PA		553(HN2F1)<br>633(HN2F1(SO3)1)	100034	1.90	LacdiNAc(Fuc, SO3)
	pk.8-4	1	26.16	25.69-26.46	1179.60			data not available			78866	1.50	xMS2
	pk.8-5	1	27.23	26.67-27.92	801.03<br>1200.28	800.60<br>1200.40	M+3H$^+$<br>M+2H$^+$	HN4F3(SO3)2C-PA		553(HN2F1)<br>633(HN2F1(SO3)1)	1324958	25.18	LacdiNAc(Fuc, SO3)
		2			751.79<br>1127.72			data not available			118687	2.26	xMS2
	pk.8-6	1	32.02	31.67-32.57	1127.54	1127.37	M+2H$^+$	HN4F2(SO3)2C-PA		553(HN2F1)	239997	4.56	LacdiNAc(Fuc)
	pk.8-7	1	32.89	32.64-33.68	1133.72			data not available			236686	4.50	
	pk.8-8	1	34.50	33.75-34.93	1133.19			data not available			149582	2.84	xMS2

Table S2A Continued.

Fr. No. (DEAE)	Peak. No. (ODS)	Full MS No.	Elusion time max (min)	Elusion time range (min)	Observed parent ion (m/z value)	Calculated (m/z value)	Estimated adduct	Estimated composition[a), b), c)]		Characteristic fragments[a)]	Area[d)]	Relative amounts[e)]	Notes
		2			1187.60			data not available			121512	2.31	xMS2
	pk.8-9	1	35.44	35.07-35.63	1194.31	1194.47	$M+3H^+$	H3HN3F4NA3C-PA	◇3	816(H1HN1F1NA1)	184199	3.50	sLe^X
	pk.8-10	1	35.88	35.76-36.25	1262.53	1262.17	$M+3H^+$	H3HN4F4NA3C-PA	◇3	816(H1HN1F1NA1)	129553	2.46	sLe^X
	pk.8-11	1	36.90	36.32-37.36	1213.59			data not available			251432	4.78	
	pk.8-12	1	37.82	37.43-38.26	1194.34	1194.47	$M+3H^+$	H3HN3F4NA3C-PA	◇3	816(H1HN1F1NA1)	206576	3.93	sLe^X
	pk.8-13	1	42.24	41.81-42.71	1262.50	1262.17	$M+3H^+$	H3HN4F4NA3C-PA	◇3	816(H1HN1F1NA1)	213406	4.06	sLe^X
	pk.8-14	1	43.26	42.85-43.89	1197.60			data not available			185594	3.53	
		2			1292.33			data not available			85268	1.62	
	pk.8-15	1	45.29	45.00-45.49	1170.25			data not available			128419	2.44	
	pk.8-16	1	48.06	47.08-48.54	1213.61	1213.48	$M+3H^+$	H3HN4F3NA3C-PA	◇3	816(H1HN1F1NA1)	124083	2.36	sLe^X
		2			1251.68	1251.50	$M+2H^+$	H2HN2F1NA2C-PA	◇1◆1	816(H1HN1F1NA1)	172723	3.28	sLe^X
	pk.8-17	1	49.50	48.89-49.65	1325.91	1325.53	$M+3H^+$	H4HN4F4NA3C-PA	◇2◆1	816(H1HN1F1NA1)	295229	5.61	sLe^X
	pk.8-18	1	50.23	49.79-50.76	1325.84	1325.53	$M+3H^+$	H4HN4F4NA3C-PA	◇2◆1	816(H1HN1F1NA1)	558866	10.62	sLe^X
	pk.8-19	1	51.42	50.90-52.08	1155.52	1155.13	$M+3H^+$	H3HN3F3NA3C-PA	◇2◆1	816(H1HN1F1NA1)	775116	14.73	sLe^X
	pk.8-20	1	52.49	52.15-52.64	1301.12	1301.18	$M+3H^+$	H5HN5F1NA3C-PA	◇2◆1	731(H2HN2)	150847	2.87	LacNAc repeat
		2			1223.10	1222.82	$M+3H^+$	H3HN4F3NA3C-PA	◇2◆1	816(H1HN1F1NA1)	177204	3.37	sLe^X
	pk.8-21	1	53.05	52.71-53.61	1155.63	1155.13	$M+3H^+$	H3HN3F3NA3C-PA	◇2◆1	816(H1HN1F1NA1)	329865	6.27	sLe^X
		2			1301.21	1301.18	$M+3H^+$	H5HN5F1NA3C-PA	◇2◆1		286513	5.44	
	pk.8-22	1	53.94	53.68-54.65	1247.31			data not available			327158	6.22	
	pk.8-23	1	55.19	54.79-55.49	1368.93			data not available			157212	2.99	
	pk.8-24	1	55.84	55.56-56.18	1368.67			data not available			147866	2.81	
	pk.8-25	1	56.70	56.25-57.15	1223.18	1222.82	$M+3H^+$	H3HN4F3NA3C-PA	◇2◆1	816(H1HN1F1NA1)	482562	9.17	sLe^X
	pk.8-26	1	59.68	59.17-60.49	1018.27<br>1527.43	1018.42<br>1527.12	$M+3H^+$<br>$M+2H^+$	H3HN3NA3C-PA	◇1◆2		384509	7.31	
		2			1174.08	1174.14	$M+3H^+$	H3HN4F2NA3C-PA	◇2◆1	816(H1HN1F1NA1)	53040	1.01	sLe^X
	pk.8-27	1	61.31	60.83-61.67	1125.56	1125.45	$M+3H^+$	H3HN4F1NA3C-PA	◇2◆1		101900	1.94	
	pk.8-28	1	62.25	61.81-62.64	1067.27	1067.10	$M+3H^+$	H3HN3F1NA3C-PA	◇1◆2		90267	1.72	
	pk.8-29	1	65.45	65.00-65.90	1027.71<br>1541.35	1027.76<br>1541.14	$M+3H^+$<br>$M+2H^+$	H3HN3NA3C-PA	◆3		134208	2.55	
		2			1183.43	1183.48	$M+3H^+$	H3HN4F2NA3C-PA	◇1◆2	816(H1HN1F1NA1)	35950	0.68	sLe^X
	pk.8-30	1	66.57	66.39-66.94				data not available			19137	0.36	
	pk.8-31	1	67.85	67.50-68.13	1076.63	1076.45	$M+3H^+$	H3HN3F1NA3C-PA	◆3		20261	0.38	
