# Supplementary material for: Structural analysis of N-glycans in chicken trachea and lung reveals potential receptors of chicken influenza viruses
Source: Sci Rep. 2022 Feb 8;12:2081. doi: 10.1038/s41598-022-05961-x (PMC8827061; doi:10.1038/s41598-022-05961-x)
Supplement: Supplementary file 6 — Supplementary Table S4. [file 41598_2022_5961_MOESM6_ESM.pdf]

Table S2B. MS and MS/MS data for PA-*N*-glycans from chicken lung after two-step alkylamidation

a) H, hexose; HN, *N*-acetylhexosamine; F, fucose (or deoxyhexose); NA, *N*-acetylneuraminic acid; SO3, sulfate group; HPO3, phosphate group; C, trimannosyl core; MA, methylamine; PA, 2-aminopyridine.

b) Sialyl linkage : ◇: α2,3; ◆ : α2,6

c) Most of the detectable fluorescence peaks eluted in 10–87 min were numbered, but not all peaks were derived from PA-*N*-glycans. The compositions of some peaks could not be determined by MS and MS/MS analysis due to insufficent signals; these cases are indicated as "data not available".

d) Individual peaks detected by fluorescence sometimes included more than two kinds of PA-glycans with different mass values. In such cases, the proportions were estimated using the ratios of integrated ion intensities for each *m/z* value detected at the corresponding elution time.

e) Amounts of glycans relative to the most abundant glycan (pk. 3-32-1), for which the amount was defined as 100.

Fr. No. (DEAE)	Peak. No. (ODS)	Full MS No.	Elusion time max (min)	Elusion time range (min)	Observed parent ion (m/z value)	Calculated (m/z value)	Estimated adduct	Estimated composition[a), b), c)]		Characteristic fragments[a)]	Area[d)]	Relative amounts[e)]	Notes
fr.3	pk.3-1	1	27.04	26.65-27.08	988.29			data not available			336924	3.36	
	pk.3-2	1	27.45	27.08-27.85	918.84<br>1377.66	918.69<br>1377.53	M+3H$^+$<br>M+2H$^+$	H4HN4NA1C-PA	◇1		675393	6.73	
		2			860.11	860.33	M+2H$^+$	H2HN2C-PA			169996	1.69	
		3			1149.48			data not available			181502	1.81	
	pk.3-3	1	28.49	27.92-28.82	796.92<br>1195.21	796.98<br>1194.96	M+3H$^+$<br>M+2H$^+$	H3HN3NA1C-PA	◇1		2622135	26.12	
	pk.3-4	1	29.22	29.03-29.58	918.70<br>1377.64	918.69<br>1377.53	M+3H$^+$<br>M+2H$^+$	H4HN4NA1C-PA	◇1		856125	8.53	
		2			1155.77	1155.91	M+2H$^+$	H3HN3F1(SO3)1C-PA			359243	3.58	
	pk.3-5	1	30.06	30.56-31.25	661.41<br>991.92	661.59<br>991.88	M+3H$^+$<br>M+2H$^+$	H3HN1NA1C-PA	◇1		1732437	17.26	
	pk.3-6	1	31.01	30.56-31.25	910.64	910.86	M+2H$^+$	H2HN1NA1C-PA	◇1		1056961	10.53	
		2			1081.92			data not available			613961	6.12	
	pk.3-7	1	31.85	31.32-32.08	1012.85	1012.40	M+2H$^+$	H2HN2NA1C-PA	◇1		552549	5.50	
		2			1061.39			data not available			870824	8.67	
		3			993.88	993.85	M+2H$^+$	H1HN3F1(SO3)1C-PA		487(HN2(SO3)1)	510569	5.09	LacdiNAc(SO3)
		4			1085.00	1085.42	M+2H$^+$	H2HN2F1NA1C-PA	◇1		493055	4.91	
	pk.3-8	1	32.44	32.15-32.57	845.83<br>1268.22	845.66<br>1267.99	M+3H$^+$<br>M+2H$^+$	H3HN3F1NA1C-PA	◇1		1268957	12.64	
		2			1085.29	1085.42	M+2H$^+$	H2HN2F1NA1C-PA	◇1		848914	8.46	
	pk.3-9	1	32.89	32.64-33.26	845.71<br>1268.86	845.66<br>1267.99	M+3H$^+$<br>M+2H$^+$	H3HN3F1NA1C-PA	◇1		1887021	18.80	
		2			973.89	973.34	M+2H$^+$	H2HN2F1(SO3)1-PA			1385535	13.80	
		3			1012.39	1012.40	M+2H$^+$	H2HN2NA1C-PA	◇1		557636	5.55	
	pk.3-10	1	34.06	33.68-34.51	675.20<br>1012.02	675.27<br>1012.40	M+3H$^+$<br>M+2H$^+$	H2HN2NA1C-PA	◇1		3189583	31.77	
	pk.3-11	1	34.88	34.58-35.42	967.49<br>1450.45	967.37<br>1450.56	M+3H$^+$<br>M+2H$^+$	H4HN4F1NA1C-PA	◇1		2301654	22.93	
	pk.3-12	1	35.80	35.56-36.11	1089.24	1089.08	M+3H$^+$	H5HN5F1NA1C-PA	◇1	731(H2HN2)<br>1035(H2HN2NA1)	515073	5.13	sLacNAc repeat
		2			983.57	983.89	M+2H$^+$	H2HN1F1NA1C-PA	◇1		467184	4.65	
		3			967.45	967.37	M+3H$^+$	H4HN4F1NA1C-PA	◇1		389897	3.88	
		4			902.66	902.86	M+2H$^+$	H1HN1F1NA1C-PA	◇1		195423	1.95	
		5			1004.78	1004.40	M+2H$^+$	H1HN2F1NA1C-PA	◇1		269224	2.68	
	pk.3-13	1	36.52	36.18-36.81	724.07<br>1085.31	723.95<br>1085.42	M+3H$^+$<br>M+2H$^+$	H2HN2F1NA1C-PA	◇1		3266063	32.53	
	pk.3-14	1	37.67	37.36-37.99	1085.34	1085.42	M+2H$^+$	H2HN2F1NA1C-PA	◇1		438857	4.37	
		2			1163.12			data not available			932557	9.29	
	pk.3-15	1	38.67	38.13-39.17	723.93<br>1085.84	723.95<br>1085.42	M+3H$^+$<br>M+2H$^+$	H2HN2F1NA1C-PA	◇1		9029898	89.95	
	pk.3-16	1	39.51	39.24-39.65	845.55<br>1267.86	845.66<br>1267.99	M+3H$^+$<br>M+2H$^+$	H3HN3F1NA1C-PA	◇1		517387	5.15	
		2			1085.13	1085.42	M+2H$^+$	H2HN2F1NA1C-PA	◇1		329337	3.28	
	pk.3-17	1	40.11	39.72-40.76	1026.79	1026.41	M+2H$^+$	H2HN2NA1C-PA	◆1		960676	9.57	
		2			1209.33	1208.98	M+2H$^+$	H3HN3NA1C-PA	◆1		783745	7.81	
		3			1267.84	1267.99	M+2H$^+$	H3HN3F1NA1C-PA	◇1		546314	5.44	
	pk.3-18	1	41.19	40.83-41.53	928.00<br>1391.48	928.03<br>1391.54	M+3H$^+$<br>M+2H$^+$	H4HN4NA1C-PA	◆1		487813	4.86	
		2			1113.52	1113.94	M+2H$^+$	H2HN3NA1C-PA	◇1		395470	3.94	
		3			967.81	967.37	M+3H$^+$	H4HN4F1NA1C-PA	◇1	1035(H2HN2NA1)<br>1096(H3HN3)	160840	1.60	sLacNAc repeat
	pk.3-19	1	41.86	41.60-42.15	845.77<br>1267.68	845.66<br>1267.99	M+3H$^+$<br>M+2H$^+$	H3HN3F1NA1C-PA	◇1		644841	6.42	
		2			1026.33	1026.41	M+2H$^+$	H2HN2NA1C-PA	◆1	739(HN2NA1)	195529	1.95	sLacdiNAc
	pk.3-20	1	43.19	42.43-43.40	1005.78	1005.90	M+2H$^+$	H3HN1NA1C-PA	◆1		1335402	13.30	
		2			1026.39	1026.41	M+2H$^+$	H2HN2NA1C-PA	◆1		1004194	10.00	
	pk.3-21	1	43.83	43.47-44.86	791.64<br>1187.31	791.65<br>1186.96	M+3H$^+$<br>M+2H$^+$	H2HN3F1NA1C-PA	◇1		1858973	18.52	
		2			924.64	924.87	M+2H$^+$	H2HN1NA1C-PA	◆1		579590	5.77	
		3			1050.27	1049.74	M+3H$^+$	H5HN5NA1C-PA	◆1		381513	3.80	
		4			844.13	843.85	M+2H$^+$	H1HN1NA1C-PA	◆1		267416	2.66	
	pk.3-22	1	45.50	44.93-46.04	976.83<br>1464.89	976.72<br>1464.57	M+3H$^+$<br>M+2H$^+$	H4HN4F1NA1C-PA	◆1		1755995	17.49	
		2			1046.85	1046.92	M+2H$^+$	H1HN3NA1C-PA	◆1	739(HN2NA1)	867922	8.65	sLacdiNAc
		3			1099.19	1099.44	M+2H$^+$	H2HN2F1NA1C-PA	◆1		969285	9.66	

Table S2B Continued.

Fr. No. (DEAE)	Peak. No. (ODS)	Full MS No.	Elusion time max (min)	Elusion time range (min)	Observed parent ion (m/z value)	Calculated (m/z value)	Estimated adduct	Estimated composition[a), b), c)]		Characteristic fragments[a)]	Area[d)]	Relative amounts[e)]	Notes
	pk.3-23	1	46.58	46.18-47.01	684.77<br>1026.53	684.61<br>1026.41	M+3H+<br>M+2H+	H2HN2NA1C-PA	◆1		9188946	91.53	
	pk.3-24	1	47.75	47.08-48.06	1098.45	1098.43	M+3H+	H5HN5F1NA1C-PA	◆1		3339847	33.27	
		2			1026.38	1026.41	M+2H+	H2HN2NA1C-PA	◆1		514824	5.13	
		3			977.20	976.72	M+3H+	H4HN4F1NA1C-PA	◆1		380421	3.79	
	pk.3-25	1	48.70	48.19-49.24	806.47<br>1209.38	806.32<br>1208.98	M+3H+<br>M+2H+	H3HN3NA1C-PA	◆1		2523727	25.14	
		2			913.42<br>1369.19	913.36<br>1369.53	M+3H+<br>M+2H+	H3HN4F1NA1C-PA	◇1		756735	7.54	
		3			1018.91	1018.41	M+2H+	H1HN2F1NA1C-PA	◆1	739(HN2NA1)	420447	4.19	sLacdiNAc
	pk.3-26	1	49.87	49.58-49.97	746.99<br>1119.92	746.97<br>1119.95	M+3H+<br>M+2H+	H1HN3F1NA1C-PA	◆1	739(HN2NA1)	2425925	24.16	sLacdiNAc
	pk.3-27	1	50.24	49.98-50.83	733.90<br>1099.38	733.30<br>1099.44	M+3H+<br>M+2H+	H2HN2F1NA1C-PA	◆1		8282363	82.50	
		2			1038.89	1038.93	M+2H+	HN3F1NA1C-PA	◆1	739(HN2NA1)	369200	3.68	sLacdiNAc
	pk.3-28	1	51.29	50.97-51.39	760.68<br>1140.38	760.65<br>1140.47	M+3H+<br>M+2H+	HN4F1NA1C-PA	◆1	739(HN2NA1)	464928	4.63	sLacdiNAc
		2			1099.70	1099.44	M+2H+	H2HN2F1NA1C-PA	◆1		99756	0.99	
		3			1120.33	1119.95	M+2H+	H1HN3F1NA1C-PA	◆1	407(HN2)	130373	1.30	LacdiNAc
	pk.3-29	1	51.73	51.56-51.84	855.16<br>1282.43	855.01<br>1282.01	M+3H+<br>M+2H+	H3HN3F1NA1C-PA	◆1		1995108	19.87	
		2			1166.29	1166.12	M+3H+	H5HN6F1NA1C-PA	◆1		598328	5.96	
	pk.3-30	1	52.61	52.29-53.33	752.30<br>1128.04	752.30<br>1127.95	M+3H+<br>M+2H+	H2HN3NA1C-PA	◆1		2404088	23.95	
	pk.3-31	1	53.73	53.50-53.77	1018.24	1018.41	M+2H+	H1HN2F1NA1C-PA	◆1		226260	2.25	
	pk.3-32	1	55.58	54.79-56.32	801.67<br>1201.41	800.99<br>1200.98	M+3H+<br>M+2H+	H2HN3F1NA1C-PA	◆1		10039087	100.00	
		2			1119.80	1119.95	M+2H+	H1HN3F1NA1C-PA	◆1		450567	4.49	
		3			873.99<br>1310.22	874.01<br>1310.52	M+3H+<br>M+2H+	H3HN4NA1C-PA	◆1		162413	1.62	
		4			855.11<br>1281.75	855.01<br>1282.01	M+3H+<br>M+2H+	H3HN3F1NA1C-PA	◆1		565005	5.63	
	pk.3-33	1	56.74	56.53-57.15	1200.80	1200.98	M+2H+	H2HN3F1NA1C-PA	◆1		224258	2.23	
		2			976.92	976.72	M+3H+	H4HN4F1NA1C-PA	◆1		84749	0.84	
		3			814.50<br>1221.48	814.66<br>1221.49	M+2H+	H1HN4F1NA1C-PA	◆1	407(HN2)	151454	1.51	LacdiNAc
	pk.3-34	1	57.82	57.36-58.40	922.69<br>1383.91	922.70<br>1383.55	M+3H+<br>M+2H+	H3HN4F1NA1C-PA	◆1		2007650	20.00	
	pk.3-35	1	60.35	60.07-60.67	922.96<br>1383.78	922.70<br>1383.55	M+3H+<br>M+2H+	H3HN4F1NA1C-PA	◆1		245436	2.44	
	pk.3-36	1	62.29	62.00-62.64	1044.85	1044.41	M+3H+	H4HN5F1NA1C-PA	◆1		183465	1.83	
fr.4	pk.4-1	1	29.20	28.82-29.44	1151.93	1151.43	M+2H+	H2HN2NA2C-PA			150854	1.50	non-alkylamidated NA
	pk.4-2	1	33.03	32.36-33.54	898.29<br>1346.79	898.35<br>1347.03	M+3H+<br>M+2H+	H3HN3NA2C-PA	◇2		869785	8.66	
	pk.4-3	1	34.11	33.75-34.51	1308.25	1307.97	M+2H+	H3HN3F1NA1(SO3)1C-PA	◇1		493056	4.91	
	pk.4-4	1	37.85	37.08-38.82	947.57<br>1420.09	947.04<br>1420.05	M+3H+<br>M+2H+	H3HN3F1NA2C-PA	◇2		1092384	10.88	
		2			1125.69	1125.40	M+2H+	H2HN2F1NA1(SO3)1C-PA	◇1		664713	6.62	
		3			1164.85	1164.46	M+2H+	H2HN2NA2C-PA	◇2		575451	5.73	
	pk.4-5	1	40.29	39.38-41.25	1068.57	1068.75	M+3H+	H4HN4F1NA2C-PA	◇2	731(H2HN2)	1143078	11.39	LacNAc repeat
		2			1190.66	1190.46	M+3H+	H5HN5F1NA2C-PA	◇2	1035(H2HN2NA1)	546361	5.44	sLacNAc repeat
		3			1312.14	1312.17	M+3H+	H6HN6F1NA2C-PA	◇2	731(H2HN2)<br>1035(H2HN2NA1)	353762	3.52	sLacNAc repeat
	pk.4-6	1	41.91	41.39-42.22	1154.55			data not available			367025	3.66	
		2			1312.09	1312.17	M+3H+	H6HN6F1NA2C-PA	◇2	731(H2HN2)<br>1035(H2HN2NA1)	308151	3.07	sLacNAc repeat
	pk.4-7	1	42.56	42.36-43.06	1154.55			data not available			328339	3.27	
		2			1237.39	1237.49	M+2H+	H2HN2F1NA2C-PA	◇2		203693	2.03	
	pk.4-8	1	43.64	43.19-44.10	825.34<br>1237.20	825.33<br>1237.49	M+3H+<br>M+2H+	H2HN2F1NA2C-PA	◇2		1495736	14.90	
	pk.4-9	1	44.55	44.17-45.00	947.36<br>1419.66	947.04<br>1420.05	M+3H+<br>M+2H+	H3HN3F1NA2C-PA	◇2	731(H2HN2)<br>1035(H2HN2NA1)	590201	5.88	sLacNAc repeat
	pk.4-10	1	45.77	45.42-46.11	909.61<br>1361.43	907.70<br>1361.04	M+4H+<br>M+3H+	H3HN3NA2C-PA	◇1◆1		623116	6.21	
	pk.4-11	1	46.25	46.18-47.10	1151.20	1151.12	M+3H+	H5HN5NA2C-PA	◇1◆1		298018	2.97	
		2			947.09<br>1419.77	947.04<br>1420.05	M+3H+<br>M+2H+	H3HN3F1NA2C-PA	◇2		383478	3.82	
	pk.4-12	1	47.73	47.36-47.92	1029.65	1029.41	M+3H+	H4HN4NA2C-PA	◇1◆1		436947	4.35	
	pk.4-13	1	48.41	48.06-48.82	1227.45			data not available			367381	3.66	
		2			1078.10	1078.09	M+3H+	H4HN4F1NA2C-PA	◇1◆1		263408	2.62	
		3			1151.20	1151.12	M+3H+	H5HN5NA2C-PA	◇1◆1		235859	2.35	
	pk.4-14	1	49.70	49.10-50.42	900.64<br>1199.96	900.10<br>1199.80	M+4H+<br>M+3H+	H5HN5F1NA2C-PA	◇1◆1		1166149	11.62	
		2			1078.36<br>1616.45	1078.09<br>1616.64	M+3H+<br>M+2H+	H4HN4F1NA2C-PA	◇1◆1		291713	2.91	
		3			786.00<br>1178.43	785.99<br>1178.48	M+3H+<br>M+2H+	H2HN2NA2C-PA	◇1◆1		959891	9.56	
		4			956.39	956.38	M+3H+	H3HN3F1NA2C-PA	◇1◆1		186048	1.85	

Table S2B Continued.

Fr. No. (DEAE)	Peak. No. (ODS)	Full MS No.	Elusion time max (min)	Elusion time range (min)	Observed parent ion (m/z value)	Calculated (m/z value)	Estimated adduct	Estimated composition[a), b), c)]		Characteristic fragments[a)]	Area[d)]	Relative amounts[e)]	Notes
	pk.4-15	1	50.85	50.49-51.25	1200.22	1199.80	$M+3H^+$	H5HN5F1NA2C-PA	◇1◆1		1582248	15.76	
		2			1321.96	1321.51	$M+3H^+$	H6HN6F1NA2C-PA	◇1◆1	731(H2HN2)<br>1035(H2HN2NA1)	456006	4.54	sLacNAc repeat
		3			1078.10	1078.09	$M+3H^+$	H4HN4F1NA2C-PA	◇1◆1		306247	3.05	
	pk.4-16	1	51.41	51.32-51.67	907.53<br>1360.89	907.70<br>1361.04	$M+3H^+$<br>$M+2H^+$	H3HN3NA2C-PA	◇1◆1		445538	4.44	
	pk.4-17	1	52.19	51.81-52.50	794.80<br>1191.73	795.33<br>1192.49	$M+3H^+$<br>$M+2H^+$	H2HN2NA2C-PA	◆2		682556	6.80	
	pk.4-18	1	53.17	52.64-53.68	834.96<br>1251.42	834.67<br>1251.50	$M+3H^+$<br>$M+2H^+$	H2HN2F1NA2C-PA	◇1◆1		1418302	14.13	
		2			795.45<br>1192.31	795.23<br>1192.49	$M+3H^+$<br>$M+2H^+$	H2HN2NA2C-PA	◆2		482551	4.81	
		3			1180.59	1180.45	$M+2H^+$	HN4F1NA1(SO3)1C-PA	◆1	739(HN2NA1)	181366	1.81	sLacdiNAc
	pk.4-19	1	54.16	53.75-54.93	956.88<br>1434.28	956.38<br>1434.07	$M+3H^+$<br>$M+2H^+$	H3HN3F1NA2C-PA	◇1◆1		875037	8.72	
		2			853.74<br>1280.13	853.68<br>1280.01	$M+3H^+$<br>$M+2H^+$	H2HN3NA2C-PA	◇1◆1		182696	1.82	
		3			1145.82			data not available			90035	0.90	
		4			1267.44	1267.50	$M+3H^+$	H5HN6F1NA2C-PA	◇1◆1		59421	0.59	
	pk.4-20	1	56.93	56.32-57.22	795.37<br>1192.30	795.33<br>1192.49	$M+3H^+$<br>$M+2H^+$	H2HN2NA2C-PA	◆2		4620366	46.02	
	pk.4-21	1	57.59	57.36-58.06	916.99<br>1375.89	917.04<br>1375.06	$M+3H^+$<br>$M+2H^+$	H3HN3NA2C-PA	◆2		1105803	11.01	
		2			1192.65	1192.49	$M+2H^+$	H2HN2NA2C-PA	◆2		247442	2.46	
		3			1352.55	1353.04	$M+2H^+$	H2HN3F1NA2C-PA	◇1◆1		178717	1.78	
	pk.4-22	1	59.74	59.24-60.07	844.57<br>1265.42	844.02<br>1265.52	$M+3H^+$<br>$M+2H^+$	H2HN2F1NA2C-PA	◆2		2000645	19.93	
		2			1024.20	1024.08	$M+3H^+$	H3HN4F1NA2C-PA	◇1◆1		167010	1.66	
	pk.4-23	1	60.40	60.14-60.90	965.73<br>1448.10	965.73<br>1448.09	$M+3H^+$<br>$M+2H^+$	H3HN3F1NA2C-PA	◆2		312994	3.12	
		2			843.84<br>1265.86	844.02<br>1265.52	$M+3H^+$<br>$M+2H^+$	H2HN2F1NA2C-PA	◆2		141544	1.41	
		3			1306.93	1306.55	$M+2H^+$	HN4F1NA2C-PA	◆2	739(HN2NA1)	73372	0.73	sLacdiNAc
		4			1285.92	1286.03	$M+2H^+$	H1HN3F1NA2C-PA	◆2	739(HN2NA1)	57559	0.57	sLacdiNAc
		5			1294.22	1294.03	$M+2H^+$	H2HN3NA2C-PA	◆2		44984	0.45	
	pk.4-24	1			911.74<br>1366.84	911.71<br>1367.06	$M+3H^+$<br>$M+2H^+$	H2HN3F1NA2C-PA	◆2		666601	6.64	
		2			1145.82	1145.79	$M+3H^+$	H4HN5F1NA2C-PA	◇1◆1	1002(H1HN1NA2)	45148	0.45	α2,3, α2,6NA LacNAc
	pk.4-25	1			1033.59	1033.42	$M+3H^+$	H3HN4F1NA2C-PA	◆2		183546	1.83	
fr.5	pk.5-1	1	12.56	11.99-12.96	777.97	778.26	$M+2H^+$	H3(HPO3)1C-PA			145388	1.45	
	pk.5-2	1	13.54	13.09-14.06	778.03	778.26	$M+2H^+$	H3(HPO3)1C-PA			211914	2.11	
	pk.5-3	1	16.99	16.40-17.51	784.70	784.78	$M+2H^+$	H3(HPO3)1(MA)1C-PA			368720	3.67	HPO3+MA
	pk.5-4	1	17.95	17.64-18.26	778.16	778.26	$M+2H^+$	H3(HPO3)1C-PA			59735	0.60	
	pk.5-5	1	18.53	18.33-19.02	784.47	784.78	$M+2H^+$	H3(HPO3)1(MA)1C-PA			77098	0.77	HPO3+MA
	pk.5-6	1	27.42	27.15-27.77	797.49<br>1195.99			data not available			121461	1.21	
	pk.5-7	1	31.49	31.36-31.91	1013.84	1013.32	$M+2H^+$	H2HN2F1(SO3)2C-PA			178979	1.78	
	pk.5-8	1	36.62	36.39-36.94	1145.48			data not available			45259	0.45	
		2			1276.30			data not available			51403	0.51	
	pk.5-9	1	37.49	37.08-37.98	1000.10	999.73	$M+3H^+$	H3HN3NA3C-PA	◇3		243496	2.43	
	pk.5-10	1	38.85	38.46-39.35	1124.90	1125.40	$M+2H^+$	H2HN2F1NA1(SO3)1C-PA	◇1		237920	2.37	
	pk.5-11	1	39.88	39.42-40.32	1121.98			data not available			228522	2.28	
	pk.5-12	1	43.03	42.52-43.76	1048.69	1048.41	$M+3H^+$	H3HN3F1NA3C-PA	◇3		174766	1.74	
		2			1291.81	1291.84	$M+3H^+$	H5HN5F1NA3C-PA	◇3		233822	2.33	
	pk.5-13	1	44.96	43.83-45.90	1170.14	1170.13	$M+3H^+$	H4HN4F1NA3C-PA	◇3		570356	5.68	
		2			1291.96	1291.84	$M+3H^+$	H5HN5F1NA3C-PA	◇3	731(H2HN2)<br>1035(H2HN2NA1)	238236	2.37	sLacNAc repeat
	pk.5-14	1	47.55	46.93-48.18	1066.46			data not available			364317	3.63	
	pk.5-15	1	49.35	48.73-49.62	1048.33			data not available			375897	3.74	
	pk.5-16	1	50.13	49.76-50.45	1252.38	1252.49	$M+3H^+$	H5HN5NA3C-PA	◇2◆1		289273	2.88	
		2			1009.61			data not available			80314	0.80	
	pk.5-17	1	50.98	50.52-51.21	1252.20	1252.49	$M+3H^+$	H5HN5NA3C-PA	◇2◆1		139983	1.39	
		2			1301.41	1301.18	$M+3H^+$	H5HN5F1NA3C-PA	◇2◆1		125654	1.25	
		3			1131.29			data not available			108418	1.08	
		4			1199.74			data not available			109015	1.09	
	pk.5-18	1	51.55	51.41-51.69	1226.48			data not available			218971	2.18	
	pk.5-19	1	51.89	51.76-52.17	1180.61	1179.47	$M+3H^+$	H4HN4F1NA3C-PA	◇2◆1		107021	1.07	
		2			1422.53			data not available			73011	0.73	

Table S2B Continued.

Fr. No. (DEAE)	Peak. No. (ODS)	Full MS No.	Elusion time max (min)	Elusion time range (min)	Observed parent ion (m/z value)	Calculated (m/z value)	Estimated adduct	Estimated composition[a), b), c)]		Characteristic fragments[a)]	Area[d)]	Relative amounts[e)]	Notes
	pk.5-20	1	52.62	52.21-52.86	976.16<br>1301.74	976.14<br>1301.18	$M+4H^+$<br>$M+3H^+$	H5HN5F1NA3C-PA	◇2◆1		934188	9.31	
	pk.5-21	1	53.50	53.00-54.24	1301.47	1301.18	$M+3H^+$	H5HN5F1NA3C-PA	◇2◆1		913536	9.10	
		2			1179.58	1179.47	$M+3H^+$	H4HN4F1NA3C-PA	◇2◆1		190455	1.90	
		3			1247.11	1247.16	$M+3H^+$	H4HN5F1NA3C-PA	◇2◆1		113429	1.13	
	pk.5-22	1	55.27	54.93-55.55	1369.06	1368.87	$M+3H^+$	H5HN6F1NA3C-PA	◇2◆1		219335	2.18	
	pk.5-23	1	56.63	56.31-56.86	1058.09	1057.76	$M+3H^+$	H3HN3F1NA3C-PA	◇2◆1		246815	2.46	
	pk.5-24	1	59.71	59.20-60.17	1018.20<br>1527.37	1018.42<br>1527.12	$M+3H^+$<br>$M+2H^+$	H3HN3NA3C-PA	◇1◆2		573784	5.72	
	pk.5-25	1	61.42	61.06-61.68	1125.87	1125.45	$M+3H^+$	H3HN4F1NA3C-PA	◇2◆1		128420	1.28	
	pk.5-26	1	62.28	62.03-62.58	1067.56<br>1599.97	1067.10<br>1600.15	$M+3H^+$<br>$M+2H^+$	H3HN3F1NA3C-PA	◇1◆2		173188	1.73	
	pk.5-27	1	65.45	65.13-65.89	1028.23<br>1540.82	1027.76<br>1541.14	$M+3H^+$<br>$M+2H^+$	H3HN3NA3C-PA	◆3		185786	1.85	
	pk.5-28	1	66.52	66.30-66.78	1135.45	1134.80	$M+3H^+$	H3HN4F1NA3C-PA	◇1◆2		63779	0.64	
	pk.5-29	1	67.95	67.68-68.30	1077.19<br>1614.25	1076.45<br>1614.17	$M+3H^+$<br>$M+2H^+$	H3HN3F1NA3C-PA	◆3		51232	0.51	
fr.6	pk.6-1	1	33.81	33.29-34.18	1054.13	1054.34	$M+2H^+$	HN4F1(SO3)2C-PA		487(HN2(SO3)1)	597615	5.95	LacdiNAc(SO3)
	pk.6-2	1	38.74	38.11-39.56	973.70<br>1459.65			data not available			280002	2.79	
	pk.6-3	1	44.07	43.69-44.45	1277.54	1277.47	$M+2H^+$	H2HN2F1NA2(SO3)1C-PA	◇2		192815	1.92	
	pk.6-4	1	47.48	46.93-47.97	1069.15			data not available			241674	2.41	
	pk.6-5	1	48.87	48.17-49.21	1271.79	1271.50	$M+3H^+$	H4HN4F1NA4C-PA	◇4		234573	2.34	
	pk.6-6	1	49.88	49.48-50.24	988.35			data not available			466318	4.65	
	pk.6-7	1	51.25	50.79-51.55	907.04			data not available			279781	2.79	
	pk.6-8	1	53.37	52.93-53.76	1061.12			data not available			439399	4.38	
	pk.6-9	1	54.44	53.96-54.58	1291.23	1291.48	$M+2H^+$	H2HN2F1NA2(SO3)1C-PA	◇1◆1		98759	0.98	
		2			1143.34<br>1524.47	1143.45<br>1524.27	$M+4H^+$<br>$M+3H^+$	H6HN6F1NA4C-PA	◇3◆1	731(H2HN2)	229742	2.29	LacNAc repeat
	pk.6-10	1	54.91	53.96-54.58	1402.71	1402.56	$M+3H^+$	H5HN5F1NA4C-PA	◇3◆1		446013	4.44	
	pk.6-11	1	55.63	55.48-55.82	1402.74	1402.56	$M+3H^+$	H5HN5F1NA4C-PA	◇3◆1	731(H2HN2)	150863	1.50	LacNAc repeat
	pk.6-12	1	57.90	57.48-58.17	1232.46			data not available			158026	1.57	
	pk.6-13	1	60.86	60.65-61.20	1305.38	1305.50	$M+2H^+$	H2HN2F1NA2(SO3)1C-PA	◆2		159184	1.59	
