# Supplementary material for: Structural analysis of N-glycans in chicken trachea and lung reveals potential receptors of chicken influenza viruses
Source: Sci Rep. 2022 Feb 8;12:2081. doi: 10.1038/s41598-022-05961-x (PMC8827061; doi:10.1038/s41598-022-05961-x)
Supplement: Supplementary file 7 — Supplementary Table S5. [file 41598_2022_5961_MOESM7_ESM.pdf]

Table S3. Lists of detected PA-*N*-glycans from chicken trachea (left) and lung (right)

[a)] Monosaccharide symbols for deduced glycan structures were as follows:
: Man : Gal : Glc : GlcNAc : GalNAc : Fuc : NeuAc : sulfate group : phosphate group

[b)] Amounts of glycans relative to the most abundant glycan (pk. 1-9-1 of trachea, pk.1-28-1 of lung), for which the amount was defined as 100.

[c)] Linkages of sialic acids (α2,3, α2,6) were deduced based on the elution positions and the results of SALSA and/or SALSA/permethylation. Sia-linkages were not indicated, when they could not been deduced unambigously.

trachea

No.	Peak. No. (ODS)	Deduced glycan structure[a)]	Relative amounts[b)]	Notes[c)]	Presence in lung (No. in lung)
1T	pk.1-9-1	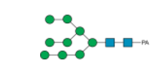	100.00		(5L)
2T	pk.1-24-1	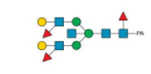	87.51	Le^X, Le^X	(132L)
3T	pk.1-12-1	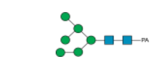	87.43		(4L)
4T	pk.1-7-1	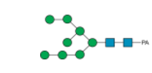	78.84		(6L)
5T	pk.3-26-1	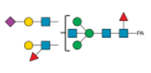	73.24	Le^X α2,6-Sia	
6T	pk.1-14-1	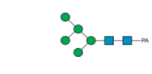	70.14		(2L)
7T	pk.3-16-1	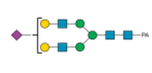	64.01	Standard a α2,6-Sia	(11L)
8T	pk.5-4-1 pk.6-9-1	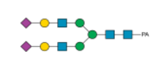	55.46	Standard A α2,6-Sia, α2,6-Sia	(16L)
9T	pk.1-33-1	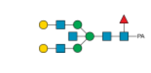	43.37	Standard H	(3L)
10T	pk.3-14-1	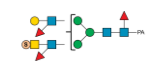	35.75	Le^X, LacdiNAc with Fuc and SO3	
11T	pk.1-8-1	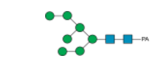	35.46		(15L)
12T	pk.5-11-1 pk.4-13-1	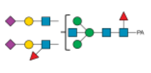	32.08	sLe^X α2,6-Sia	
13T	pk.1-26-1	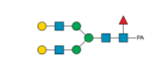	28.77	Standard F	(1L)
14T	pk.3-30-1	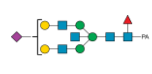	28.59	Standard g α2,6-Sia	(8L)
15T	pk.1-23-1	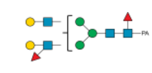	28.21	Le^X	(145L)
16T	pk.1-29-1	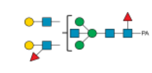	25.14	Le^X	
17T	pk.1-20-1	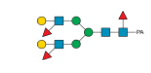	24.64	Le^X	
18T	pk.4-12-1 pk.5-10-1	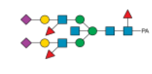	23.44	sLe^X	

lung

No.	Peak. No. (ODS)	Deduced glycan structure[a)]	Relative amounts[b)]	Notes[c)]	Presence in trachea (No. in trachea)
1L	pk.1-28-1	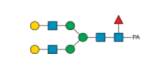	100.00	Standard F	(13T)
2L	pk.1-14-1	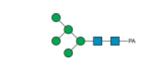	96.71		(6T)
3L	pk.1-38-1	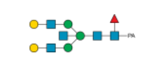	78.27	Standard H	(9T)
4L	pk.1-11-1	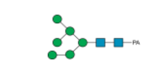	49.30		(3T)
5L	pk.1-8-1	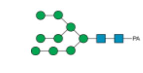	43.67		(1T)
6L	pk.1-6-1	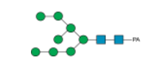	42.95		(4T)
7L	pk.3-30-1	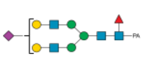	38.12	α2,3-Sia	
8L	pk.3-35-1	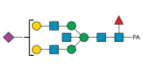	37.34	Standard g α2,6-Sia	(14T)
9L	pk.1-22-1	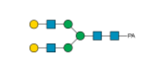	32.74	Standard E	(28T)
10L	pk.1-17-1	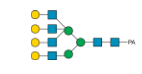	30.68		
11L	pk.3-17-1	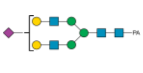	29.45	Standard a α2,6-Sia	(7T)
12L	pk.1-15-1	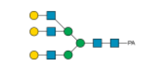	26.83		
13L	pk.3-26-1	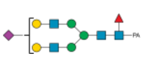	26.14	Standard e α2,6-Sia	(48T)
14L	pk.1-23-1	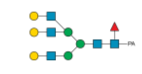	25.53		
15L	pk.1-7-1	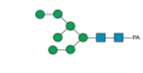	23.81		(11T)
16L	pk.4-15-1	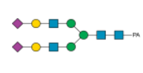	20.94	Standard A α2,6-Sia, α2,6-Sia	(8T)
17L	pk.1-27-1	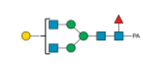	20.52		
18L	pk.1-15-2	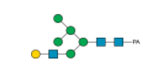	19.28		(43T)

Table S3 Continued.

No.	Peak. No. (ODS)	Deduced glycan structure[a]	Relative amounts[b]		Notes[c]	Presence in lung (No. in lung)
19T	pk.1-13-1	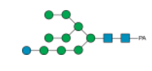	22.42			(24L)
20T	pk.4-10-1<br>pk.5-6-1	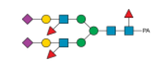	21.47		sLe^X, sLe^X	
21T	pk.1-28-1	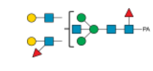	21.41		Le^X	
22T	pk.1-25-1	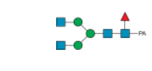	20.75		Standard J	(34L)
23T	pk.1-17-1	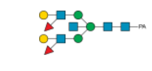	20.02		Le^X, Le^X	
24T	pk.3-24-1	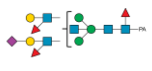	19.02		Le^X, sLe^X	
25T	pk.3-19-1	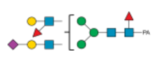	18.75		Le^X<br>α2,6-Sia	
26T	pk.1-11-1	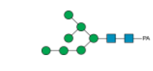	18.31			(26L)
27T	pk.6-8-1<br>pk.5-3-1	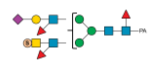	18.27		sLe^X,<br>LacdiNAc with Fuc and SO3	
28T	pk.1-21-1	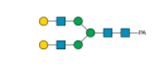	16.86		Standard E	(9L)
29T	pk.1-27-1	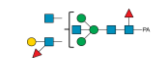	16.67		Le^X	
30T	pk.1-22-1	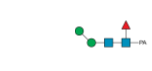	16.44			(29L)
31T	pk.1-22-2	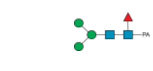	16.15			(37L)
32T	pk.8-5-1	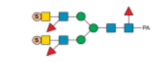	14.87		LacdiNAc with Fuc and SO3,<br>LacdiNAc with Fuc and SO3	
33T	pk.5-7-1	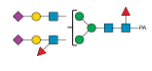	13.76		sLe^X<br>α2,6-Sia	
34T	pk.3-18-1	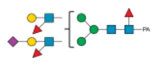	13.73		Le^X, sLe^X	
35T	pk.1-11-2	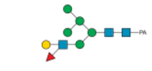	13.72		Le^X	
36T	pk.3-17-1	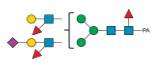	12.44		Le^X, sLe^X	
37T	pk.1-28-2	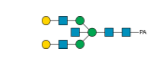	12.39		Standard G	(19L)
38T	pk.5-9-1	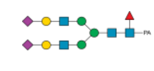	12.32		Standard B<br>α2,6-Sia, α2,6-Sia	(25L)

No.	Peak. No. (ODS)	Deduced glycan structure[a]	Relative amounts[b]		Notes[c]	Presence in trachea (No. in trachea)
19L	pk.1-32-1	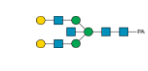	17.35		Standard G	(37T)
20L	pk.1-24-1	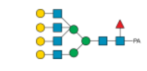	17.25			
21L	pk.3-25-1	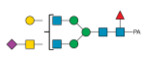	13.15		sLacdiNAc with α2,6-Sia	
22L	pk.1-37-1	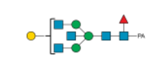	13.09			(39T)
23L	pk.1-36-1	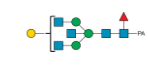	11.86			(40T)
24L	pk.1-13-1	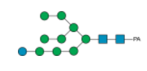	9.83			(19T)
25L	pk.4-20-1	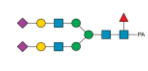	9.00		Standard B<br>α2,6-Sia, α2,6-Sia	(39T)
26L	pk.1-10-1	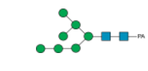	8.92			(26T)
27L	pk.1-16-1	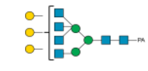	8.42			
28L	pk.3-27-1	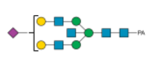	7.99		α2,6-Sia	(61T)
29L	pk.1-23-2	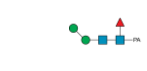	7.20			(30T)
30L	pk.1-15-3	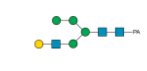	7.19			
31L	pk.4-25-1	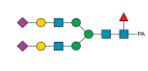	7.11		α2,3-Sia, α2,3-Sia	(89T)
32L	pk.1-30-1	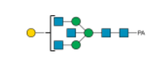	7.05			
33L	pk.1-20-2	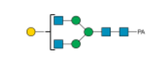	7.00			
34L	pk.1-26-1	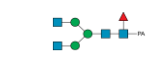	6.95		Standard J	(22T)
35L	pk.3-22-1	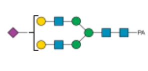	6.54		Standard c<br>α2,3-Sia	(46T)
36L	pk.4-19-1	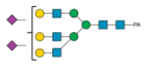	6.37			
37L	pk.1-23-4	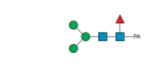	6.21			(31T)
38L	pk.1-24-2	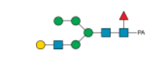	5.96			

Table S3 Continued.

No.	Peak. No. (ODS)	Deduced glycan structure[a]	Relative amounts[b]		Notes[c]	Presence in lung (No. in lung)
39T	pk.1-32-1	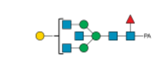	11.99			(22L)
40T	pk.1-31-1	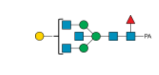	11.23			(23L)
41T	pk.3-27-1	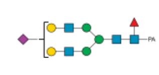	10.78		α2,3-Sia	
42T	pk.3-9-1	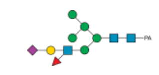	10.58		sLe^X	
43T	pk.1-16-1	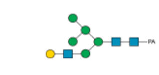	10.25			(18L)
44T	pk.5-8-1	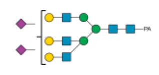	10.18		α2,6-Sia, α2,6-Sia	(36L or 141L)
45T	pk.3-2-1	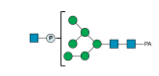	10.04		HPO3	(78L)
46T	pk.3-20-1	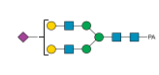	9.76		Standard c<br>α2,3-Sia	(35L)
47T	pk.1-30-1	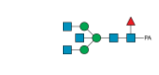	9.12		Standard L	(41L)
48T	pk.3-25-1	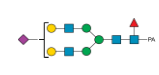	8.64		α2,6-Sia	(13L)
49T	pk.3-21-1	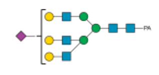	8.38		α2,6-Sia	(45L)
50T	pk.1-19-1	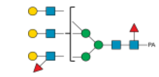	7.99		Le^X	(144L)
51T	pk.3-10-1	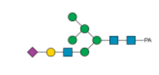	7.95		α2,6-Sia	(66L)
52T	pk.1-27-3	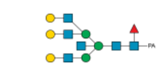	7.71			(80L)
53T	pk.4-14-1	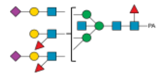	7.41		Le^X, sLe^X<br>α2,6-Sia	
54T	pk.3-11-1	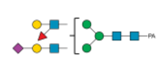	7.12		Le^X<br>α2,6-Sia	
55T	pk.3-28-1	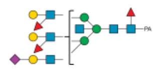	7.08		Le^X, Le^X<br>α2,6-Sia	
56T	pk.1-10-1	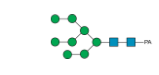	7.04			(60L)
57T	pk.3-20-2	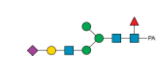	6.78		α2,6-Sia	(70L)
58T	pk.3-16-2	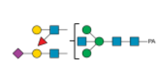	6.30		Le^X<br>α2,6-Sia	

No.	Peak. No. (ODS)	Deduced glycan structure[a]	Relative amounts[b]		Notes[c]	Presence in trachea (No. in trachea)
39L	pk.3-41-1	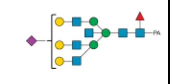	5.86		α2,6-Sia	(84T)
40L	pk.3-31-1	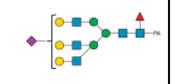	5.80		α2,6-Sia	
41L	pk.1-35-1	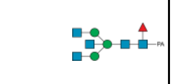	5.65		Standard L	(47T)
42L	pk.3-39-1	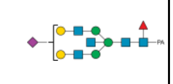	5.59		α2,3-Sia	(63T)
43L	pk.1-25-1	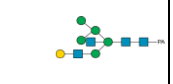	5.18			
44L	pk.4-26-1	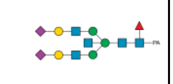	5.09		Standard D<br>α2,6-Sia, α2,6-Sia	(59T)
45L	pk.3-23-3	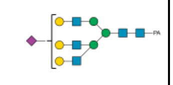	4.88		α2,6-Sia	(49T)
46L	pk.1-23-3	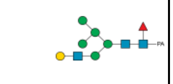	4.54			
47L	pk.3-20-1	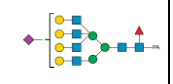	4.37		α2,6-Sia	
48L	pk.4-22-1	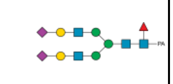	4.35		α2,3-Sia, α2,6-Sia	
49L	pk.1-28-2	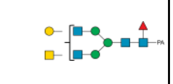	4.28		LacdiNAc	
50L	pk.1-33-1	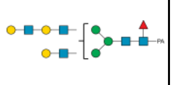	4.24		LacNAc repeat	
51L	pk.3-29-1	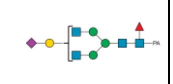	4.19		α2,3-Sia	
52L	pk.3-12-1	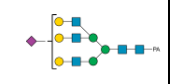	4.15		α2,3-Sia	
53L	pk.5-1-1	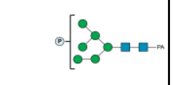	4.11		HPO3	(87T)
54L	pk.4-5-1	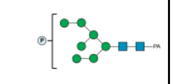	4.11		HPO3	(111T)
55L	pk.1-29-1	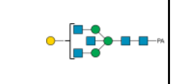	4.10			(81T)
56L	pk.1-20-3	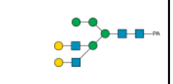	4.10			
57L	pk.3-23-4	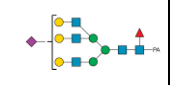	4.08		α2,3-Sia	
58L	pk.3-26-2	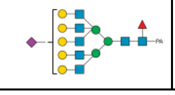	3.99		α2,6-Sia	

Table S3 Continued.

No.	Peak. No. (ODS)	Deduced glycan structure[a]	Relative amounts[b]	Notes[c]	Presence in lung (No. in lung)
59T	pk.5-13-1	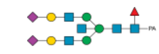	6.27	Standard D<br>α2,6-Sia, α2,6-Sia	(44L)
60T	pk.3-15-1	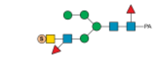	6.20	LacdiNAc with Fuc and SO3	
61T	pk.3-26-2	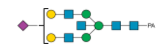	6.02	α2,6-Sia	(28L)
62T	pk.3-25-2	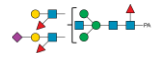	5.81	Le[X], sLe[X]	
63T	pk.3-32-1	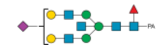	5.50	α2,3-Sia	(42L)
64T	pk.4-11-1	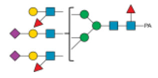	5.39	Le[X], sLe[X]<br>α2,6-Sia	
65T	pk.8-11-1	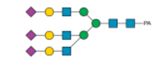	4.67	α2,6-Sia,<br>α2,6-Sia,<br>α2,6-Sia	
66T	pk.5-5-1	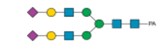	4.66	α2,3-Sia, α2,6-Sia	(86L)
67T	pk.3-11-2	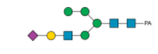	4.60	α2,6-Sia	(119L)
68T	pk.3-29-1	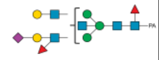	4.58	sLe[X]	
69T	pk.1-18-1	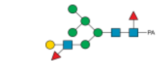	4.36	Le[X]	
70T	pk.3-3-1	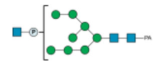	4.30	HPO3	(97L)
71T	pk.3-26-3	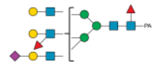	4.23	Le[X]<br>α2,6-Sia	
72T	pk.3-7-1	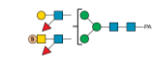	4.14	LacdiNAc with Fuc and SO3,<br>Le[X]	
73T	pk.5-2-1	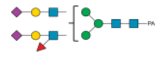	4.00	sLe[X]<br>α2,6-Sia	
74T	pk.3-6-2	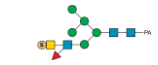	3.97	LacdiNAc with Fuc and SO3	
75T	pk.8-15-1	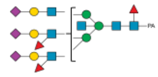	3.84	sLe[X], sLe[X]<br>α2,6-Sia	
76T	pk.3-4-1	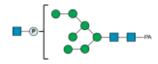	3.67	HPO3	(138L)
77T	pk.3-13-1	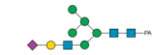	3.47	α2,3-Sia	(61L)
78T	pk.3-29-2	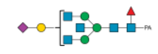	3.47	α2,6-Sia	(62L)

No.	Peak. No. (ODS)	Deduced glycan structure[a]	Relative amounts[b]	Notes[c]	Presence in trachea (No. in trachea)
59L	pk.1-19-1	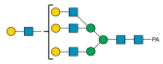	3.86	LacNAc repeat	
60L	pk.1-9-1	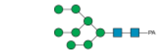	3.80		(56T)
61L	pk.3-13-1	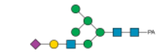	3.79	α2,3-Sia	(77T)
62L	pk.3-34-1	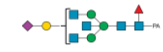	3.72	α2,6-Sia	(78T)
63L	pk.1-26-2	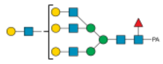	3.68	LacNAc repeat	
64L	pk.4-20-2	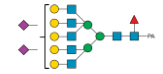	3.63		
65L	pk.1-24-3	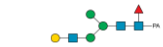	3.61		
66L	pk.3-10-1	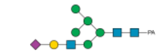	3.59	α2,6-Sia	(51T)
67L	pk.3-18-1	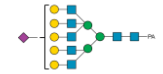	3.56	α2,6-Sia	
68L	pk.3-28-1	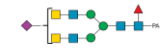	3.54	LacdiNAc,<br>sLacdiNAc with α2,6-Sia	
69L	pk.1-31-1	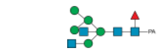	3.53		
70L	pk.3-21-1	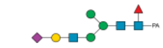	3.40	α2,6-Sia	(57T)
71L	pk.3-33-1	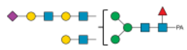	3.34	sLacNAc repeat with α2,3-Sia	
72L	pk.1-10-2	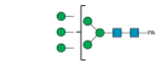	3.33		
73L	pk.3-29-2	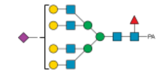	3.32	α2,3-Sia	
74L	pk.3-19-1	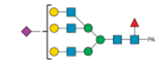	3.27	α2,3-Sia	
75L	pk.4-24-1	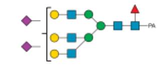	3.24		
76L	pk.1-41-1	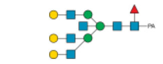	3.15		
77L	pk.3-41-2	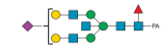	3.12	α2,3-Sia	(95T)
78L	pk.3-4-1	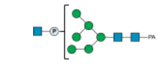	3.11	HPO3	(45T)

Table S3 Continued.

No.	Peak. No. (ODS)	Deduced glycan structure[a)]	Relative amounts[b)]	Notes[c)]	Presence in lung (No. in lung)
79T	pk.1-34-1	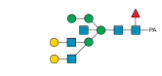	3.40		(146L)
80T	pk.3-18-2	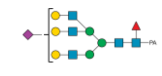	3.31	α2,6-Sia	
81T	pk.1-27-2	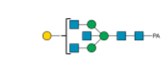	3.29		(55L)
82T	pk.5-14-1	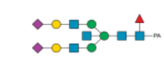	3.15	α2,3-Sia, α2,6-Sia	(87L)
83T	pk.8-12-1	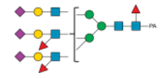	3.07	sLe^{X}, sLe^{X} α2,6-Sia	
84T	pk.3-33-1	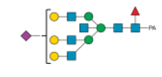	3.04	α2,6-Sia	(39L)
85T	pk.1-15-2	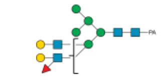	3.03	Le^{X}	
86T	pk.3-9-2	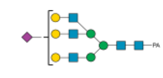	2.96	α2,6-Sia	(156L)
87T	pk.7-1-1	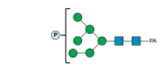	2.95	HPO3	(53L)
88T	pk.8-13-1	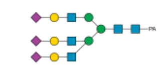	2.87	α2,3-Sia, α2,6-Sia, α2,6-Sia	(152L)
89T	pk.5-12-1	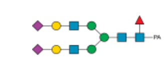	2.76	α2,3-Sia, α2,3-Sia	(31L)
90T	pk.6-10-1	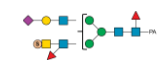	2.62	LacdiNAc with Fuc and SO3	
91T	pk.1-15-1	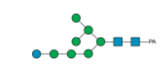	2.40		
92T	pk.8-6-1	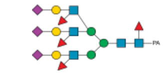	2.18	sLe^{X}, sLe^{X}, sLe^{X}	
93T	pk.1-25-2	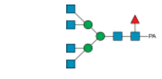	2.13		
94T	pk.3-7-2	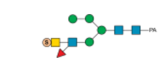	2.08	LacdiNAc with Fuc, SO3	
95T	pk.3-33-2	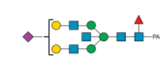	2.06	α2,3-Sia	(77L)
96T	pk.1-16-2	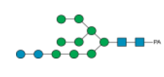	2.04		
97T	pk.8-8-1	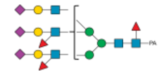	2.02	sLe^{X}, sLe^{X} α2,6-Sia	
98T	pk.3-5-1	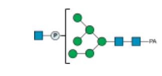	2.01	HPO3	(172L)

No.	Peak. No. (ODS)	Deduced glycan structure[a)]	Relative amounts[b)]	Notes[c)]	Presence in trachea (No. in trachea)
79L	pk.5-7-1	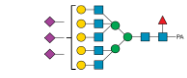	2.94		
80L	pk.1-30-2	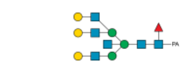	2.94		(52T)
81L	pk.3-36-1	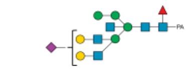	2.91	α2,6-Sia	(110T)
82L	pk.3-23-1	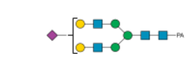	2.88	Standard d α2,3-Sia	
83L	pk.1-18-1	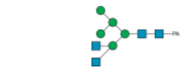	2.87		
84L	pk.3-24-1	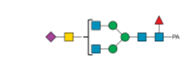	2.85	sLacdiNAc with α2,6-Sia	
85L	pk.3-22-2	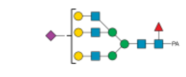	2.84	α2,3-Sia	
86L	pk.4-16-1	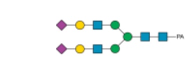	2.79	α2,3-Sia, α2,6-Sia	(66T)
87L	pk.4-28-1	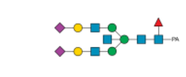	2.77	α2,3-Sia, α2,6-Sia	(82T)
88L	pk.1-22-2	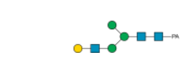	2.69		
89L	pk.6-5-1	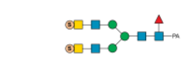	2.69	LacdiNAc with SO3, LacdiNAc with SO3	
90L	pk.3-27-2	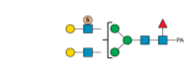	2.58	LacNAc with SO3	
91L	pk.5-8-1	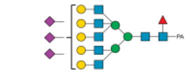	2.53		
92L	pk.3-25-5	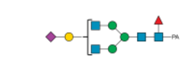	2.43	α2,6-Sia	
93L	pk.5-9-1	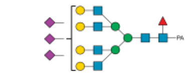	2.40		
94L	pk.4-18-1	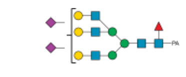	2.26		
95L	pk.4-23-1	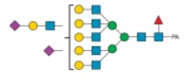	2.25	sLacNAc repeat	
96L	pk.1-34-1	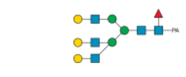	2.21		
97L	pk.3-5-1	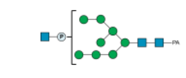	2.14	HPO3	(70T)
98L	pk.5-5-1	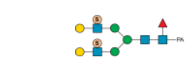	2.14	LacNAc with SO3, LacNAc with SO3	

Table S3 Continued.

No.	Peak. No. (ODS)	Deduced glycan structure[a)]	Relative amounts[b)]	Notes[c)]	Presence in lung (No. in lung)
99T	pk.6-1-1	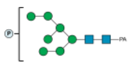	1.93	HPO3	(165L)
100T	pk.3-14-2	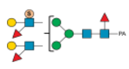	1.68	Le^{X},<br>Le^{X} with SO3	
101T	pk.8-1-1	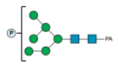	1.65	HPO3	(155L)
102T	pk.5-11-2	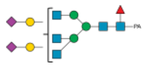	1.63	α2,6-Sia, α2,6-Sia	
103T	pk.5-6-2	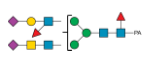	1.63	sLe^{X}<br>sLacdiNAc with α2,6-Sia	
104T	pk.3-11-3	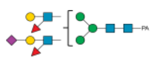	1.47	Le^{X}, sLe^{X}	
105T	pk.8-10-1	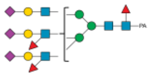	1.46	sLe^{X}, sLe^{X}<br>α2,6-Sia	
106T	pk.3-13-2	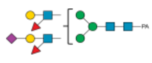	1.45	Le^{X}, sLe^{X}	
107T	pk.3-10-2	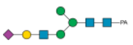	1.37	α2,6-Sia	(162L)
108T	pk.8-14-1	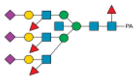	1.32	sLe^{X}, sLe^{X}, sLe^{X}	
109T	pk.3-16-3	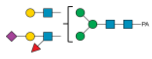	1.29	sLe^{X}	
110T	pk.3-31-1	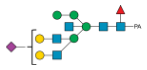	1.18	α2,6-Sia	(81L)
111T	pk.6-5-1	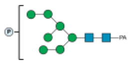	0.62	HPO3	(54L)

No.	Peak. No. (ODS)	Deduced glycan structure[a)]	Relative amounts[b)]	Notes[c)]	Presence in trachea (No. in trachea)
99L	pk.5-6-1	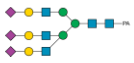	2.11		
100L	pk.4-30-1	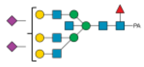	2.04		
101L	pk.3-15-1	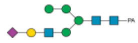	2.02	α2,3-Sia	
102L	pk.1-19-2	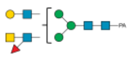	1.95	LacdiNAc with Fuc	
103L	pk.3-21-2	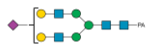	1.87	Standard b<br>α2,6-Sia	
104L	pk.4-27-1	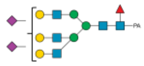	1.83		
105L	pk.5-10-1	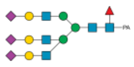	1.81		
106L	pk.4-13-1	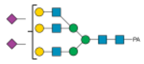	1.79		
107L	pk.4-21-1	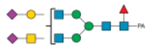	1.74	sLacdiNAc with α2,6-Sia,<br>α2,3-Sia	
108L	pk.1-29-2	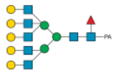	1.73		
109L	pk.3-37-1	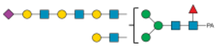	1.72	sLacNAc repeat with α2,3-Sia	
110L	pk.3-40-1	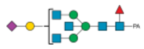	1.72	α2,3-Sia	
111L	pk.4-17-1	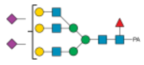	1.64		
112L	pk.3-32-2	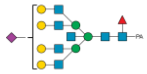	1.61	α2,6-Sia	
113L	pk.3-25-4	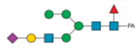	1.60	α2,3-Sia	
114L	pk.3-42-1	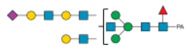	1.59	sLacNAc repeat with α2,3-Sia	
115L	pk.3-16-2	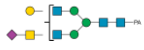	1.58	sLacdiNAc with α2,6-Sia	
116L	pk.3-14-1	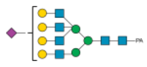	1.57	α2,3-Sia	
117L	pk.3-15-2	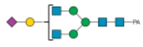	1.55	α2,6-Sia	
118L	pk.4-24-2	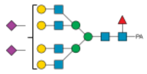	1.47		

Table S3 Continued.

No.	Peak. No. (ODS)	Deduced glycan structure[a)]	Relative amounts[b)]	Notes[c)]	Presence in lung (No. in lung)

No.	Peak. No. (ODS)	Deduced glycan structure[a)]	Relative amounts[b)]		Notes[c)]	Presence in trachea (No. in trachea)
119L	pk.3-11-1	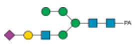	1.46		α2,6-Sia	(67T)
120L	pk.3-16-1	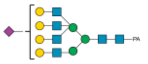	1.43		α2,3-Sia	
121L	pk.3-29-3	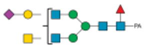	1.42		LacdiNAc, α2,6-Sia	
122L	pk.4-25-2	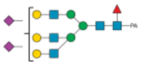	1.41			
123L	pk.1-16-2	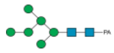	1.40			
124L	pk.1-21-1	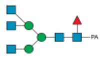	1.38			
125L	pk.1-12-3	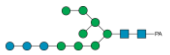	1.36			
126L	pk.3-28-2	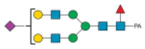	1.36		Standard f α2,6-Sia	
127L	pk.4-21-2	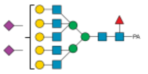	1.35			
128L	pk.3-32-1	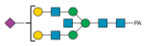	1.30		α2,3-Sia	
129L	pk.3-30-2	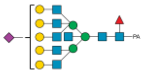	1.29		α2,6-Sia	
130L	pk.4-16-2	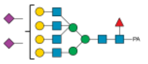	1.26			
131L	pk.3-38-2	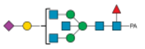	1.22		α2,6-Sia	
132L	pk.1-25-2	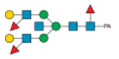	1.19		Le^X, Le^X	(2T)
133L	pk.3-25-3	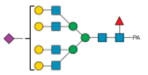	1.17		α2,6-Sia	
134L	pk.1-16-3	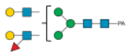	1.14		Le^X	
135L	pk.1-26-3	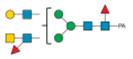	1.11		LacdiNAc with Fuc	
136L	pk.1-12-4	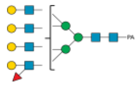	1.10		Le^X	
137L	pk.4-21-3	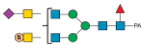	1.10		LacdiNAc with SO3, LacdiNAc with α2,6-Sia	
138L	pk.3-6-1	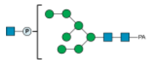	1.08		HPO3	(76T)

Table S3 Continued.

No.	Peak. No. (ODS)	Deduced glycan structure[a)]	Relative amounts[b)]	Notes[c)]	Presence in lung (No. in lung)

No.	Peak. No. (ODS)	Deduced glycan structure[a)]	Relative amounts[b)]	Notes[c)]	Presence in trachea (No. in trachea)
139L	pk.1-18-2	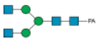	1.07	Standard I	
140L	pk.3-14-2	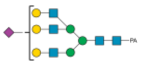	1.06	α2,3-Sia	
141L	pk.4-18-2	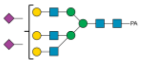	1.04		
142L	pk.3-38-3	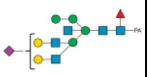	1.04	α2,3-Sia	
143L	pk.6-4-1	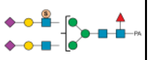	1.03	sLacNAc with SO3 and α2,6-Sia, α2,6-Sia	
144L	pk.1-18-3	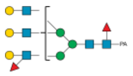	1.03	Le^X	(50T)
145L	pk.1-23-5	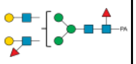	1.03	Le^X	(15T)
146L	pk.1-39-1	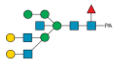	1.00		(79T)
147L	pk.1-40-1	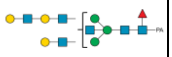	1.00	LacNAc repeat	
148L	pk.4-28-2	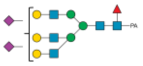	0.98		
149L	pk.4-29-1	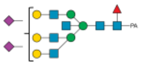	0.96		
150L	pk.3-38-1	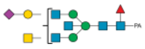	0.95	LacdiNAc, α2,6-Sia	
151L	pk.4-31-1	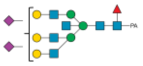	0.93		
152L	pk.5-8-2	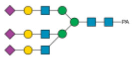	0.91		(88T)
153L	pk.3-28-4	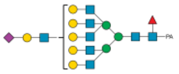	0.87	sLacNAc repeat with α2,3-Sia	
154L	pk.5-11-1	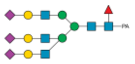	0.86		
155L	pk.5-2-1	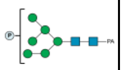	0.85	HPO3	(101T)
156L	pk.3-10-3	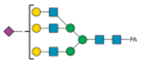	0.85	α2,6-Sia	(86T)
157L	pk.3-28-3	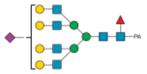	0.81	α2,3-Sia	
158L	pk.4-11-1	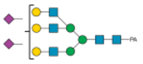	0.80		

Table S3 Continued.

No.	Peak. No. (ODS)	Deduced glycan structure[a)]	Relative amounts[b)]	Notes[c)]	Presence in lung (No. in lung)

No.	Peak. No. (ODS)	Deduced glycan structure[a)]	Relative amounts[b)]	Notes[c)]	Presence in trachea (No. in trachea)
159L	pk.5-12-1	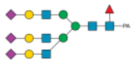	0.77		
160L	pk.6-6-1	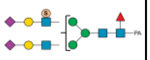	0.75	sLacNAc with SO3 and α2,3-Sia, α2,3-Sia	
161L	pk.3-13-2	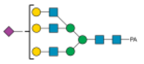	0.74	α2,3-Sia	
162L	pk.3-10-2	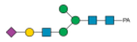	0.73	α2,6-Sia	(107T)
163L	pk.4-12-1	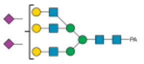	0.71		
164L	pk.5-4-1	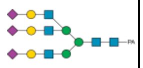	0.70		
165L	pk.4-1-1	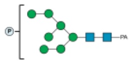	0.70	HPO3	(99T)
166L	pk.3-23-2	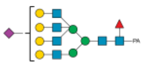	0.69	α2,3-Sia	
167L	pk.3-25-6	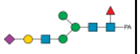	0.68	α2,3-Sia	
168L	pk.1-30-3	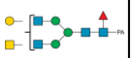	0.68	LacdiNAc	
169L	pk.1-21-3	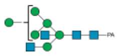	0.67		
170L	pk.3-21-3	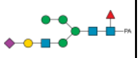	0.64	α2,6-Sia	
171L	pk.1-34-2	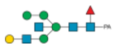	0.60		
172L	pk.3-8-1	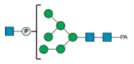	0.57	HPO3	(98T)
173L	pk.3-24-2	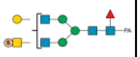	0.52	LacdiNAc with SO3	
174L	pk.3-34-2	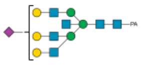	0.51	α2,6-Sia	
175L	pk.4-20-3	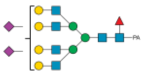	0.51		
176L	pk.4-22-2	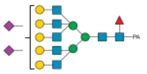	0.50		
177L	pk.4-29-2	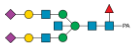	0.48	α2,3-Sia, α2,3-Sia	
178L	pk.4-10-1	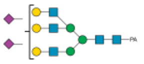	0.46		

Table S3 Continued.

No.	Peak. No. (ODS)	Deduced glycan structure[a)]	Relative amounts[b)]	Notes[c)]	Presence in lung (No. in lung)

No.	Peak. No. (ODS)	Deduced glycan structure[a)]	Relative amounts[b)]	Notes[c)]	Presence in trachea (No. in trachea)
179L	pk.4-22-3	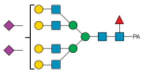	0.42		
180L	pk.1-21-2	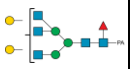	0.41		
181L	pk.5-13-1	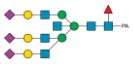	0.41		
182L	pk.4-22-4	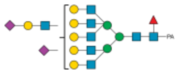	0.38	sLacNAc repeat	
183L	pk.5-8-3	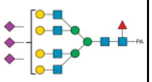	0.36		
184L	pk.4-9-1	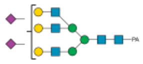	0.35		
185L	pk.5-6-2	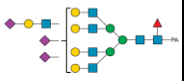	0.27	sLacNAc repeat	
186L	pk.5-1-2	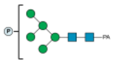	0.25	HPO3	
187L	pk.3-14-3	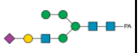	0.21	α2,3-Sia	
